# Signaling pathways of inflammation in CIA model of rheumatoid arthritis regulated by cannabichromene

**DOI:** 10.1186/s42238-026-00464-2

**Published:** 2026-06-24

**Authors:** Michaela Sklenárová, Monika Šteigerová, Petr Jelínek, Mykhaylo Bazyuk, Sara Merdita, Baraa Chaban, Jan Hlaváč, Daniel Stránský, Markéta Fučíková, Miroslav Šoóš, Martin Šíma, Ondřej Slanař

**Affiliations:** 1 Institute of Pharmacology, First Faculty of Medicine, Charles University and General University Hospital in Prague, Albertov 4, Prague, 128 00 Czech Republic; 2 Department of Chemical Engineering, Faculty of Chemical Engineering, University of Chemistry and Technology, Prague, Czech Republic; 3 Department of Gynaecology, Obstetrics and Neonatology, First Faculty of Medicine, Charles University and General University Hospital in Prague, Prague, Czech Republic

**Keywords:** Cannabinoids, Cannabichromene, Rheumatoid arthritis, CIA model, Inflammasome

## Abstract

**Background:**

Rheumatoid arthritis is a chronic autoimmune disease characterized by synovial inflammation, cytokine imbalance, and progressive joint destruction. The endocannabinoid system has emerged as a potential therapeutic target; however, the anti-inflammatory mechanisms of non-psychoactive cannabinoids such as cannabichromene (CBC) remain insufficiently defined. This study aimed to evaluate the anti-inflammatory effects of CBC in vitro and in a collagen-induced arthritis (CIA) rat model, with a focus on key inflammatory signaling pathways.

**Methods:**

CBC effects were assessed in LPS-stimulated HUVEC cells by qPCR analysis of inflammatory markers. In vivo, female Wistar rats were assigned to four groups: CIA + saline (placebo), CIA + CBC, CIA + methylprednisolone, and non-immunized controls receiving saline. Disease progression was evaluated using clinical scoring, paw thickness, and body weight. Synovial tissues and serum were analyzed by qPCR, Western blotting, and ELISA to assess cytokines, inflammasome components, and signaling pathways, including NF-κB and JAK/STAT.

**Results:**

CBC reduced TNF-α expression in vitro at low micromolar concentrations. In vivo, CBC significantly decreased arthritis scores compared to placebo and attenuated weight loss, although it did not significantly reduce paw swelling. Molecular analyses revealed downregulation of IL-6, STAT3, and IL-17 A, indicating suppression of the TNF–NF-κB–IL-6–STAT3–Th17 axis. CBC also significantly inhibited inflammasome components (NLRP3, NLRP1A, caspase-11). However, MMP-3 and MMP-9 levels were not significantly affected.

**Conclusions:**

CBC exhibits significant anti-inflammatory activity in vitro and in vivo by modulating key cytokine and inflammasome pathways. While its effects on structural joint damage markers were limited, CBC represents a promising candidate for inflammatory arthritis therapy, warranting further investigation.

**Supplementary Information:**

The online version contains supplementary material available at https://doi.org/10.1186/s42238-026-00464-2.

## Introduction

Rheumatoid arthritis (RA) is a prevalent chronic autoimmune and inflammatory disease that primarily targets synovial joints, leading to excessive synovial proliferation and progressive bone destruction. Although current therapeutic approaches can alleviate symptoms and slow the disease progression, they remain palliative rather than curative. Despite decades of intensive research, the precise etiology of RA is still not fully understood. It is, however, widely accepted that a complex interplay between genetic predisposition, environmental triggers, and immune dysregulation contributes significantly to disease onset and progression (Smolen and Aletaha 2015).

A hallmark of RA is the disruption of the balance between pro-inflammatory and anti-inflammatory cytokines. Central mediators such as TNF-α, IL-6, and IL-17 promote synovial hyperplasia, angiogenesis, and osteoclast activation (McInnes and Schett 2007). These processes culminate in the formation of pannus – an invasive granulation tissue that progressively erodes cartilage and bone – eventually resulting in joint deformities and systemic complications, including cardiovascular and metabolic comorbidities.

The collagen-induced arthritis (CIA) model remains one of the most widely utilized preclinical models for RA research. CIA exhibits a chronic, progressive disease course when induced with autologous collagen in rats. Moreover, both RA and CIA are characterized by the presence of inflammatory infiltration of macrophages, fibroblasts, T cells, and granulocytes. These similarities suggest that both disorders are driven by a combination of delayed-type hypersensitivity and immune complex-mediated inflammation (Holmdahl et al. 1989, Steigerova et al. 2023).

The endocannabinoid system (ECS) is an essential regulator of numerous physiological processes, including nociception, appetite, emotional responses, memory, and immune homeostasis. Consequently, pharmacological modulation of the ECS has emerged as a promising therapeutic strategy for chronic inflammatory diseases such as RA. However, the intricate relationship between ECS signaling and the underlying immunopathological mechanisms of RA remains incompletely defined.

Experimental studies in both animal models and human subjects have demonstrated that activation of the CB2 cannabinoid receptor exerts immunosuppressive effects by downregulating TNF-α production and modulating Th1-mediated immune responses (Malfait et al. 2000). Furthermore, emerging evidence indicates that CB2 receptor agonists may attenuate joint inflammation, cartilage degradation, and bone erosion through the regulation of MAPK and NF-κB signaling pathways in CIA models (Turcotte et al. 2016).

Previous studies on potential anti-inflammatory effect of cannabinoids, focused on cannabidiol (CBD), or cannabigerol (CBG) (Sklenarova et al. 2023, Steigerova et al. 2025, Jelinek et al. 2024). Nonetheless, other non-psychoactive cannabinoids, such as cannabichromene (CBC), may also represent compounds of interest.

CBC is a non-psychoactive phytocannabinoid that has gained increasing attention for its potential therapeutic effects on pain, inflammation, and arthritis. Preclinical studies show that CBC exerts anti-inflammatory and analgesic actions mainly through modulation of non-CB1/CB2 targets, including TRPV1 (transient receptor potential vanilloid type 1) and TRPA1(transient receptor potential ankyrin 1) channels, which are key mediators in pain and inflammatory pathways (DeLong et al. 2010, Romano et al. 2013). Wirth et al. first showed CBC’s anti-edematous effects in rats, and more recent study confirmed CBC’s suppression of inflammatory gene expression in macrophages (Wirth et al. 1980, Gojani et al. 2023). Collectively, these findings highlight CBC as a modulator of nociceptive and inflammatory signaling, suggesting therapeutic promise for arthritis and chronic inflammatory pain.

Evidence from prior research indicates that CBC may exert anti-inflammatory effects, yet the molecular mechanisms and specific signaling pathways through which it mediates these actions are not well elucidated. To address this gap, the present in vivo study examined the anti-inflammatory efficacy of CBC CIA rat model and explored its influence on intracellular signaling networks associated with the inflammatory pathophysiology of CIA and RA.

## Methods

### Isolation and Cultivation of HUVEC cells

HUVEC cells were isolated from umbilical cords by our modification of standard method by Jaffe et al. (Jaffe et al. 1973). Briefly, umbilical cord was obtained with written informed consent and institutional ethics committee approval from the Department of Gynecology, Obstetrics and Neonatology, General University Hospital in Prague. Upon receipt, the umbilical vein was flushed with PBS until clear fluid flowed out. One end of the vein was closed with hemostatic forceps and a three-way stopcock secured with a zip tie was inserted into the other end. The vein was filled with 0.1% (w/v) collagenase type I prepared in serum-free DMEM/F-12 medium pre-warmed to 37 °C and the cord was incubated in a 37 °C PBS bath for 15 min. The fluid from the vein was poured into a 50 mL centrifuge tube, the vein was rinsed twice with PBS into the same tube, and the cell suspension was centrifuged at 350 × g for 10 min. Supernatant was poured away and the cell pellet was resuspended in 14 mL DMEM/F-12 medium supplemented with 10% (v/v) fetal bovine serum, penicillin–streptomycin (100 U/mL and 100 µg/mL), 10 mM HEPES, endothelial cell growth supplement (50 µg/mL), heparin (100 µg/mL) and hydrocortisone (725 ng/mL) and plated into 100 mm culture dish pre-coated with a thin non-gelling layer of Geltrex™ to promote adhesion.

After 24 h, the medium was replaced with fresh medium to remove non-adherent cells. Thereafter, medium was changed every 48 h. At ~ 80% confluence, cells were passaged using 0.05% trypsin–EDTA. HUVEC from passage 3 were seeded at 25,000 cells per well in 96-well culture plates pre-coated with a thin layer of Geltrex™ and allowed to adhere overnight. On the following day, test compounds were added in culture medium for 2 h incubation. Subsequently, LPS was added to a final concentration of 100 ng/mL.

### qPCR analysis of HUVEC cells

Total RNA was isolated from cells harvested 2 h post-LPS stimulation, and cDNA was synthesized as previously described (Stransky et al. 2025). Gene expressions were quantified using custom probe-based assays. Primers (Generi Biotech, Hradec Králové, Czech Republic) and dual-labeled hydrolysis probes QSY/QSY2 (Thermo Fisher Scientific, USA, cat. No. 4482778/A56900) were designed for target genes (*TNF*,* IL1A*) and reference genes (*HPRT1*,* TBP*,* YWHAZ*; sequences listed in Table 1). Assays were validated for cDNA specificity (> 100-fold signal reduction in no-RT controls) and amplification efficiency (90–110%). The qPCR was performed on a QuantStudio 3 Real-Time PCR system (Thermo Fisher Scientific, USA) under manufacturer-recommended conditions. All runs included no-reverse-transcriptase and no-template controls to validate the specificity of the amplification. Relative gene expression was calculated using the comparative 2⁻ΔΔCT method. The sample size for each experiment was defined as the number of biological replicates: *n* = 6.

**Table 1:** Primer sequences used in qPCR analysis of HUVEC cells

Official gene symbol	Forward primer 5’-3’	Reverse primer 5’-3’	Probe 5’-3’
TBP	CAAGAACTTAGCTGGAAAACCC	GATAAGAGAGCCACGAACCAC	CACAGGAGCCAAGAGTGAAGAACAGT
HPRT1	GTATTCATTATAGTCAAGGGCATATCC	AGATGGTCAAGGTCGCAAG	TGGTGAAAAGGACCCCACGAAGT
YWHAZ	TCAGTTACAGACTTCATGCAGG	TCCCTCAAACCTTGCTTCTAG	AGTTTGGCCTTCTGAACCAGCTCATT
TNF	TCAGCTTGAGGGTTTGCTAC	TGCACTTTGGAGTGATCGG	AGATGATCTGACTGCCTGGGCC
IL1A	TCTTCATCTTGGGCAGTCAC	GCTGCTGCATTACATAATCTGG	TGAAGCAGTGAAATTTGACATGGGTGC

### Animals

The female Wistar rats (250–350 g) were obtained from Velaz (Prague, Czech Republic) and housed under controlled laboratory conditions (22 ± 2 °C, 50 ± 10% relative humidity, 12-hours light/dark cycle) with ad libitum access to food and water. All experimental procedures complied with the ethical guidelines of the First Faculty of Medicine, Charles University. The animal experimental protocol was approved by the Ministry of Education, Youth, and Sports of the Czech Republic (approval No. MSMT-26838/2021-4).

### Reagents

Bovine type II collagen and incomplete Freund’s adjuvant (Chondrex) were used for induction of CIA. Methylprednisolone sodium succinate (SOLU-MEDROL 40 mg, Pfizer), CBC nanoemulsion and 0.9% saline (placebo) were used for treatment. The tested CBC formulation was prepared as published previously (Rysanek et al. 2025). CBC (Pharmabinoid) was first dissolved in the oil phase, which was composed of 50 wt% Kolliphor EL (BASF, Germany), 30 wt% diethylene glycol monoethyl ether (Gattefossé, France), and 20 wt% propylene glycol monocaprylate (Gattefossé, France). Water was then added dropwise to the oil mixture at a 4:1 ratio (water to oil, by weight) while stirring gently before use. The final CBC concentration was 15 mg/mL. The resulting nanoemulsion had a particle size of 32 nm, as determined by dynamic light scattering (LS Instruments, Switzerland).

### Design of experiment

**Fig. 1:**
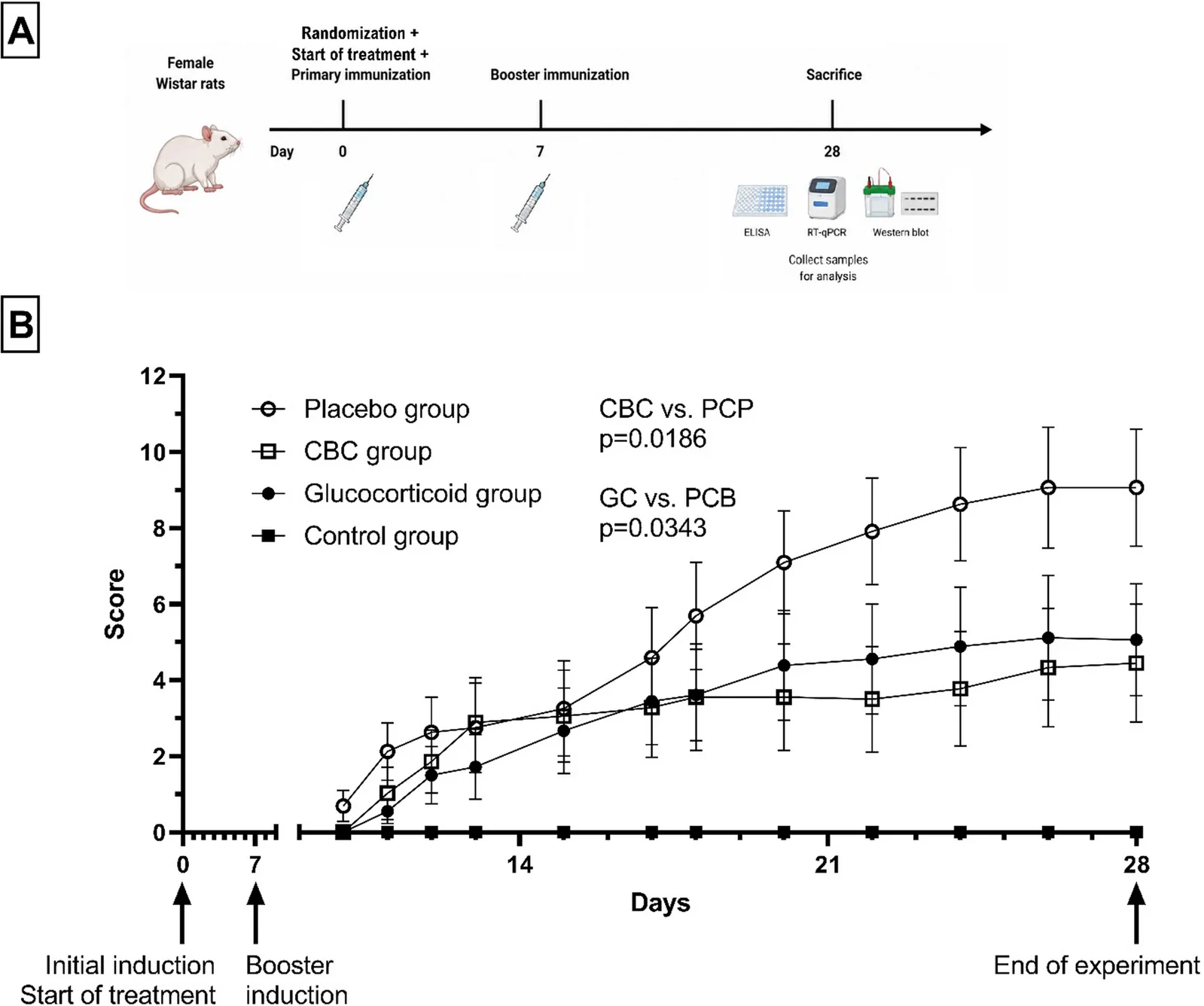
Illustration of the collagen-induced arthritis (CIA) experimental design (**A**) and clinical progression of CIA expressed as mean ± SD arthritic score in rats (**B**). CBC: cannabichromene group (*n* = 9), GC: glucocorticoid group (*n* = 9), CO: control group (*n* = 6), PCB: placebo group (*n* = 8)

Collagen emulsion for the induction of CIA was prepared following the Chondrex protocol. The emulsion was produced by mixing incomplete Freund’s adjuvant with bovine type II collagen dissolved in 0.05 M acetic acid (Chondrex 2015). Adult female rats received a subcutaneous injection of 0.2 mL of the emulsion at the base of the tail on day 0, followed by a booster injection of 0.1 mL on day 7. Control animals were administered with an equivalent volume of saline. After 30 days of treatment, all animals were euthanized using T-61 (Intervet International B.V., the Netherlands). The timeline of CIA induction, treatment administration, and sample collection is presented in Fig. 1A.

On day 0, rats were assigned identification numbers and randomly allocated to four experimental groups using a random number generator: placebo (PCB), cannabichromene (CBC), methylprednisolone (GC), and control (CO). The rats in the CBC group (*n* = 9) were treated with 15 mg of CBC in 1 mL of nanoemulsion once daily. The animals in the GC group (*n* = 9) were administered with 1 mL of solution containing 0.15 mg methylprednisolone daily. The rats in PCB (*n* = 9) and CO (*n* = 6) groups were given 1 mL of saline orally once per day.

Arthritis severity was evaluated every other day using a standardized clinical scoring system. A score of 0 indicated no visible inflammation; 1 represented swelling or redness in one joint; 2 denoted swelling or redness in multiple joints; 3 corresponded to swelling of the entire paw; and 4 indicated severe swelling, erythema, and stiffness affecting the ankle and distal digits. The total clinical score per animal was obtained by summing the scores for all four limbs, with a maximum possible score of 16. Ankle edema was measured using digital calipers on both hind limbs at baseline (day 0) and at the end of the study (day 28).

After 28 days of treatment, all animals were sacrificed, and blood samples along with synovial membrane tissues were collected. Cytokine and related gene expression levels were quantified using real-time polymerase chain reaction (RT-PCR), and protein expression was analyzed via Western blotting (WB).

We analyzed the effect of CBC on the relative expression of a wide range of inflammatory, immune and cell signaling markers including cytokines (IL-1β, IL-6, IL-17, IL-18, IL-23, TNF-α), COX-2, FOXP-3, Toll-like receptors (TLR-2, TLR-3, TLR-4, TLR-5, TRL-7, TRL-8, TLR-9), NF-kB p65 subunit, inflammasome components (NLRP3, NLRP1A, NLRC4, AIM2, Caspase-1, Caspase-11, Gasdermin-D), the JAK-STAT pathway components (JAK-1, JAK-2, JAK-3, TYK-2, STAT-1, STAT-2, STAT-3, STAT-4, STAT-5 A, STAT-6, SOCS-3), matrix metalloproteinases (MMP-3, MMP-9) and RANKL, and markers of apoptosis (BCL, BAX, caspase-3).

### Sample preparation

Synovial membrane samples were preserved in RNAlater Stabilization Solution (Invitrogen Corporation, Carlsbad, USA) and stored at − 80 °C until further processing. Blood samples collected for ELISA analysis were centrifuged at 2000 × g for 10 min at 4 °C to obtain serum, which was subsequently aliquoted and stored at − 80 °C pending analysis.

### ELISA assay

Blood samples were taken on Day 28 to evaluate matrix metalloproteinase (MMP)-3 levels in serum. MMP-3 levels were determined according to manufacturer’s instructions [Rat MMP3 ELISA kit (ab270216; Abcam, UK)].

### qPCR analysis of synovial tissue samples

Frozen synovial samples were processed, and qPCR was performed as described previously (Steigerova et al. 2025). Primers were designed using Primer-BLAST (Ye et al. 2012). Relative gene expression levels were calculated using the 2⁻ΔΔCT method, with RPL13A, B2M, and HPRT1 used as internal reference genes for normalization.

### Western blot

The synovial samples were homogenized and lysed according to previously described (Steigerova et al. 2025). The following primary antibodies were used: anti-NLRP3 recombinant monoclonal antibody (MA5-32255, Invitrogen, USA; dilution 1:1,000), anti-TNF-α monoclonal antibody (60291-1-IG, Invitrogen, USA; dilution 1:2,000), anti-MMP-3 monoclonal antibody (66338-1-IG, Invitrogen, USA; dilution 1:1,000), anti-NF-κB p65/RelA monoclonal antibody (A22331, ABclonal, Germany; dilution 1:10,000), anti-phospho-NF-κB p65 (Ser536) monoclonal antibody (MA5-15160, Invitrogen, USA; dilution 1:1,000), and anti-β2-microglobulin monoclonal antibody (59035, Cell Signaling Technology, USA; dilution 1:1,000). The band intensities of the target proteins were quantified using ImageJ software (National Institutes of Health, Bethesda, USA) and normalized to the respective housekeeping protein (β-2-microglobulin), followed by normalization to the corresponding negative control group.

### Statistical analysis

All data were expressed as the mean ± standard deviation (SD). The significance of the differences between groups was analyzed by one-way ANOVA with Tukey’s multiple comparisons test (comparison of all study groups) or by Student t-test (comparison of two selected groups). All statistical analyses were performed using GraphPad Prism 8.2.1 software (GraphPad Inc., La Jolla, USA) and p-value ≤ 0.05 was considered statistically significant.

## Results

### In vitro effect of CBC on expression of inflammatory markers in HUVEC cells

**Fig. 2:**
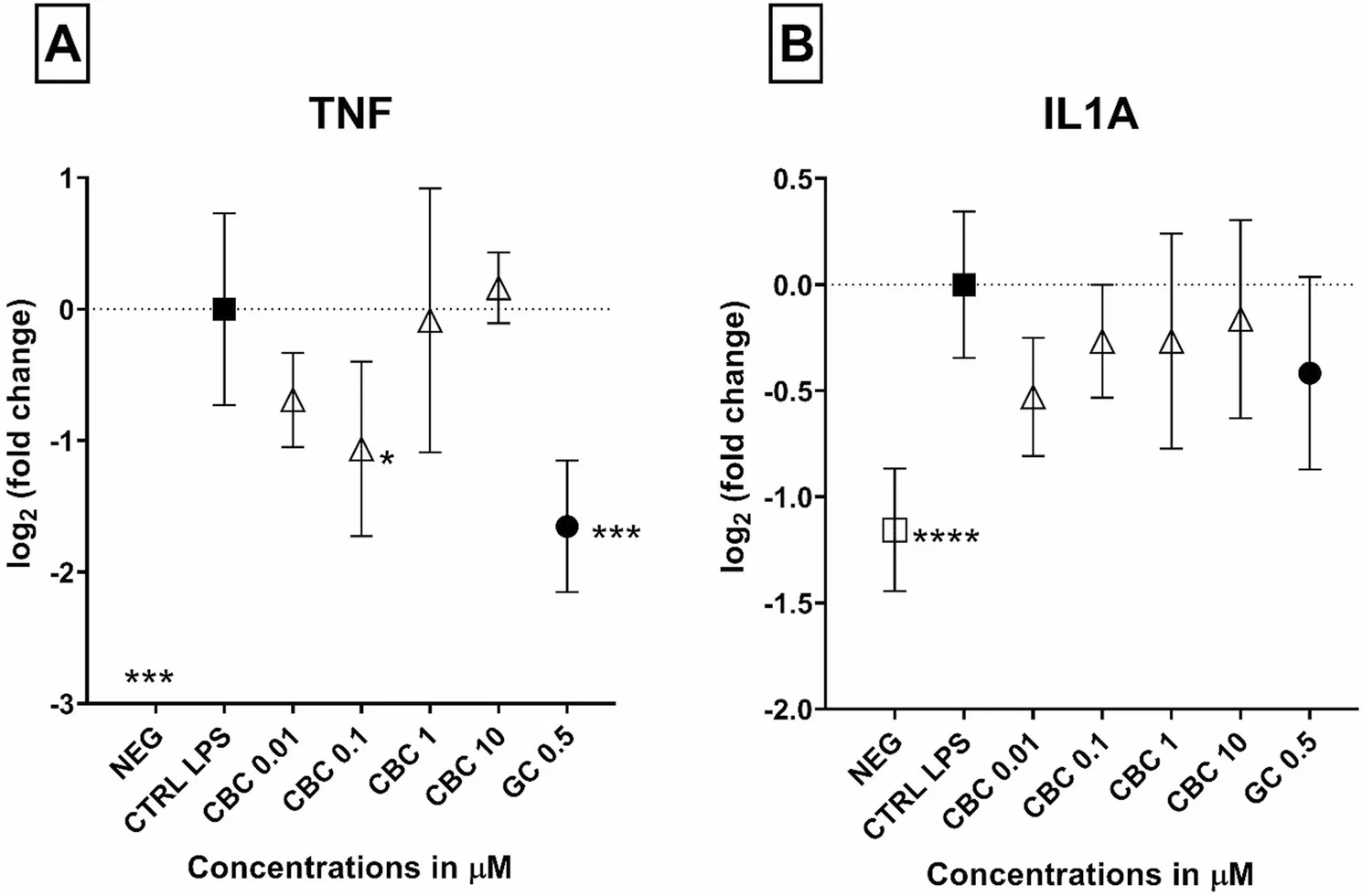
In vitro effects of cannabichromene on relative gene expression of target genes (**A**, **B**) in HUVEC cells. Each graph shows the relative expression of one target gene in the cannabichromen (CBC) and glucocorticoid group (GC) versus the placebo group (CTRL LPS) 2H after LPS stimulation. Results are expressed as log2 of fold change compared to placebo group, data represents the mean ± SD (*n* = 6). Statistical significance is indicated by *(*p* ≤ 0.05), ***(*p* ≤ 0.001), and ****(*p* ≤ 0.0001)

Compared to the placebo group (CTRL LPS), CBC reduced TNF-α expression primarily at lower concentrations with a statistically significant effect at 0.1 µM (*p* = 0.04) (Fig. 2). Regarding IL-1 A, CBC also demonstrated a trend toward suppression at this range of concentrations.

### Effects of the treatment on progression of CIA

Clinical scores are shown in Fig. 1B; ankle width and body weight are summarized in Table 2. PCB group showed significantly lower body weight (*p* = 0.0032), higher arthritis score (*p* < 0.0001) higher width of paws (*p* = 0.001) and higher serum MMP-3 (*p* < 0.0001) compared to CO group, indicating that the CIA model was induced successfully. The arthritic score in the CBC group was significantly lower compared to the PCB group (*p* = 0.02), although no significant reduction of width of paws compared to PCB group was noted (*p* = 0.34).

**Table 2:** Mean ± SD values of ankle width, weight and serum MMP-3 levels in rats

	Ankle width (mm)				Weight (g)		MMP-3 (ug/mL)
	Day 0		Day 28		Day 0	Day 28	Day 28
	Left hindlimb	Right hindlimb	Left hindlimb	Right hindlimb			
PCB	5.0 ± 0.3	5.1 ± 0.3	8.1 ± 1.6	8.9 ± 1.4	297.4 ± 14.1	249.6 ± 14.0	529.93 ± 177.7
CBC	5.0 ± 0.2	4.9 ± 0.3	7.4 ± 1.7	7.2 ± 1.8	298.0 ± 28.6	262.6 ± 36.3	399.27 ± 204.9
GC	5.2 ± 0.3	5.1 ± 0.3	7.7 ± 1.9	6.9 ± 2.0	298.4 ± 15.4	248.7 ± 18.3	415.47 ± 154.4
CO	5.0 ± 0.2	4.9 ± 0.3	5.1 ± 0.4 ***	5.1 ± 0.3 **	298.7 ± 6.7	295.8 ± 9.5 **	33.67 ± 3.2 ****

Statistically significant difference vs. PCB group is indicated by **(*p* ≤ 0.01), ***(*p* ≤ 0.001), and ****(*p* ≤ 0.0001)

*PCB* Placebo group (*n* = 8), *CBC* Cannabichromene group (*n* = 9), *GC* Glucocorticoid group *GC*(*n* = 9), *CO* Control group (*n* = 6)

The arthritic score in the GC group was significantly lower than in the placebo group (*p* = 0.03). The width of paws and serum MMP-3 levels in the GC group were not significantly lower on day 28 compared to PCB (*p* = 0.38 and *p* = 0.49, respectively) (Table 2).

Significant weight loss was noted in all groups (PCB, CBC, GC) compared to the CO group (*p* = 0.003, *p* = 0.034, *p* = 0.002, respectively); the CBC group experienced the least weight reduction (Table 2).

### Effect of CBC on TNF-α → NF-κB → IL-6 axis in synovial tissue

**Fig. 3:**
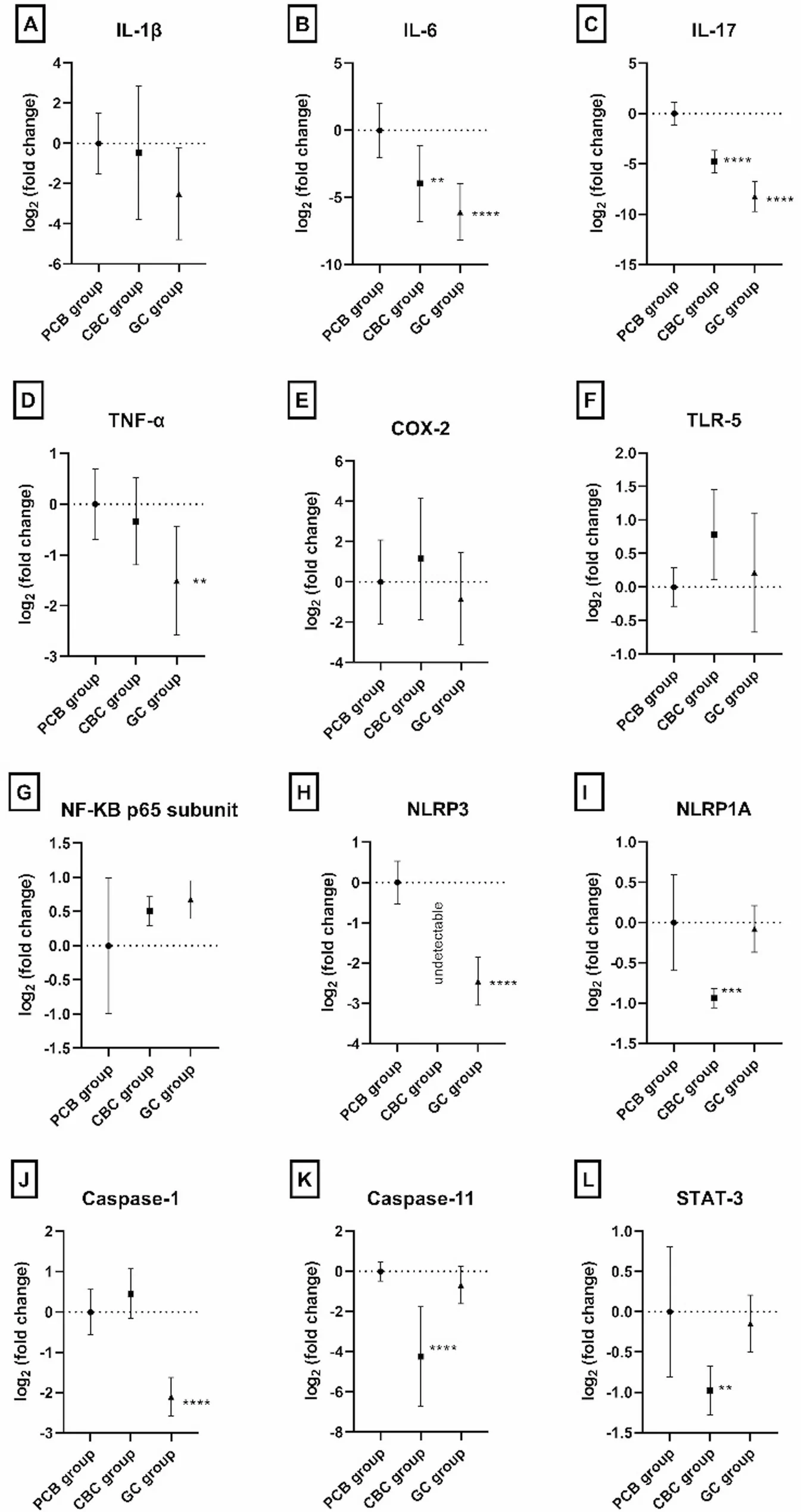
Effects of cannabichromene on relative gene expression of target genes (**A - L**) in synovial tissue. Each graph shows the relative expression of one target gene in the cannabichromen (CBC) and glucocorticoid group (GC) versus the placebo group (PCB). Results are expressed as log2 of fold change compared to PCB group, data represents the mean ± SD (PCB *n* = 8, CBC *n* = 9, GC *n* = 9). Statistical significance is indicated by **(*p* ≤ 0.01), ***(*p* ≤ 0.001), and ****(*p* ≤ 0.0001)

Compared to the PCB group, the CBC group showed a significant reduction in the relative expression levels of IL-6 and STAT3 (*p* = 0.0060, *p* = 0.0030, respectively) with a non-significant trend toward suppression of TNF-α (*p* = 0.7265) (Fig. 3B, D and L). The relative mRNA expression the NF-κB p65 subunit was not affected by CBC (Fig. 3G), however, Western blot analysis revealed a trend toward reduced levels of p-NF-κB in the CBC group compared to the PCB group (*p* = 0.22) (Fig. 5A), suggesting a potential decrease in NF-κB pathway activation. Since p-NF-κB is a marker of active transcriptional signaling involved in pro-inflammatory gene expression, this reduction indicates that the intervention may attenuate the inflammatory cascade characteristic of collagen-induced arthritis. Furthermore, total NF-κB protein expression was significantly reduced in the CBC group (*p* = 0.05) (Fig. 5A) compared to the PCB group, indicating that the treatment may not only inhibit NF-κB activation but also downregulate its overall expression.

This downregulation of key mediators suggests suppression of the TNF-driven NF-κB/STAT3 signaling cascade, which is known to regulate the production of inflammatory cytokines. Also, on the level of protein expression we observed the downregulation of TNF-α in the CBC and GC groups compared to PCB (*p* = 0.003, *p* = 0.002, respectively) (Fig. 5B). TNF-α and IL-6 are the major drivers of synovial inflammation. They activate fibroblast-like synoviocytes (FLS) and macrophages leading to joint swelling, pannus formation, and cartilage destruction. IL-6 and IL-23 activate STAT3, which promotes the differentiation of Th17 cells, linking this process to the next critical inflammatory axis.

### Effect on expression of IL-6 → STAT3 → IL-17 A axis

The relative expression of IL-17 A, the signature cytokine produced by Th17 cells, was significantly reduced in both the CBC and GC groups (*p* < 0,0001, *p* < 0,0001) compared to the PCB group (Fig. 3C). This decrease aligns with the suppressed levels of IL-6 and STAT3 (Fig. 3B and L), key drivers of Th17 cell differentiation. Th17 cells play a critical role in disease progression by secreting IL-17 A, which recruits neutrophils, promotes osteoclastogenesis leading to bone erosion, and amplifies the production of TNF and IL-6 (Yang et al. 2019). Consequently, lowering IL-17 A levels helps to mitigate joint inflammation and prevent bone damage.

### Effect on expression of matrix metalloproteinases MMP-3 and MMP-9

**Fig. 4:**
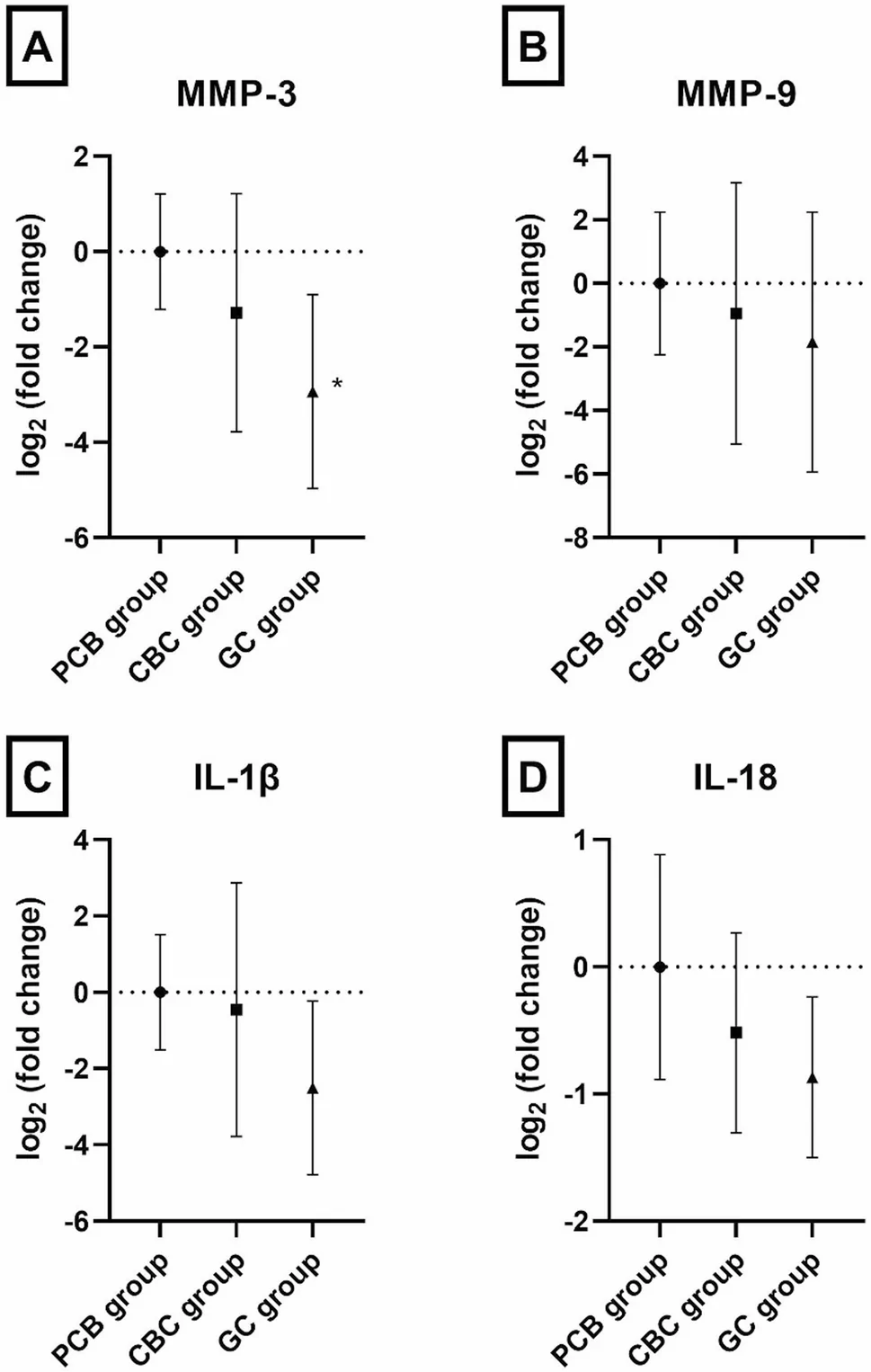
Effects of cannabichromene on relative gene expression of target genes (**A - D**) in synovial tissue. Each graph shows the relative expression of one target gene in the cannabichromen (CBC) and glucocorticoid group (GC) versus the placebo group (PCB). Results are expressed as log2 of fold change compared to PCB group, data represents the mean ± SD (PCB *n* = 8, CBC *n* = 9, GC *n* = 9). Statistical significance is indicated by *(*p* ≤ 0.05).

**Fig. 5:**
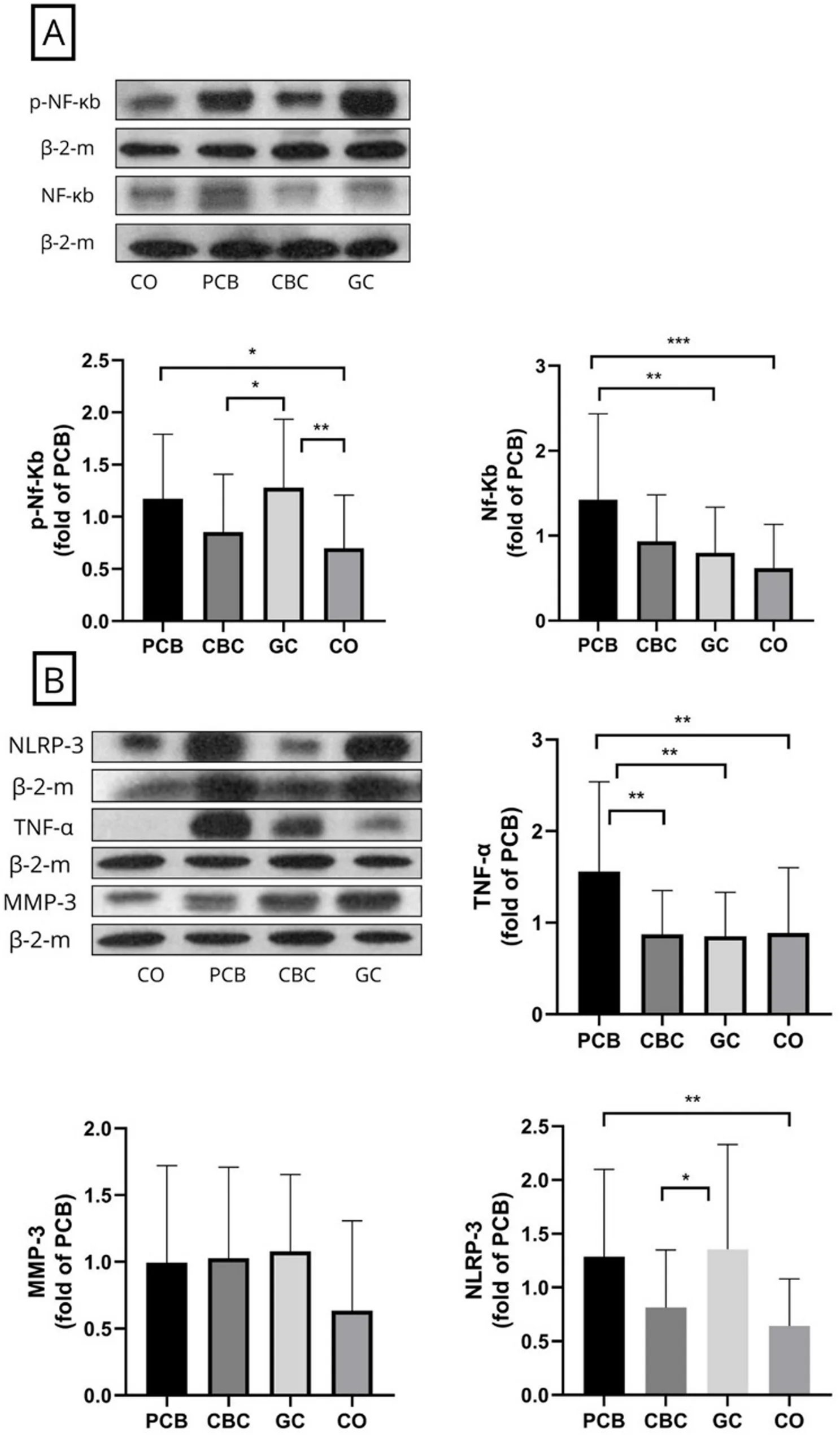
Proteins levels of p-NF-kB and NF-kB (**A**), and of TNF-α, MMP-3, and NLRP-3 (**B**) in the synovial membrane of CIA rats in the cannabichromene 15 mg (CBC), glucocorticoid (GC), and control groups (CO) versus the placebo group (PCB). Representative imagines of western blot protein bands in synovial membrane of CIA rats, Quantitative densitometry of proteins bands in synovial membrane of CIA rats β-2-m was used as a loading control. The data are expressed as the mean ± SD (CO *n* = 9, PCB *n* = 8, CBC *n* = 9, GC *n* = 9) *P < 0.05; **P < 0.01; ***P < 0.001 compared to placebo group

As shown in Fig. 4A and B, the expression of MMP-3 and MMP-9 expression was not markedly affected in the CBC group. These results are consistent with those from WB (Fig. 5A and B) and ELISA (Table 2) analyses.

### Effect on expression of inflammasome components (NLRP3, NLRP1A, and Caspase-11)

Inflammasome activation plays a central role in perpetuating chronic synovial inflammation (Jiang et al. 2021). In the current study, the expression of inflammasome-related genes – including NLRP3, NLRP1A, and caspase-11 – was markedly elevated in the PCB group, indicating increased inflammasome priming and activation (Fig. 3H, I and K). Treatment with CBC and GC significantly downregulated these key components. Specifically, CBC treatment reduced gene expression of NLRP1A (*p* = 0.001), NLRP3 (*p* < 0,0001) and caspase-11 (*p* < 0,0001) (Fig. 3H, I and K). WB analysis also revealed reduced protein expression of NLRP3 in the CBC-treated group (*p* = 0.09), suggesting potent inhibition of both canonical inflammasome signaling and pyroptotic pathways (Fig. 5). These findings indicate that CBC therapy may suppress inflammasome activation, likely secondary to decreased NF-κB signaling, leading to potential reduction of IL-1 and IL-18 expression. This inhibition may ultimately limit synovial inflammation and tissue destruction, contributing to the overall anti-inflammatory effect. The difference between the other markers did not reach statistical significance.

## Discussion

CIA was successfully established in experimental animals, as demonstrated by elevated clinical arthritis scores, increased paw thickness, and progressive body weight loss, consistent with previously published methodologies (Alabarse et al. 2018).

The daily dose of CBC (15 mg) administered in vivo was extrapolated from the concentration range (~ 0.1–10 µM) shown in vitro to attenuate TNF-α expression after LPS stimulation of HUVEC cells considering the pharmacokinetic performance of the nanoemulsion formulation. HUVEC cells were used as a standardized model of endothelial inflammatory activation, while LPS served as a robust inducer of cytokine production. The in vitro experiment was intended primarily for estimation of biologically active CBC concentrations rather than for direct modelling of the CIA synovial microenvironment. Our previous pharmacokinetic investigations revealed that oral administration of 15 mg CBC in 1 mL of the nanoemulsion employed in this study (bioavailability approximately 18%) produced a peak plasma concentration (C_max_) of about 12 ng/mL and an overall exposure (AUC) of roughly 117 ng·h/mL (Rysanek et al. 2025). This systemic exposure corresponds to the concentrations demonstrated to exert biological activity in vitro.

Administration of CBC resulted in a significant reduction in clinical scores compared with the PCB group. Although treatment with CBC and GC did not restore ankle volumes to those observed in healthy controls, the approximately 43% reduction in swelling relative to the placebo group is considered clinically meaningful in the CIA model.

Moreover, CBC-treated animals exhibited the smallest loss of body weight among all CIA-induced groups, indicating likely partial preservation of general health status, or analgetic action of CBC. Contrary to other phytocannabinoids that affect feeding behavior, primarily THC and to a lesser extent CBG, there is currently no robust experimental or clinical evidence that CBC has orexigenic (appetite-stimulating) effects (Kirkham and Williams 2001, Zagozen et al. 2021).

In the CIA model, treatment with CBC attenuated key inflammatory mediators compared with the PCB group. The observed reductions in IL-6, TNF-α, IL-17 A, STAT-3, NF-kB, NLRP3, NLRP1a, and caspase-11 indicate suppression of multiple inflammatory cascades, notably the TNF–NF-κB/IL-6–STAT3, Th17/IL-17 A, and inflammasome pathways. These results align with previous studies showing that inhibition of these axes mitigates joint inflammation and tissue destruction (Chen and Zhou 2015, Ding et al. 2023, Zheng et al. 2014).

Evidence on CBC efficacy in RA or CIA is extremely limited, only one mice CIA study has suggested a potential benefit of a CBC–CBD combination based on reduced joint swelling (Grogan et al. 2023). That study, however, did not report the specific effects of CBC on circulating cytokines, although it documented that CIA was associated with elevated serum concentrations of G-CSF, IL-6, IL-17, MIG, and TNF-α, which showed strong positive correlations with both arthritis severity and paw swelling. These associations support the clinical relevance of the CBC-mediated improvement observed in the present work and are consistent with independent data demonstrating that CBC reduces the expression of key pro-inflammatory cytokines such as IL-1β, IL-6, and TNF-α at the mRNA and protein levels in other models and pathophysiological situations.

In LPS-stimulated HaCaT cells, CBC led to significant reduction in both IL-8 and IL-12 expressions compared to LPS-treated controls (Tortolani et al. 2023). In RAW 264.7 macrophages, the strongest reductions of IL-1β, IL-6, TNF-α and NF-κB were observed at higher CBC concentrations (5–20 µM) (Hong et al. 2023). Similarly, in an atopic dermatitis model, where topical administration of CBC (0.1 mg/kg or 1 mg/kg) significantly decreased mRNA expression inflammatory mediators (IL-1β, IL-6, IL-17, IL-18) (*p* < 0.05), as well as protein expression of JAK-1, JAK-2, STAT-1, STAT-2, STAT-3, and STAT-6 (*p* < 0.05) (Kim et al. 2024). In contrast, in the Carrageenan-Induced Mouse Model, 4-day oral CBC treatment (10 mg/kg) did not significantly reduce IL-1β or IL-6, though it did reduce iNOS expression (Hong et al. 2023). After inhalation administration of CBC to mice with induced acute respiratory distress syndrome were observed by flow cytometry analysis of blood and lung that the expression levels of pro-inflammatory cytokines including IL-6, IL-17, and IFNγ in both tissues compared to the untreated counterparts were significantly reduced (*p* < 0.01) (Khodadadi et al. 2021).

Contrary to some of these observations, in our CIA model CBC did not significantly affect the expression of IL-1β, IL-18 or other components of JAK/STAT pathway, aside from STAT-3. This may be attributed to differences in the distinct immunological microenvironment between the atopic dermatitis, ARDS and CIA models.

The IL-6/STAT3 signaling axis plays a critical role in perpetuating chronic inflammation in CIA by promoting Th17 differentiation and sustaining cytokine amplification loops (Ogura et al. 2008). The marked decrease in IL-6 and STAT3 expressions in the CBC group suggests that the treatment disrupts this pro-inflammatory circuit, thereby limiting downstream IL-17 A production. Correspondingly, reduced IL-17 A expression has been shown to reflect dampened Th17 cell activity – a major contributor to joint inflammation and osteoclastogenesis in CIA (Adamopoulos and Bowman 2008).

The suppression of NLRP3, NLRP1A, and caspase-11 expression further indicates inhibition of inflammasome priming and non-canonical inflammasome activation, processes that are recognized contributors to exacerbated arthritis severity in RA and CIA (Yin et al. 2022, Yin et al. 2023).

CBC likely primarily acted at the level of inflammasome activation and cytokine processing/secretion, rather than at the level of cytokine gene transcription in synovial tissue, which could be an explanation for our observation that CBC treatment did not significantly affect the mRNA expression of these cytokines in the synovial tissue. In contrast, at lower concentrations, CBC moderately reduced IL-1 A expression in HUVEC cells, which differ from synovial leukocyte populations in their cytokine repertoire, signaling pathways, and inflammasome dependence.

Our findings that CBC may modulate inflammasome activity are supported by an in vitro study, in which CBC (2.5 µM and 5 µM) significantly decreased protein levels of NLRP3 and pro-IL-1β in LPS and ATP-stimulated THP-1 macrophages. Interestingly, in the same study, Caspase-1 protein levels were significantly increased in CBC 5 µM group, which is consistent with our observations of elevated Caspase-1 expression in synovial tissue from CBC-treated group (Gojani et al. 2023). Taken together, these results indicate that CBC treatment exerts broad anti-inflammatory activity.

Although CBC treatment significantly suppressed cytokine and inflammasome markers, MMP-3 and MMP-9 levels were not significantly reduced in the CBC-treated group. MMPs act as downstream effectors of joint destruction by degrading extracellular matrix components and promoting cartilage erosion (Grillet et al. 2023). One plausible explanation is that CBC preferentially targets upstream inflammatory pathways rather than distal tissue-remodeling cascades, implying that additional or combination strategies may be necessary to achieve robust protection against structural damage. Another possibility is a temporal disconnect, whereby meaningful downregulation of MMP expression and activity requires longer treatment duration or higher dosing before being captured at the tissue level. Consistent with this notion, our previous CIA studies with cannabidiol and cannabigerol, similarly failed to demonstrate a clear reduction in MMP levels despite anti-inflammatory efficacy. In contrast, selective CB2 receptor agonism may directly modulate MMPs secretion in CIA model: JWH133 significantly decreased MMP-3 expression in dose-dependent manner in cultured FLS (Fukuda et al. 2014). In an independent study, JWH133 significantly reduced MMP-9 protein expression in parallel with reduced inflammatory bone destruction in CIA mice.

These findings imply that CBC may effectively control inflammatory processes through modulation of the TNF–NF-κB/IL-6–STAT3–Th17–inflammasome network, leading to clinical improvement in CIA, but its effect on cartilage-degrading enzymes is limited. Combination therapy or extended treatment duration may be necessary to achieve joint protection.

Several limitations of this study should be acknowledged. First, the study was performed in a single preclinical model of arthritis, which may not fully reflect the complexity and heterogeneity of human rheumatoid arthritis. Second, mechanistic conclusions are based primarily on gene and protein expression analyses rather than direct functional pathway measurements. In particular, NF-κB activation was evaluated by separate assessment of phosphorylated and total NF-κB p65 protein levels rather than by calculation of the phospho/total protein ratio. Because total NF-κB p65 expression itself was altered across experimental groups, both parameters were presented independently to capture changes in protein abundance and activation state. In addition, some outcomes, including paw swelling, MMP-3, and MMP-9 expression, were not significantly affected by CBC treatment, suggesting that its anti-inflammatory effects may be selective and not uniformly reflected across all disease parameters. Future studies should further clarify the precise molecular mechanisms of CBC action, evaluate dose-response relationships, assess long-term safety, and determine whether prolonged administration or combination treatment strategies improve therapeutic efficacy and structural outcomes.

## Conclusion

CBC demonstrated anti-inflammatory activity in vitro and therapeutic effects in vivo in the CIA model. At physiologically relevant concentrations, CBC significantly reduced TNF-α expression in HUVEC cells and showed a suppressive trend for IL-1 A, suggesting that CBC may modulate early inflammatory signaling even at low micromolar concentrations. In vivo, CBC treatment significantly reduced arthritis severity and altered the expression of several inflammatory mediators. Specifically, CBC reduced IL-6, STAT3, and IL-17 A expression, findings consistent with modulation of pathways involved in synovial inflammation and Th17-associated responses. CBC also reduced the expression of inflammasome-related markers, including NLRP1A, NLRP3, and caspase-11, suggesting a potential effect on canonical and non-canonical inflammasome signaling. Together, these findings support the anti-inflammatory potential of CBC across multiple levels of inflammatory regulation and provide a basis for further investigation of its therapeutic relevance in inflammatory disease.

## Supplementary Information

Supplementary Material 1.

## Data Availability

The data that support the findings of this study are available from the corresponding author upon reasonable request.
