# Supplementary material for: Signaling pathways of inflammation in CIA model of rheumatoid arthritis regulated by cannabichromene
Source: J Cannabis Res. 2026 Jun 24;8:100. doi: 10.1186/s42238-026-00464-2 (PMC13555878; doi:10.1186/s42238-026-00464-2)
Supplement: Supplementary file 1 [file 42238_2026_464_MOESM1_ESM.pdf]

Supplementary data

Signaling pathways of inflammation in CIA model of rheumatoid arthritis regulated by cannabichromene

Michaela Sklenárová[1†], Monika Šteigerová[1†], Petr Jelínek[2], Mykhaylo Bazyuk[1], Sara Merdita[1], Baraa Chaban[1], Jan Hlaváč[1], Daniel Stránský[1], Markéta Fučíková[3], Miroslav Šoóš[2], Martin Šíma[1*], Ondřej Slanař[1*]

[1] Institute of Pharmacology, First Faculty of Medicine, Charles University and General University Hospital in Prague, Prague, Czech Republic.

[2] Department of Chemical Engineering, Faculty of Chemical Engineering, University of Chemistry and Technology, Prague, Czech Republic.

[3] Department of Gynaecology, Obstetrics and Neonatology, First Faculty of Medicine, Charles University and General University Hospital in Prague, Czech Republic.

†These authors contributed equally.

***Corresponding authors**

martin.sima@lf1.cuni.cz

ondrej.slanar@lf1.cuni.cz

Nf-Kb	b-2m
11,21,31,41-3x	
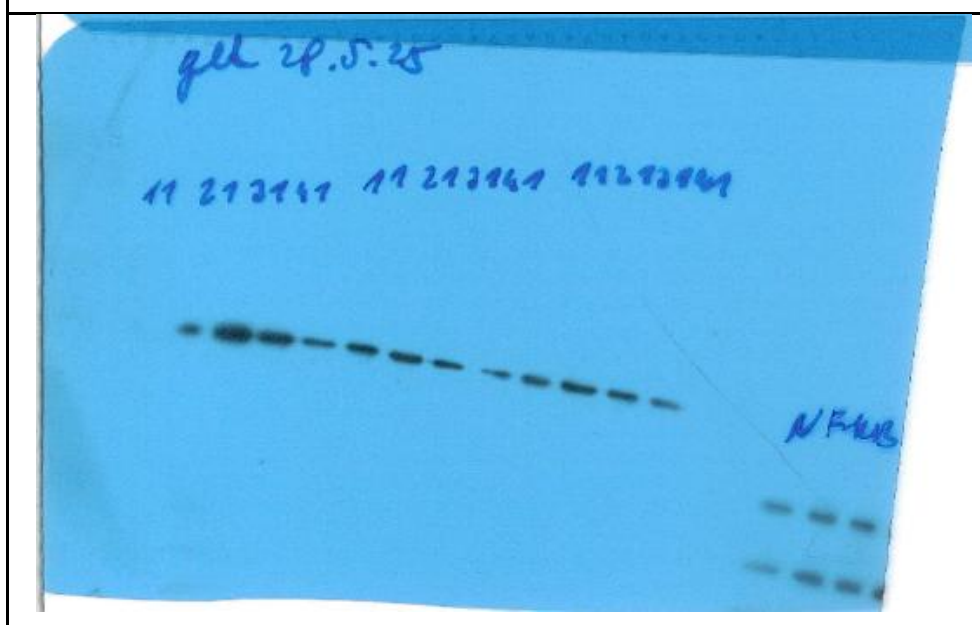	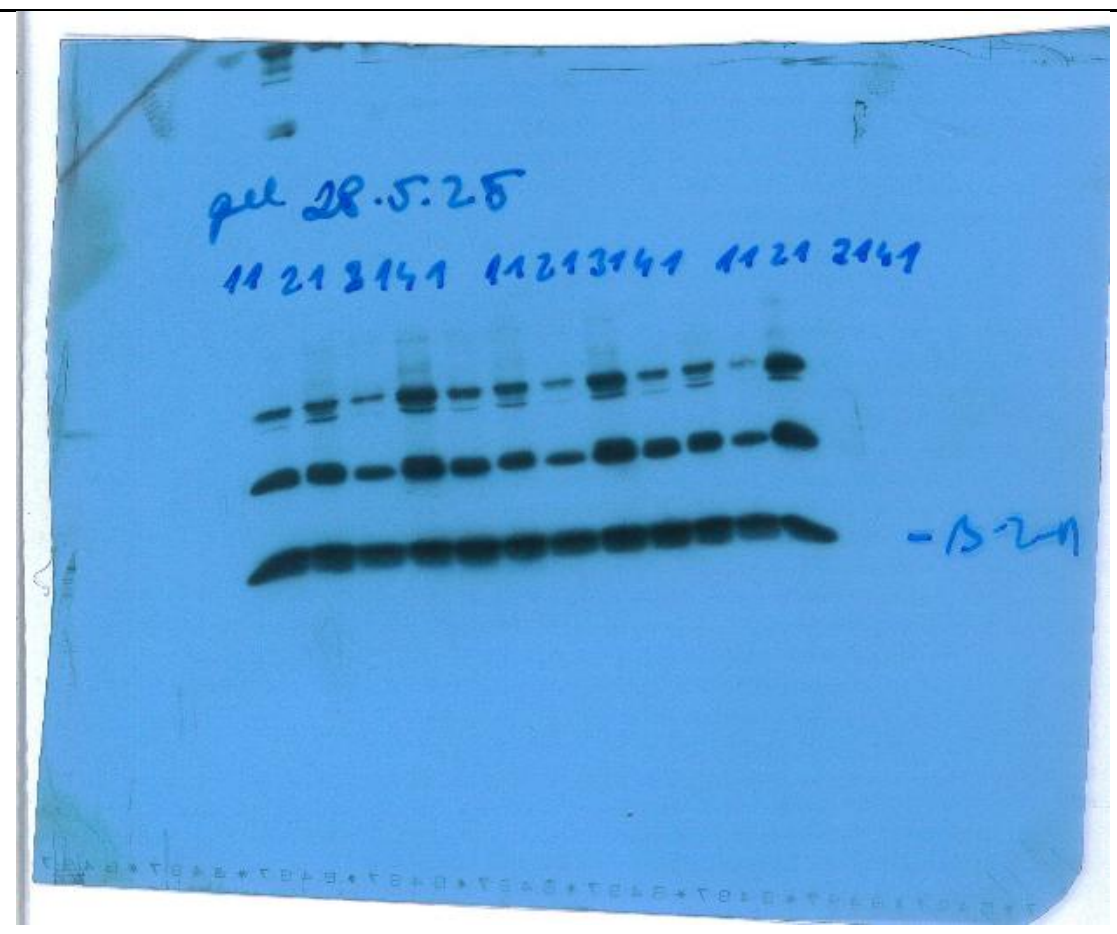
12,22,33,42-3x	
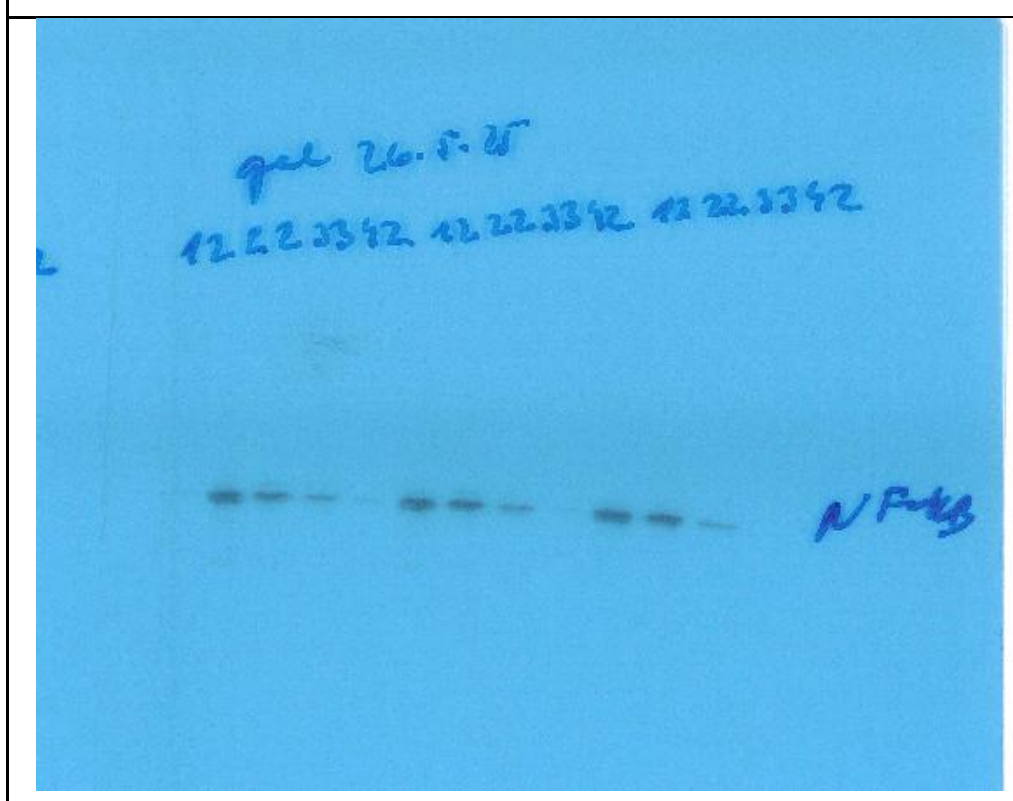	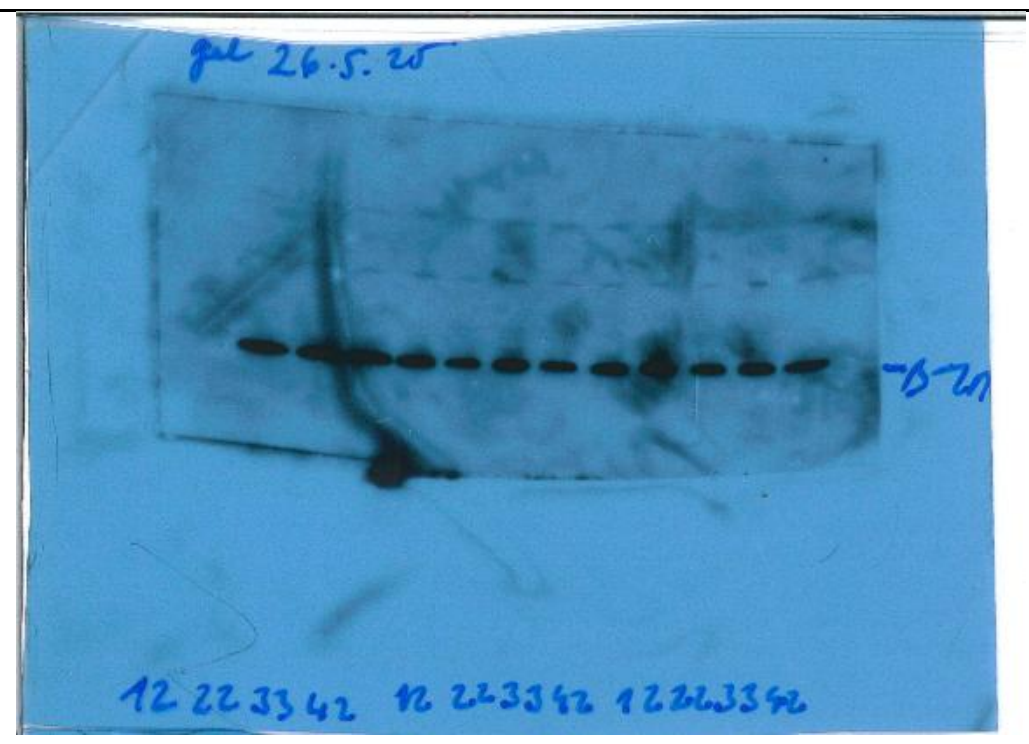
13,23,34,43-3x	
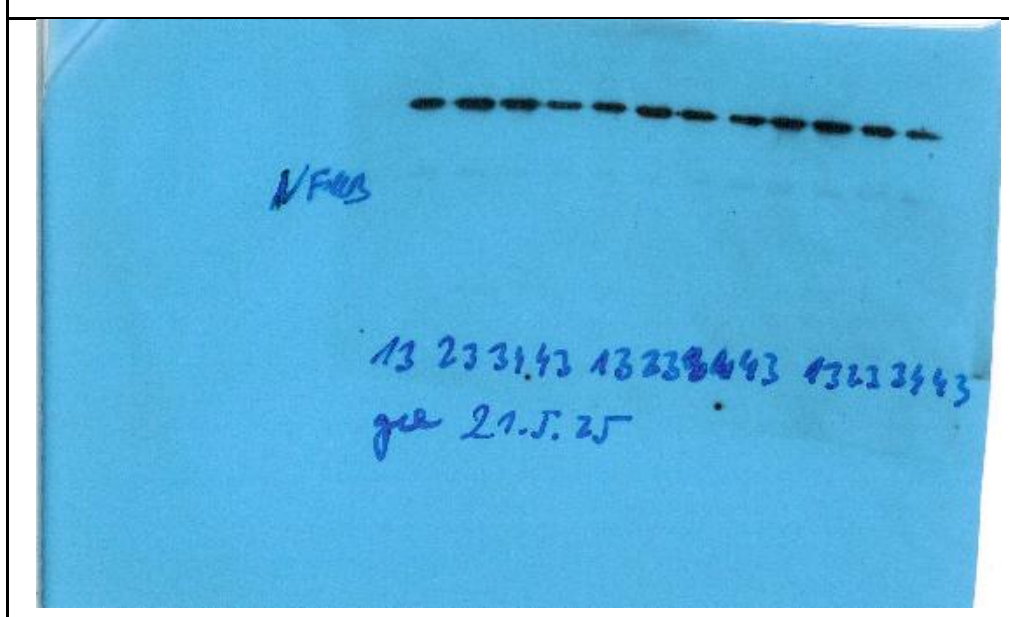	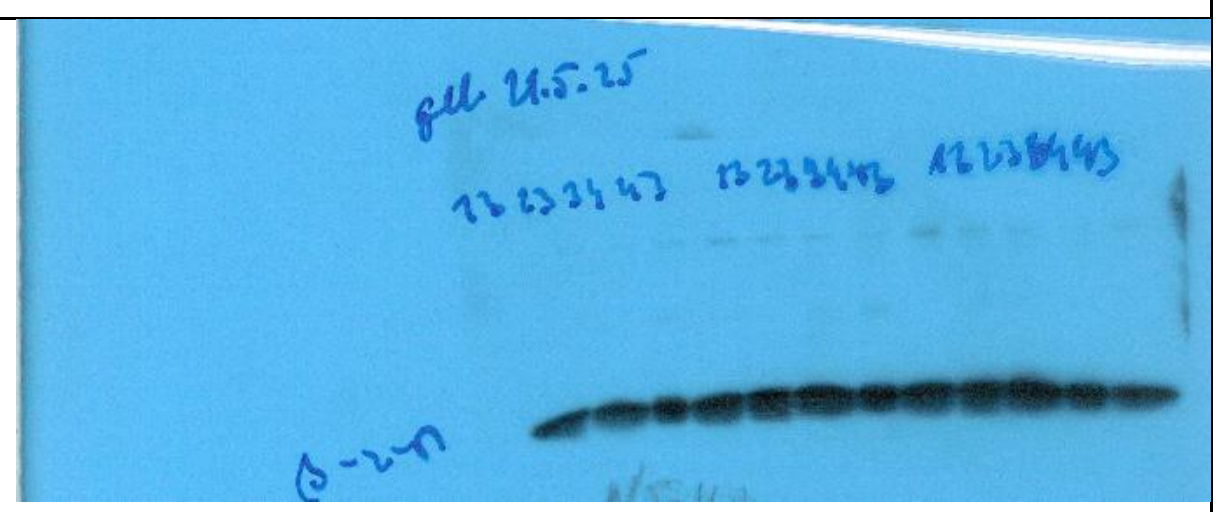
14,24,35,44-3x	

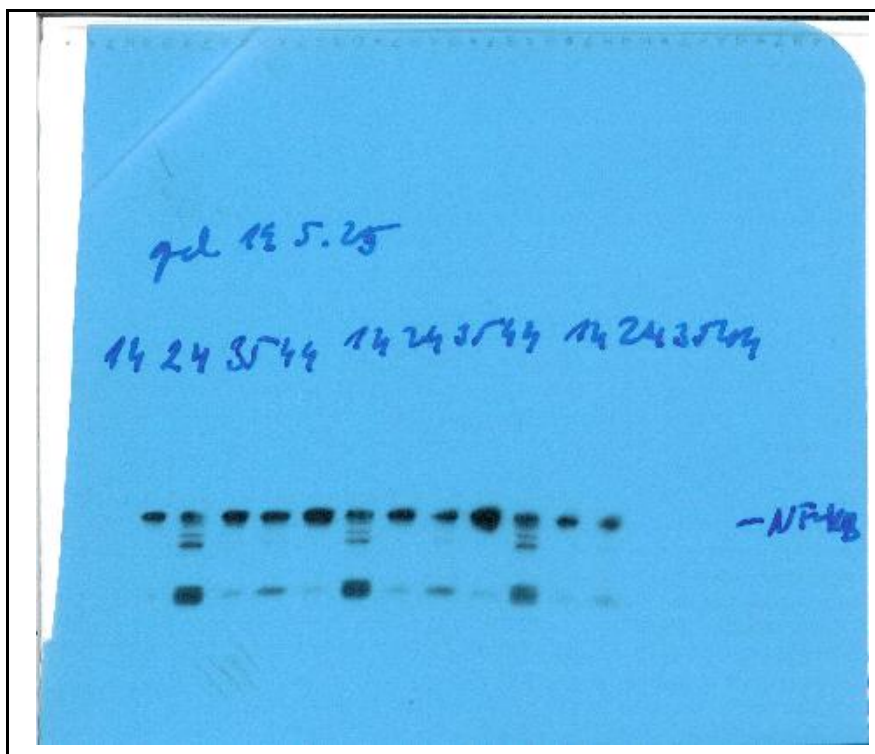	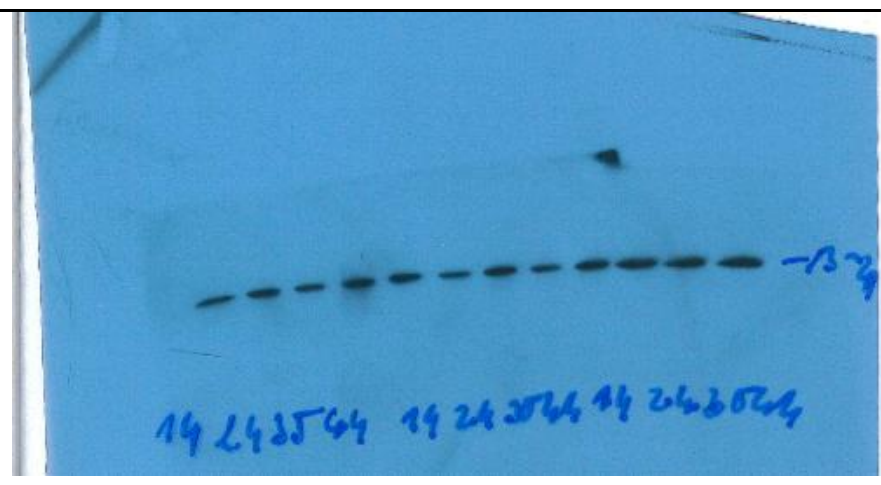
15,25,36,45-3x	
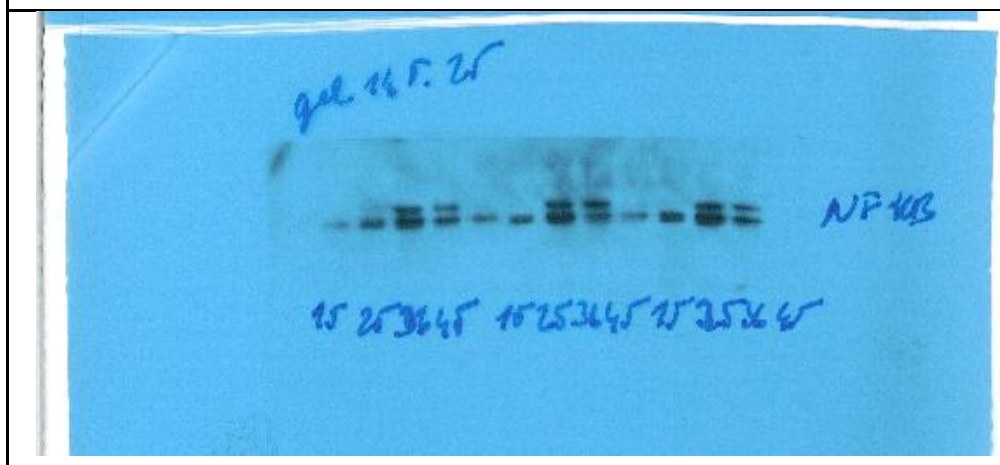	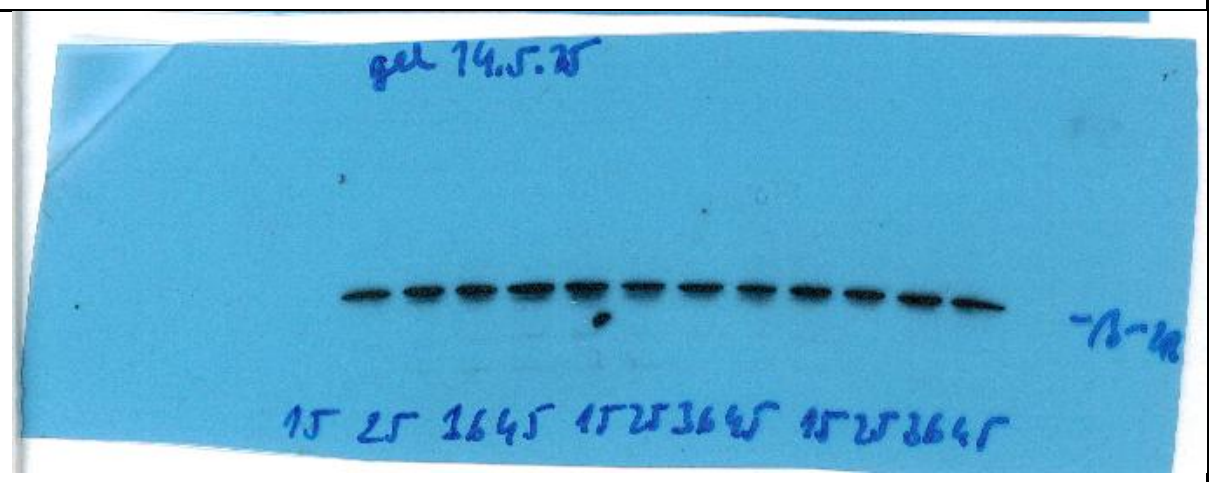
16,27,37,46-3x	
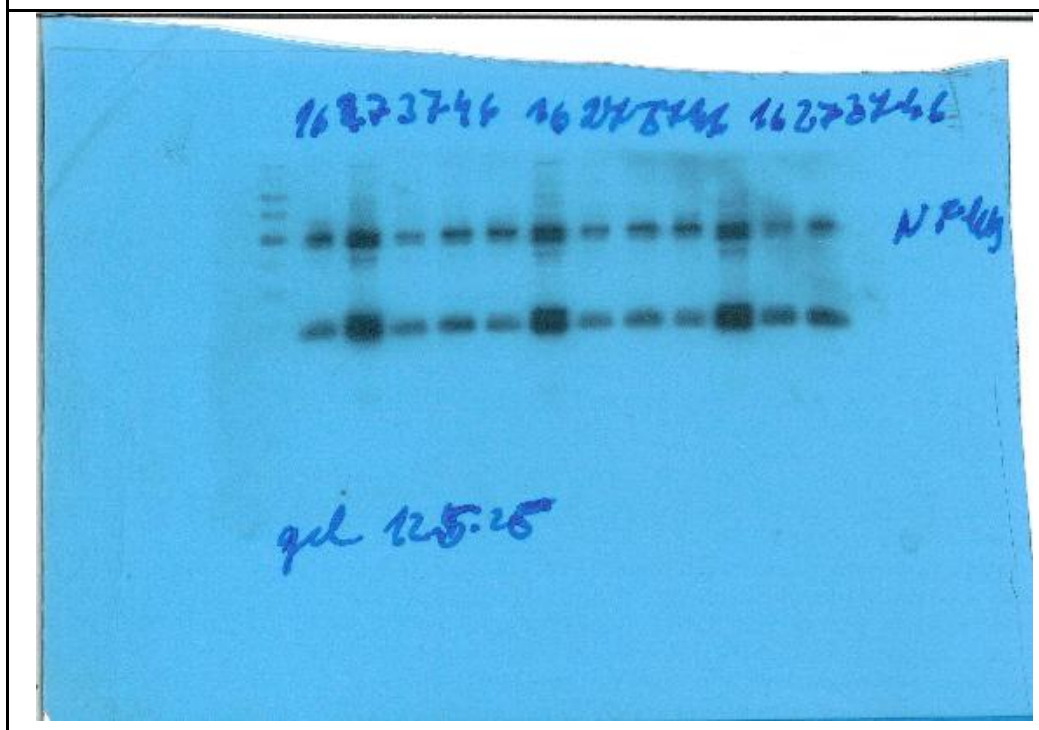	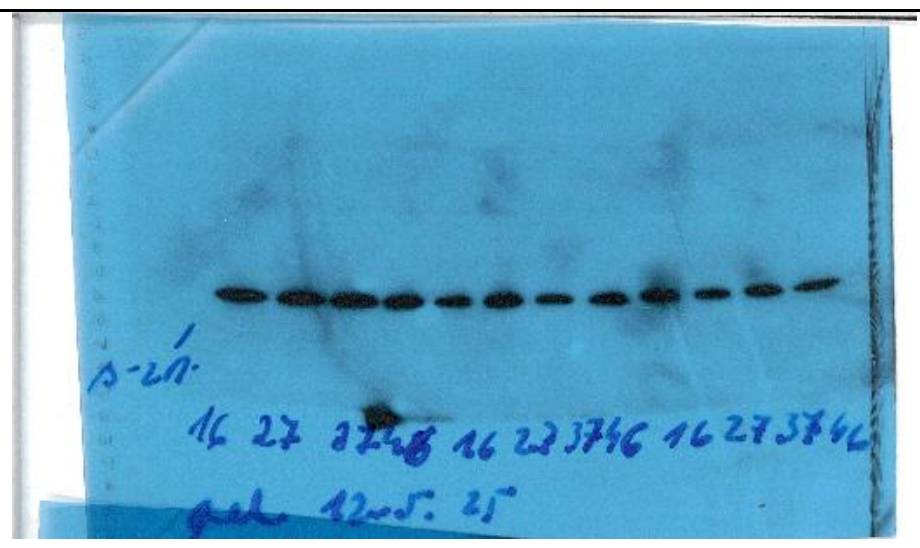
17,28,38,47-3x	
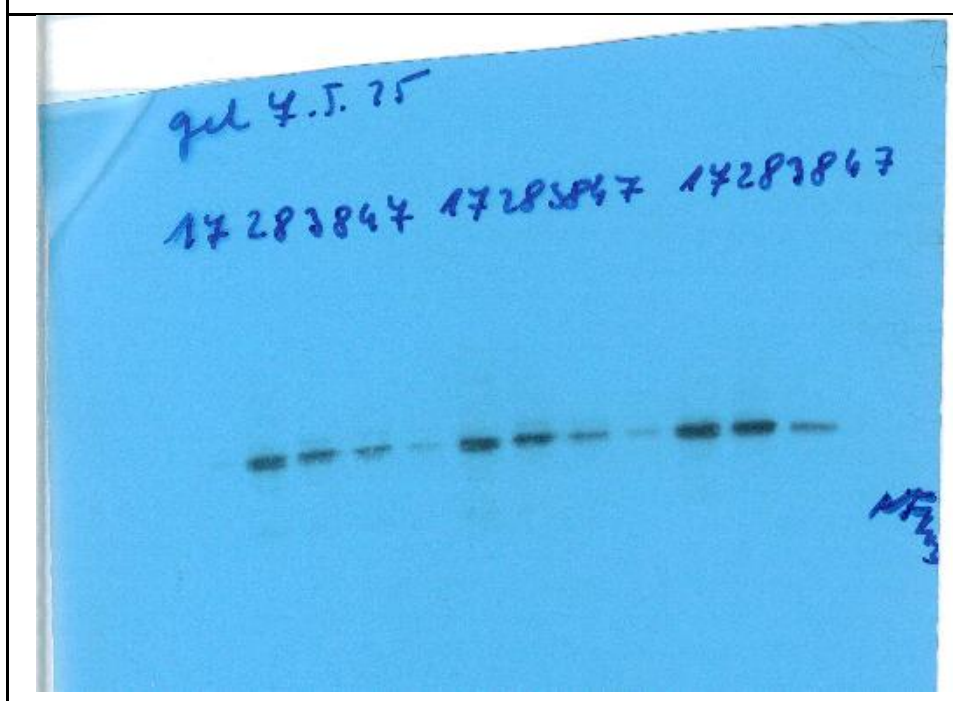	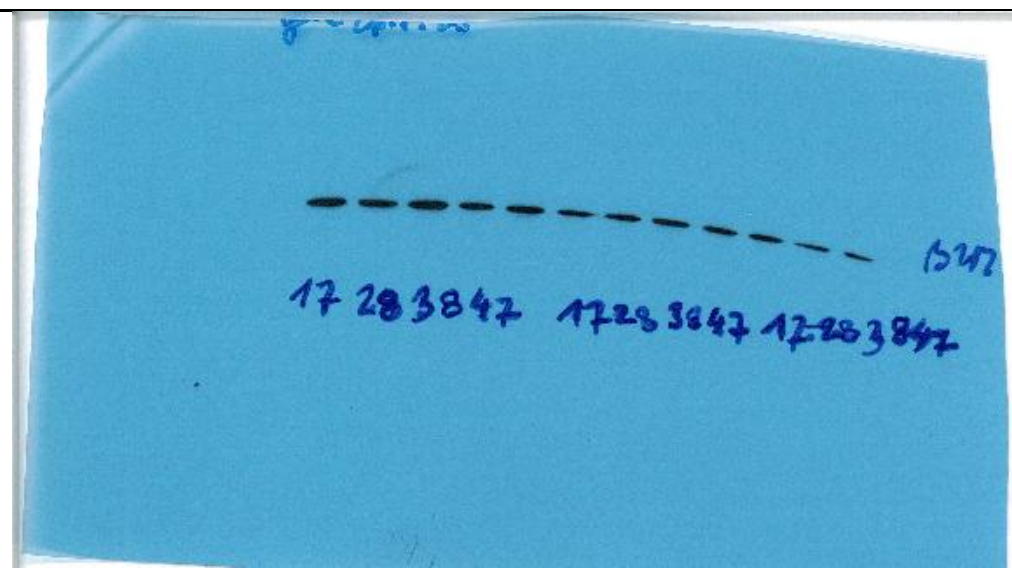
18,29,39,48-3x	

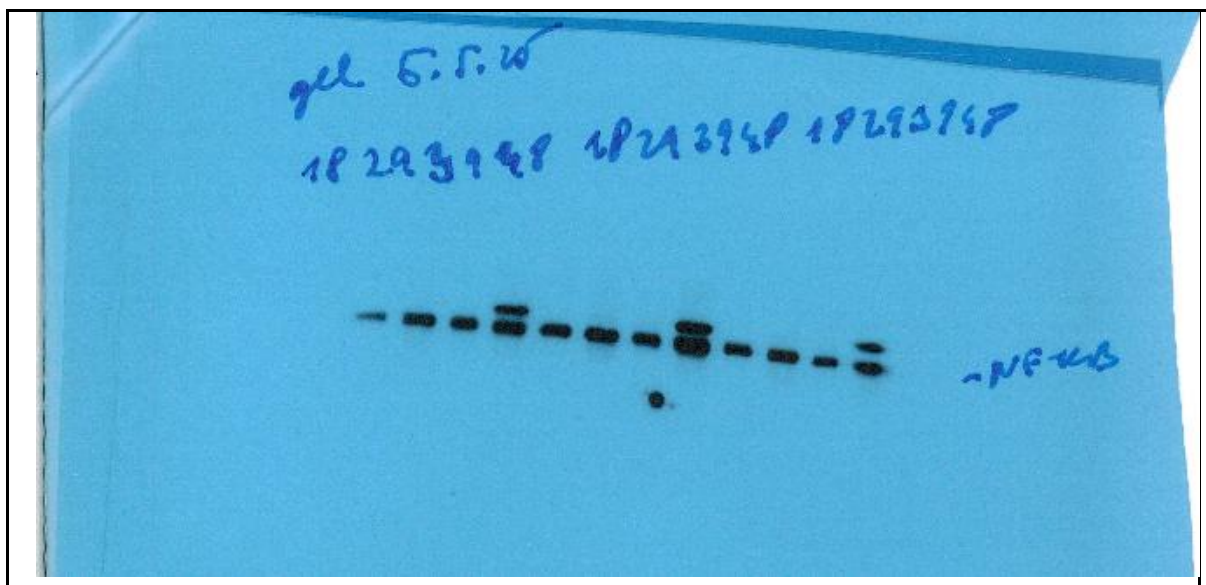	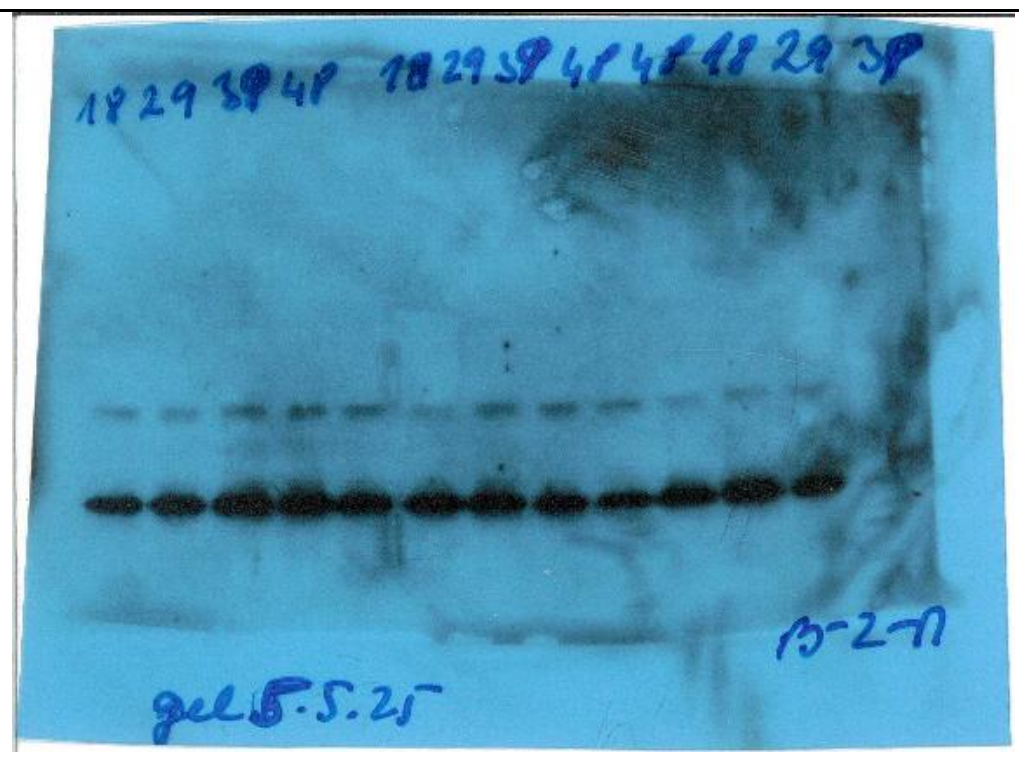
19,310,49-3x	
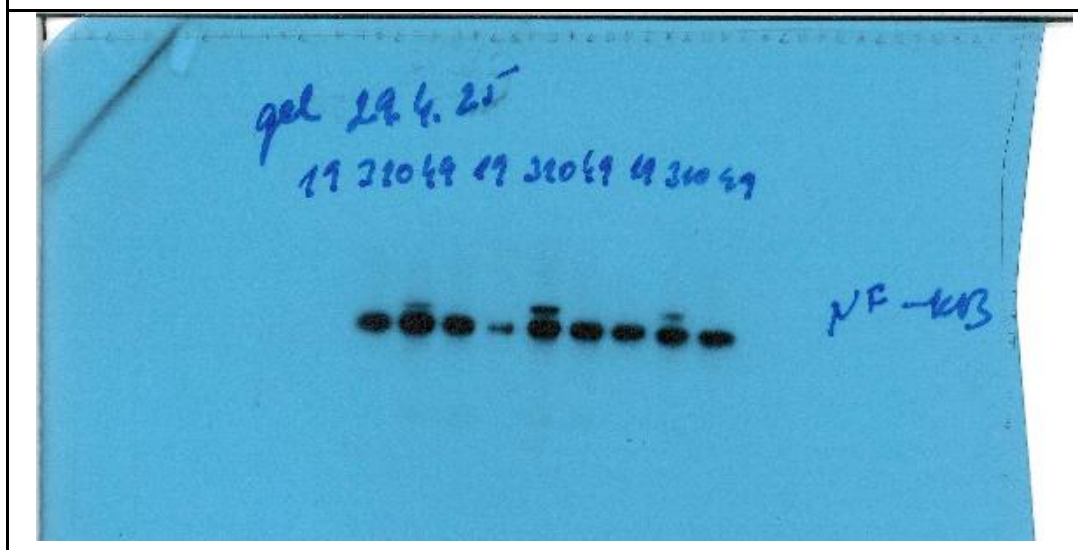	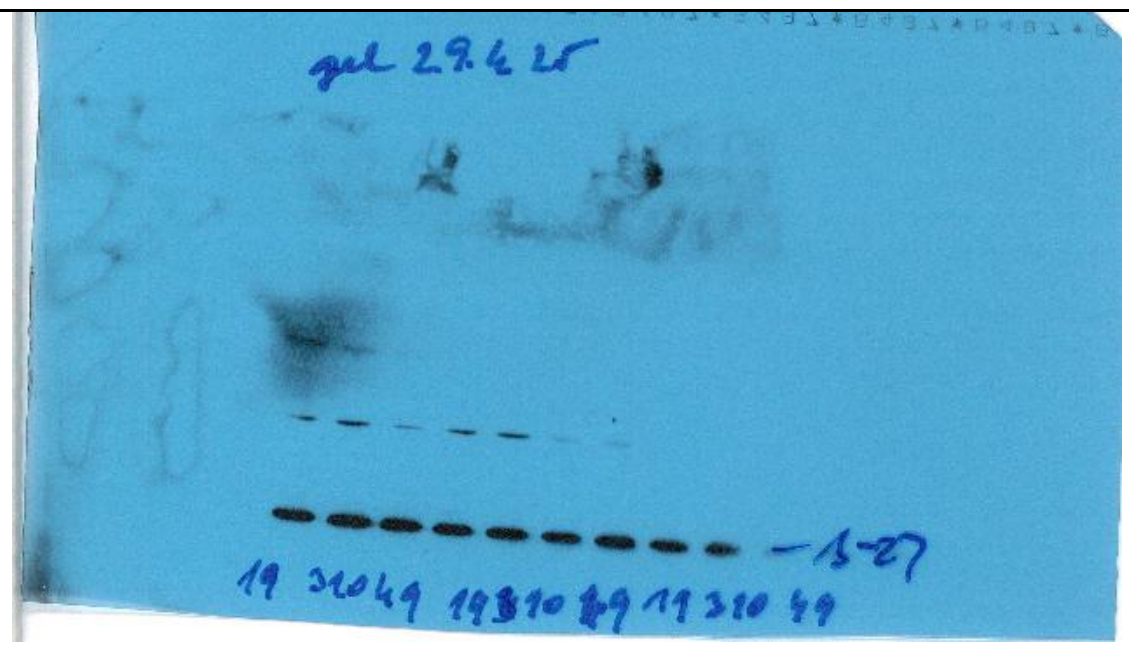

p-Nf-Kb	b-2-m
41,11,21,31-3x	
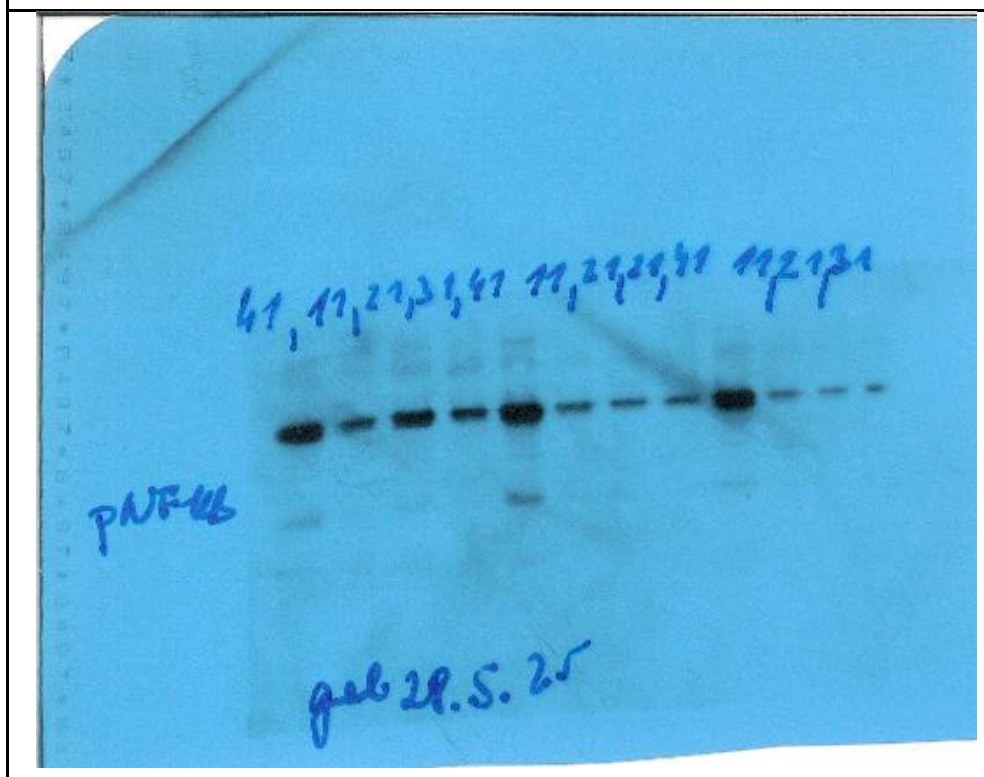	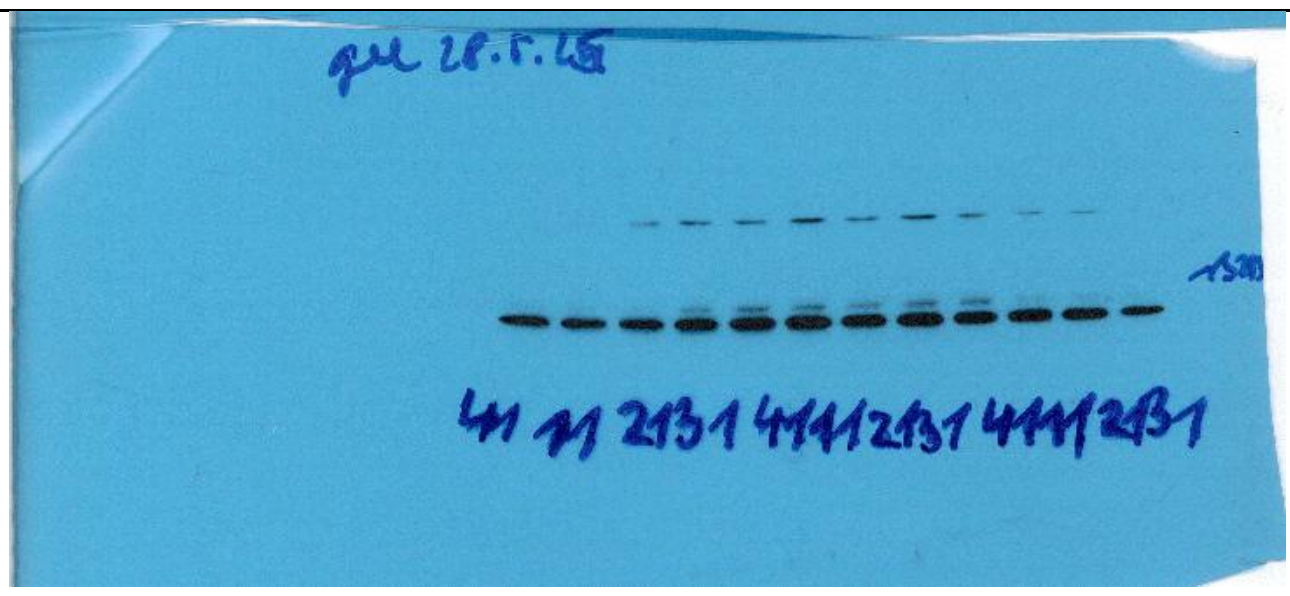
12,22,33,42-3x	

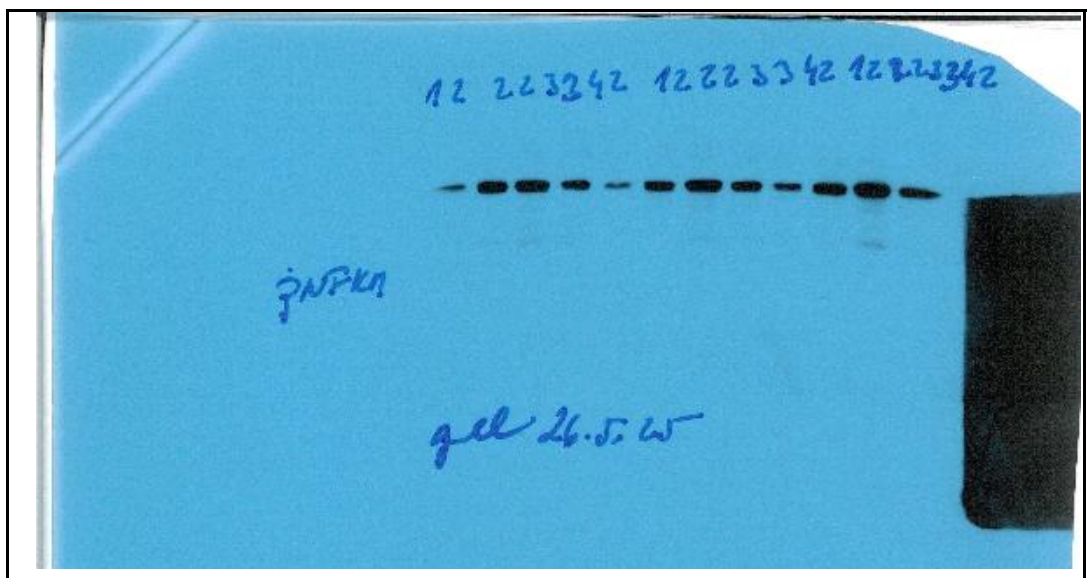	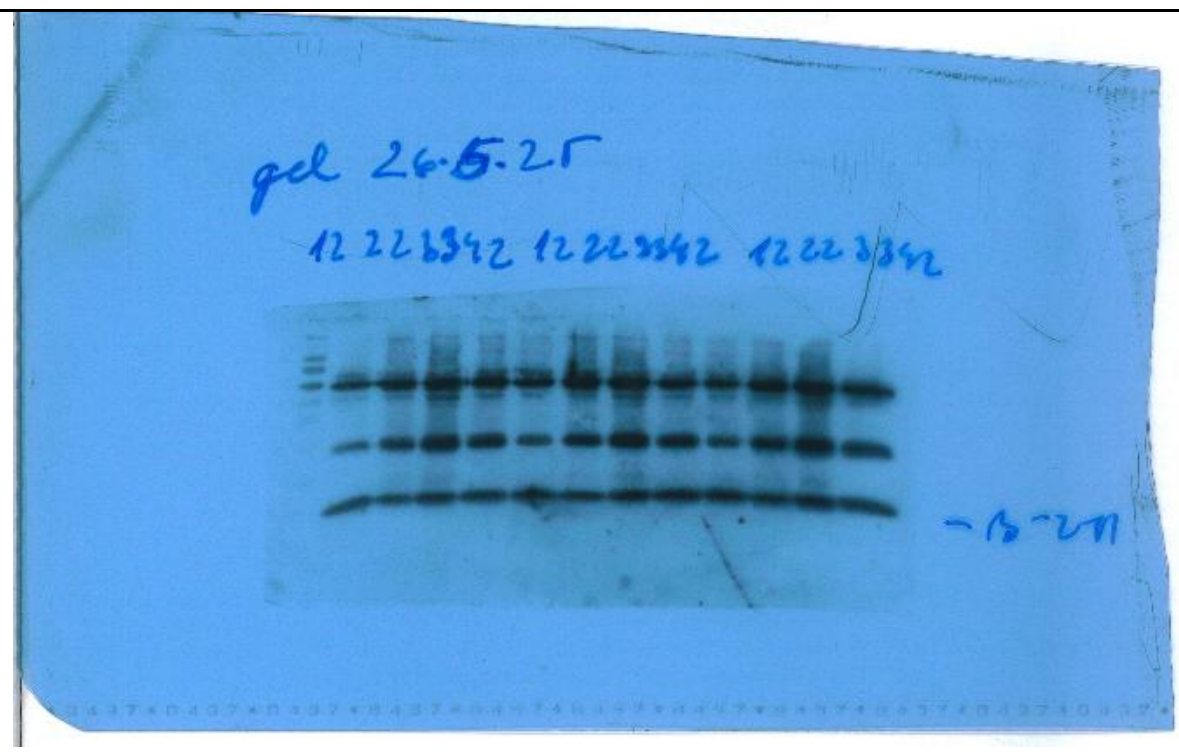
13,23,34,43-3x	
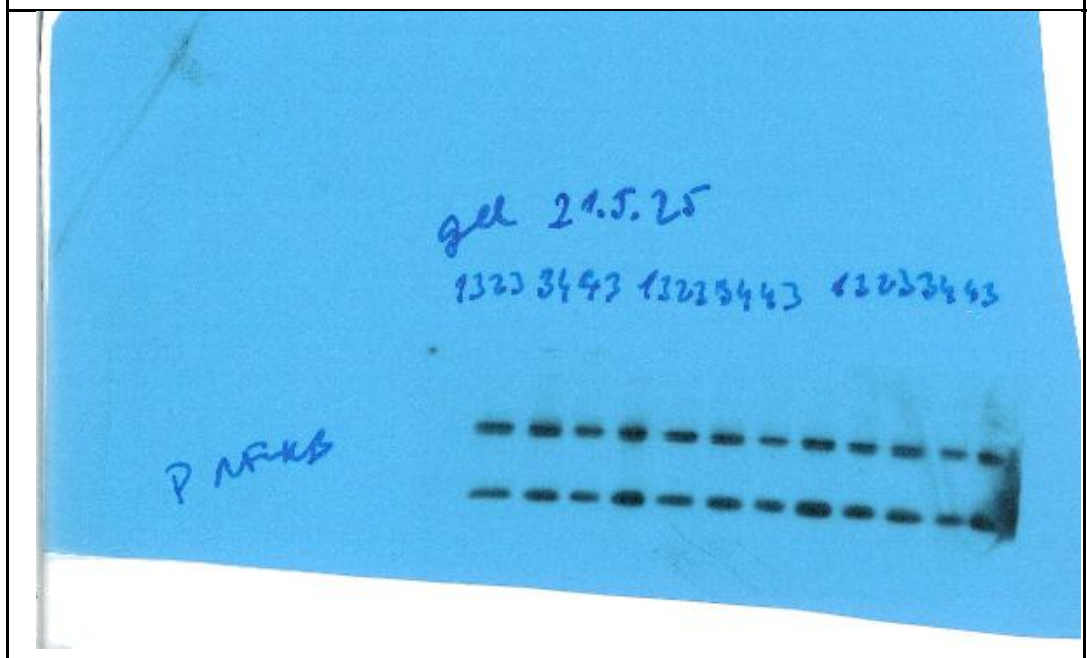	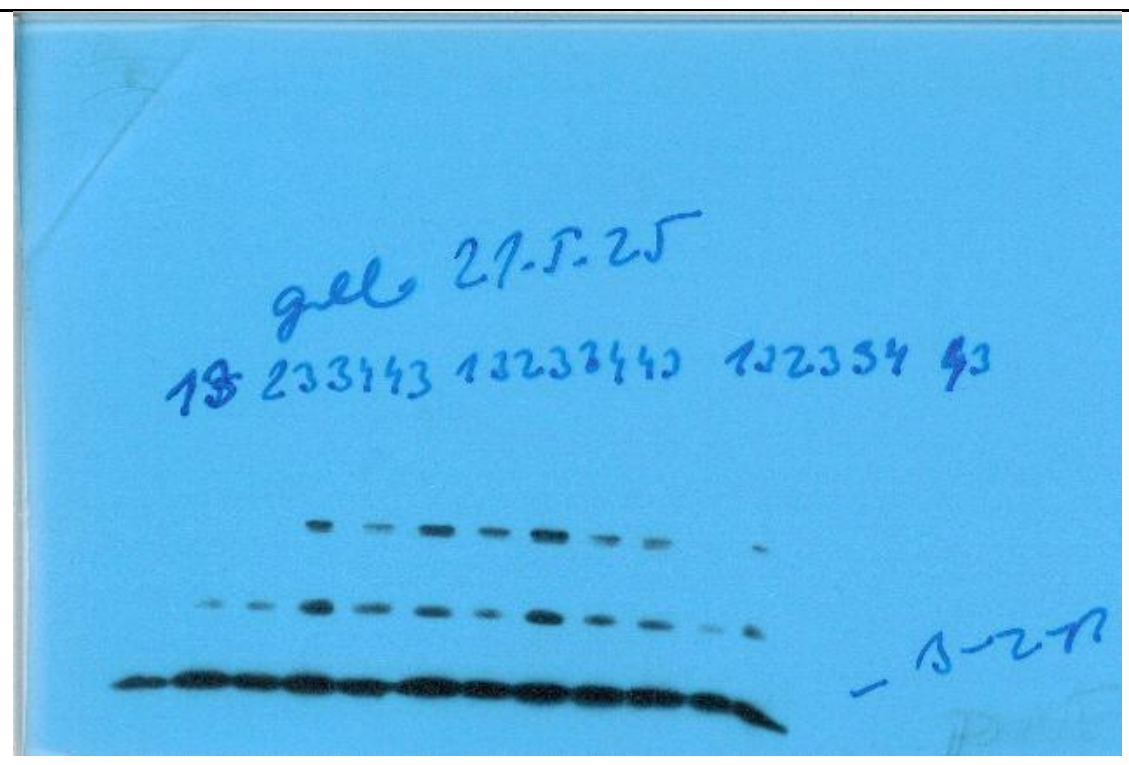
14,24,35,44-3x	
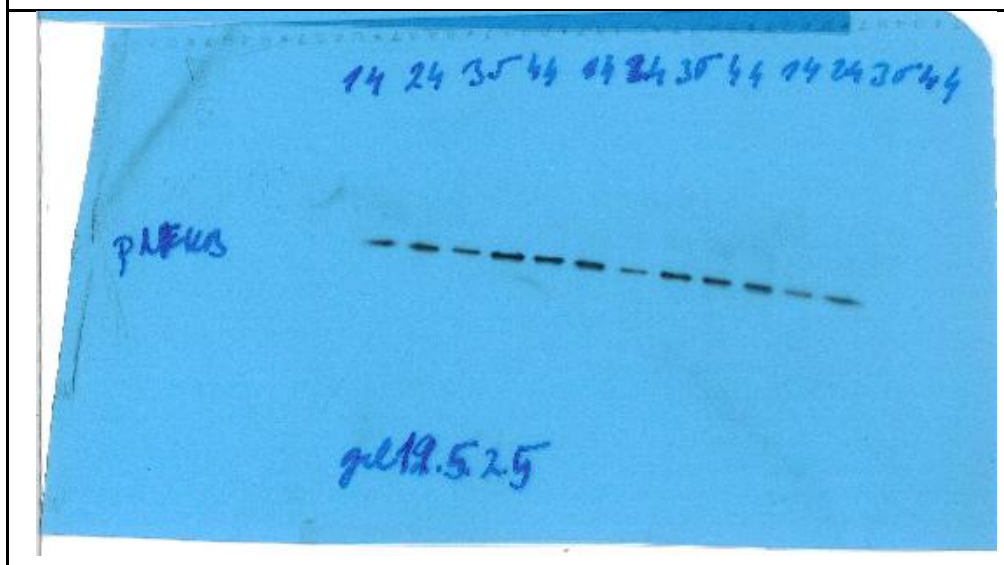	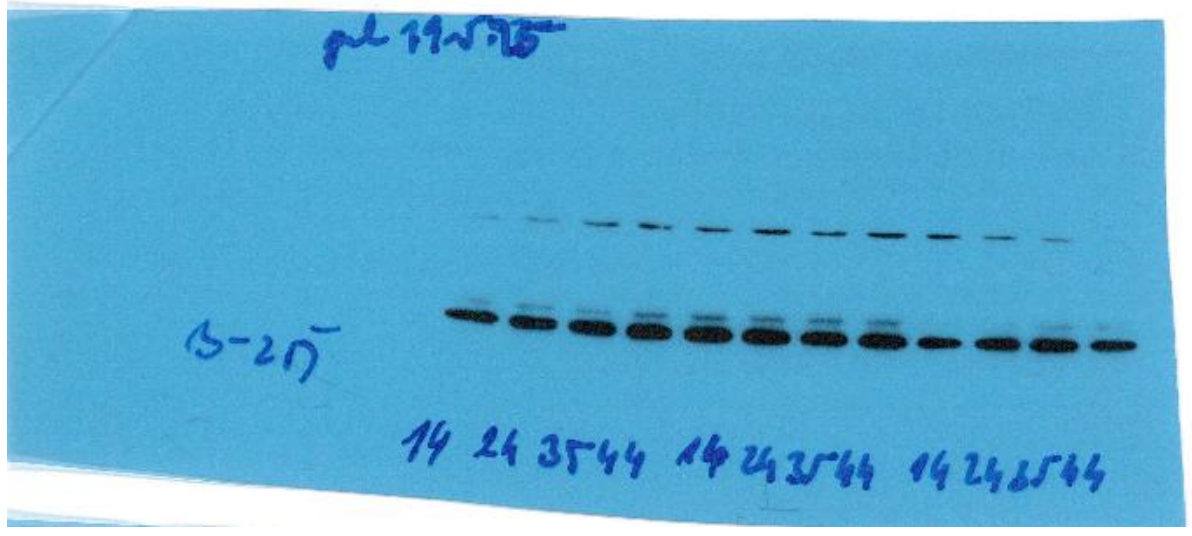
15,25,36,45-3x	
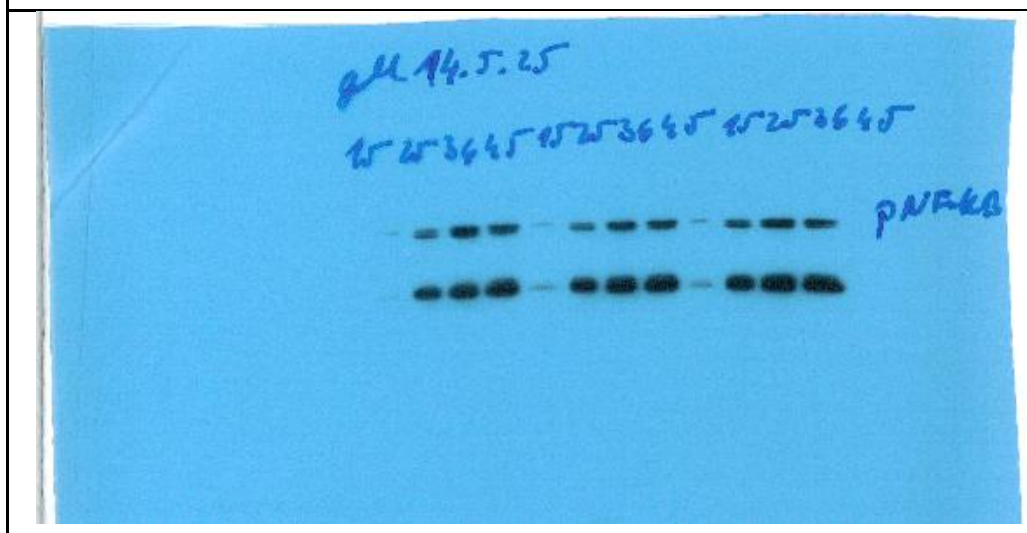	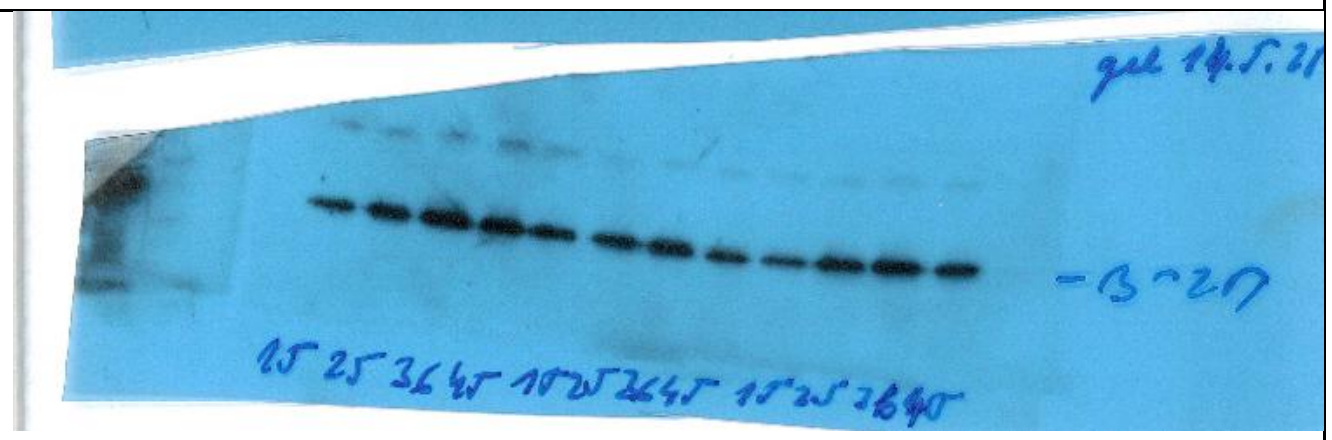
16,27,37,46-3x	

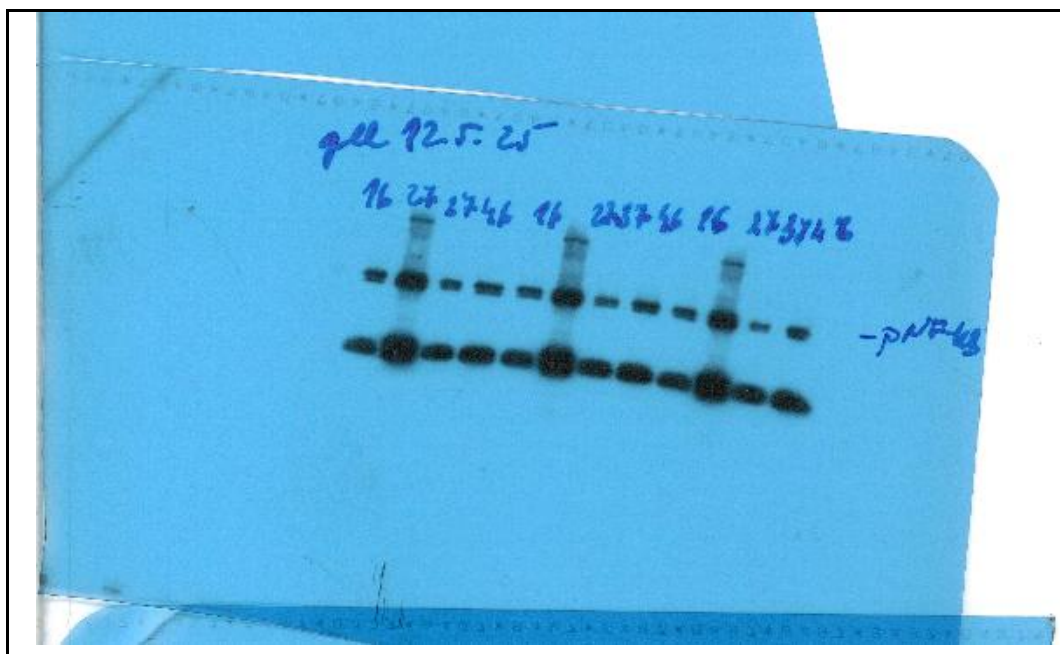	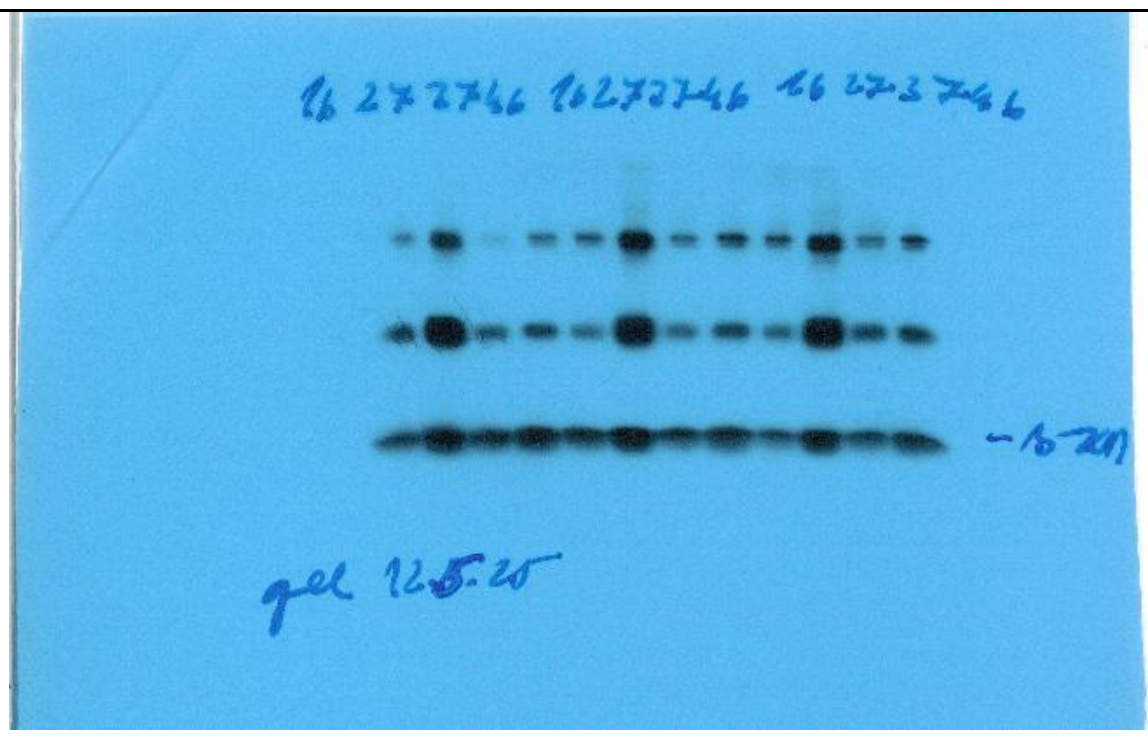
17,28,38,47-3x	
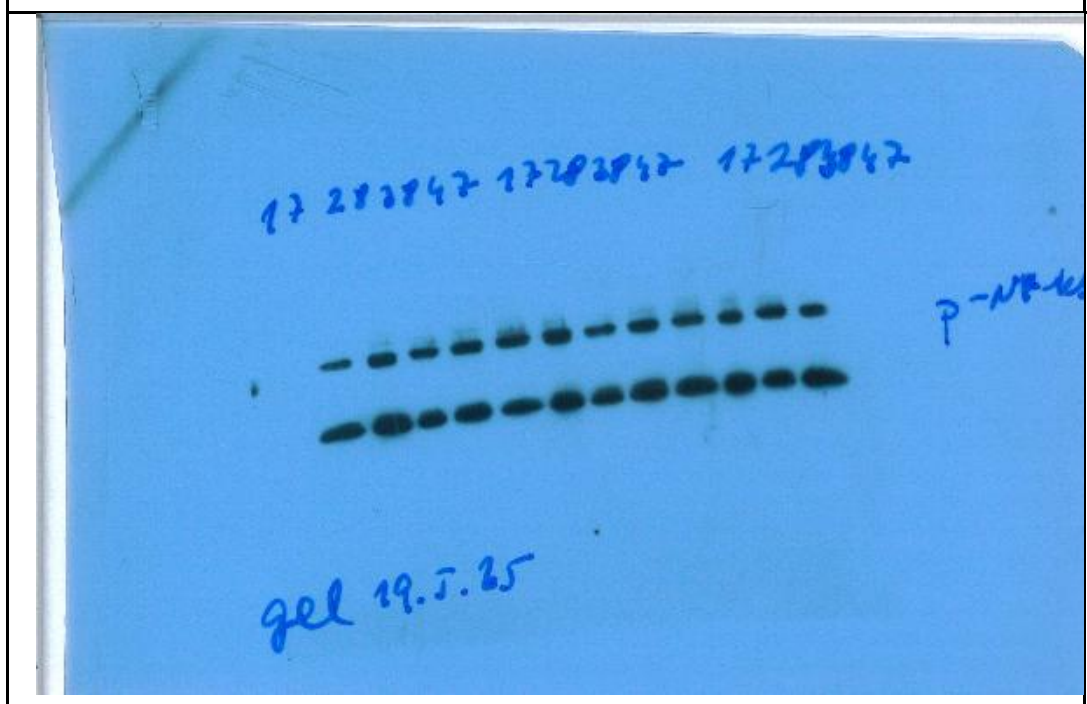	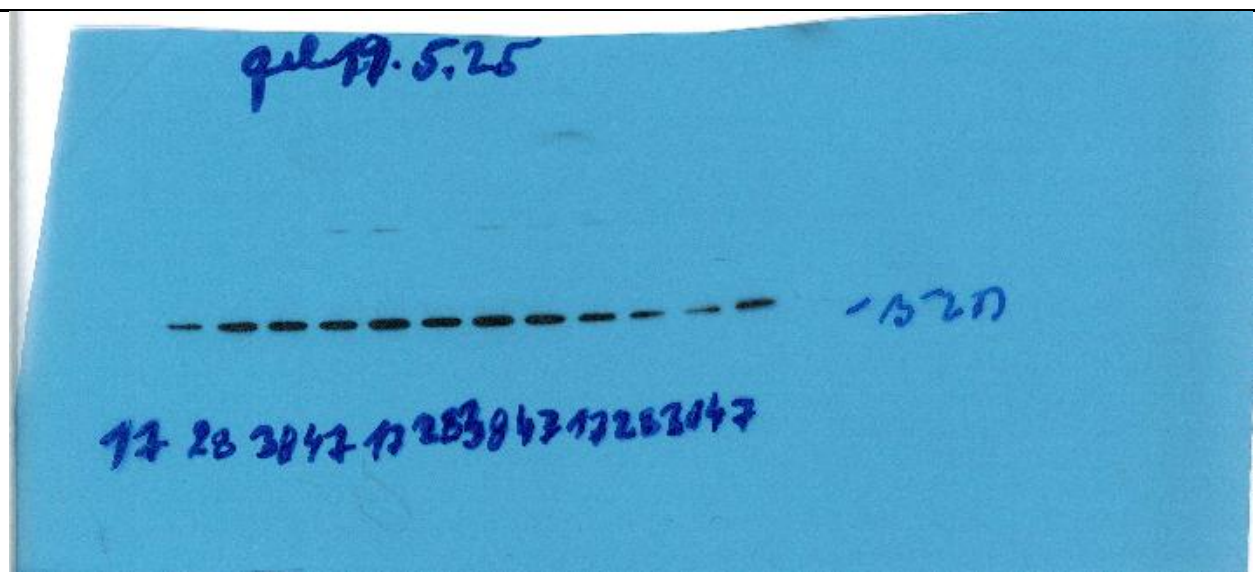
18,29,39,48-3x	
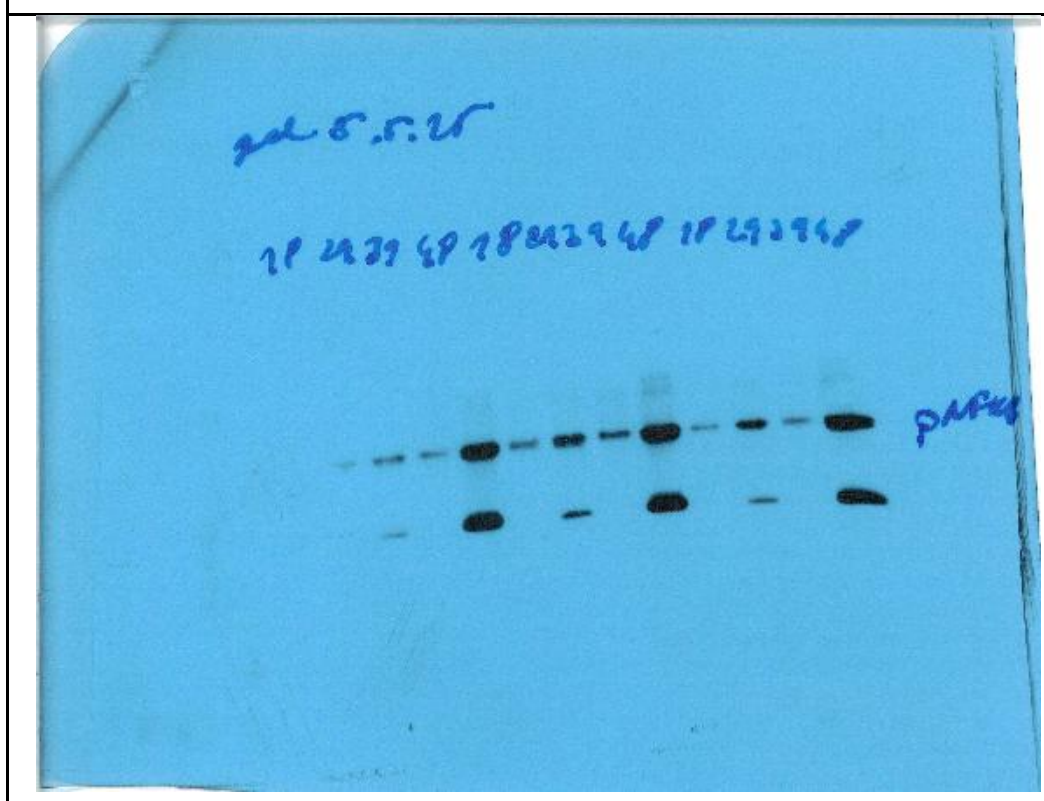	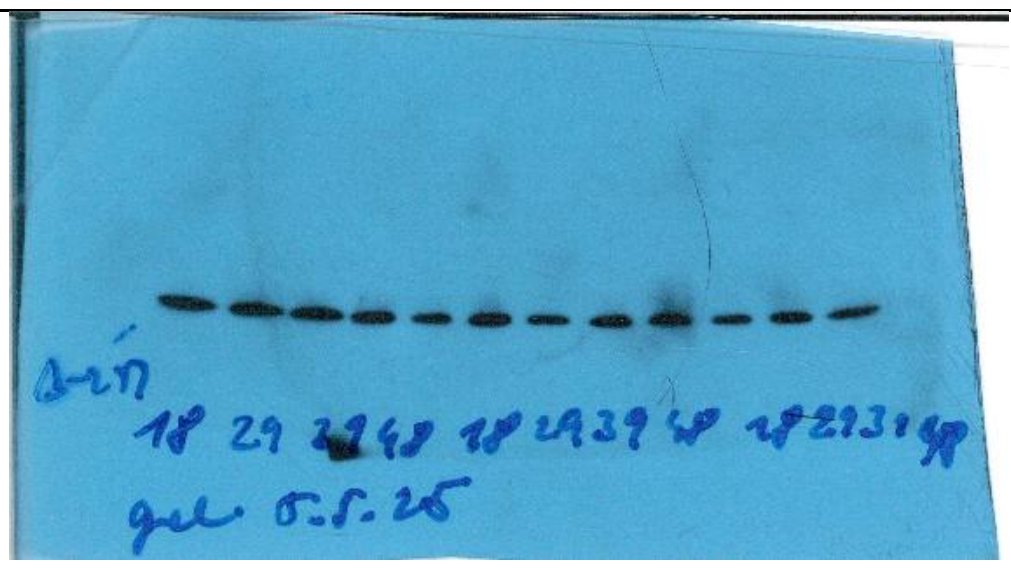
19,310,49-3x	
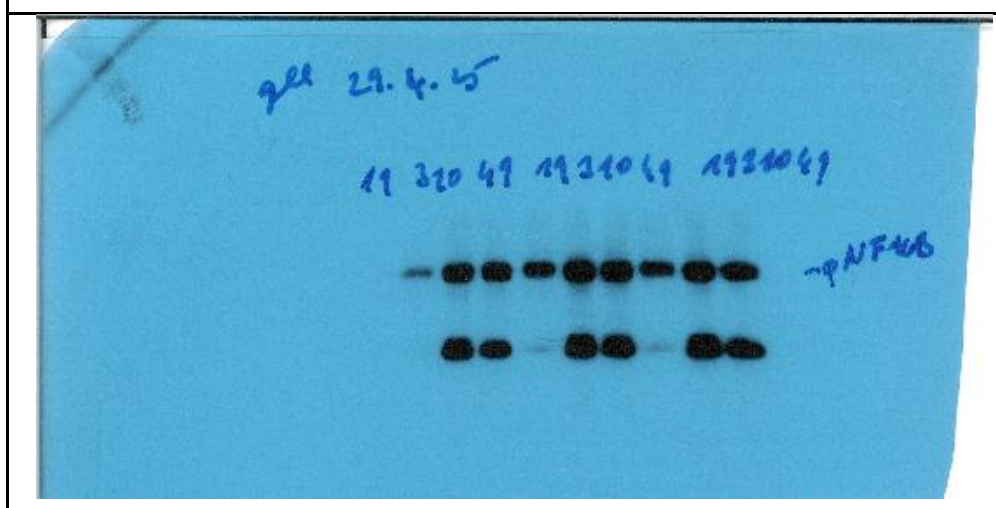	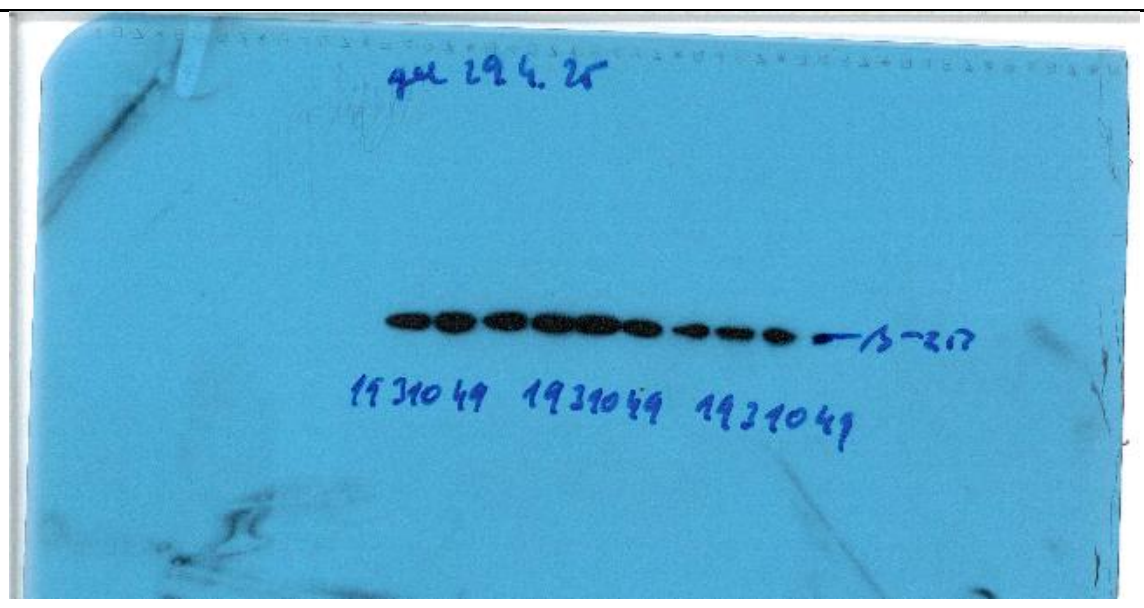

NLRP-3	b-2-m
41,11,21,31 -3x	
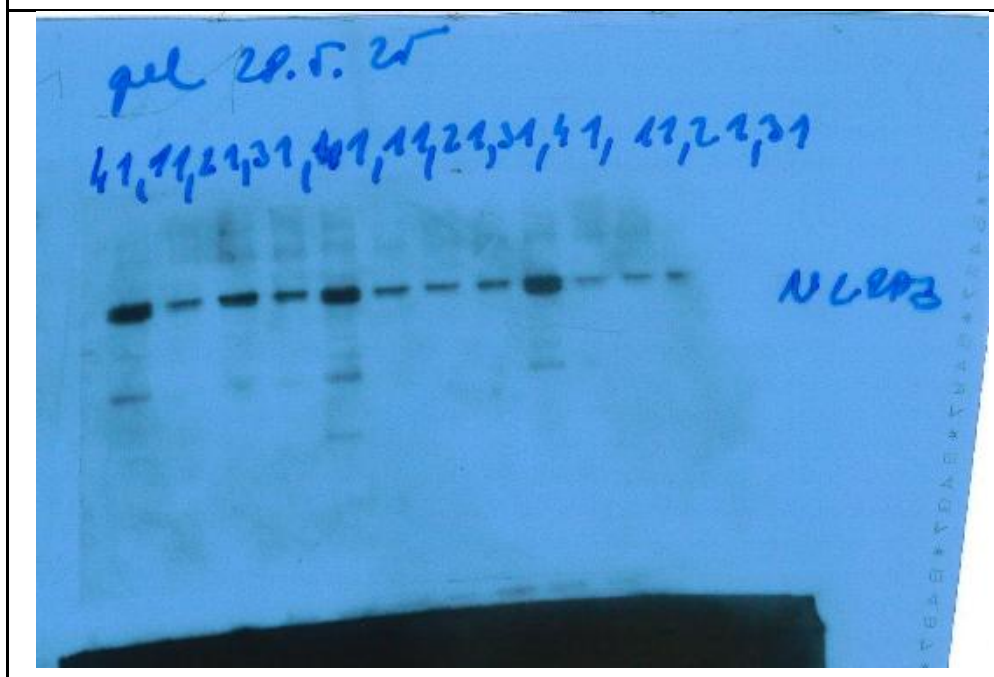	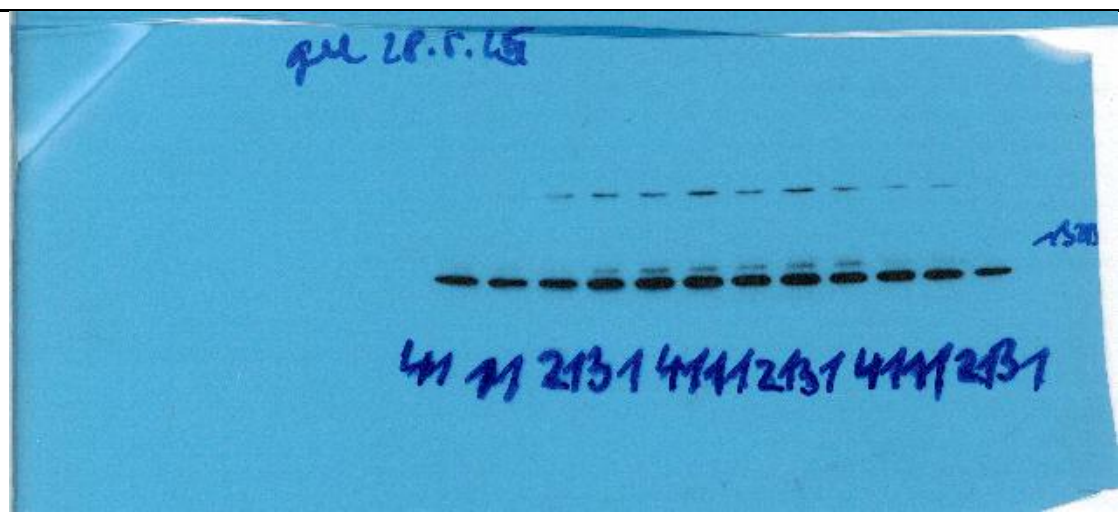
12,22,32,42 – 3x	
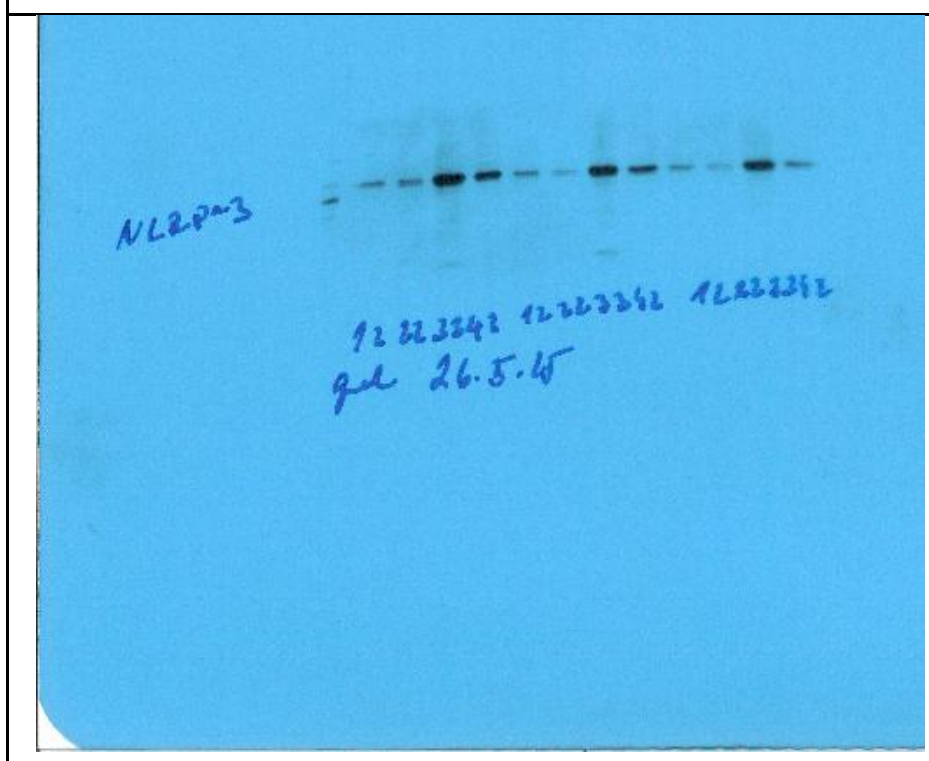	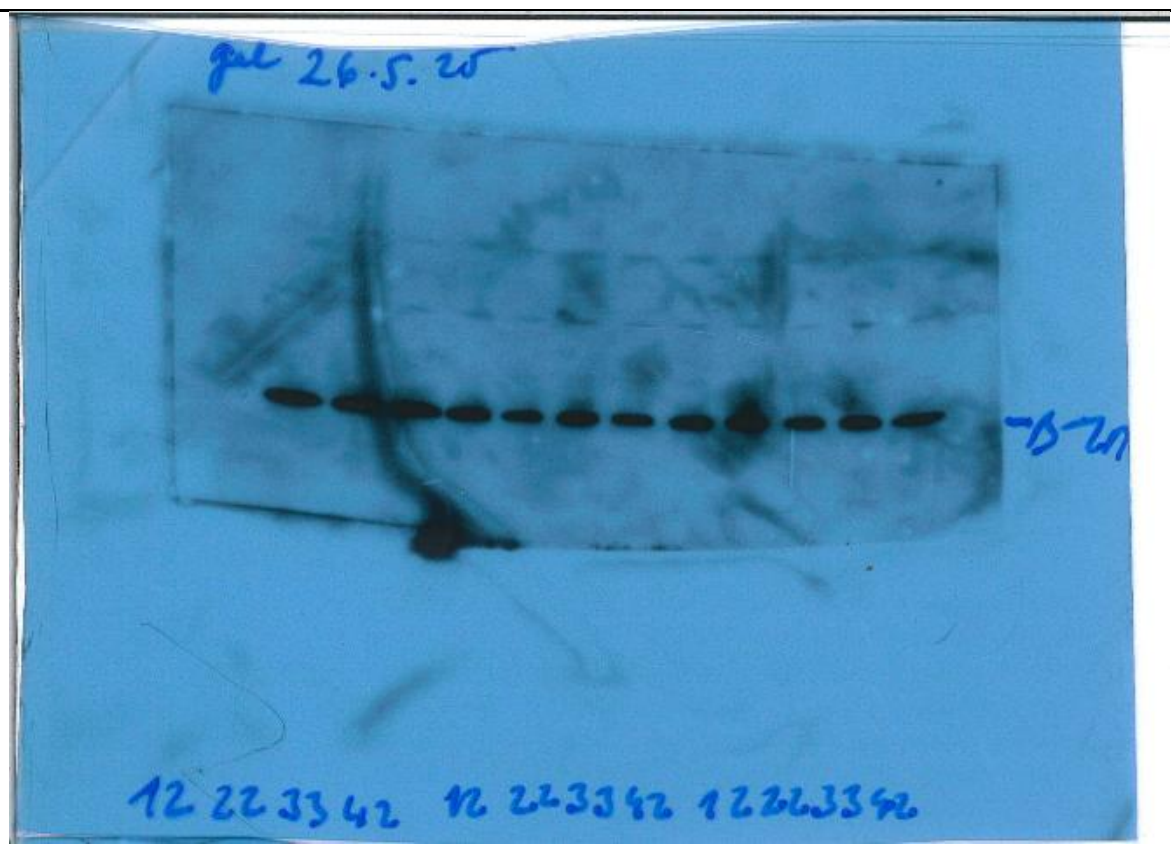
13,23,34,43 – 3x	
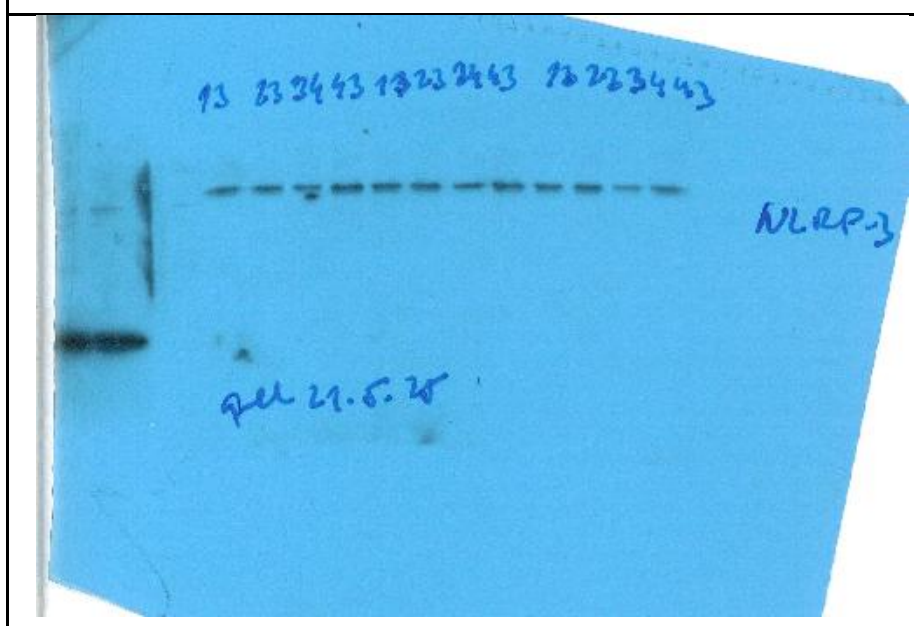	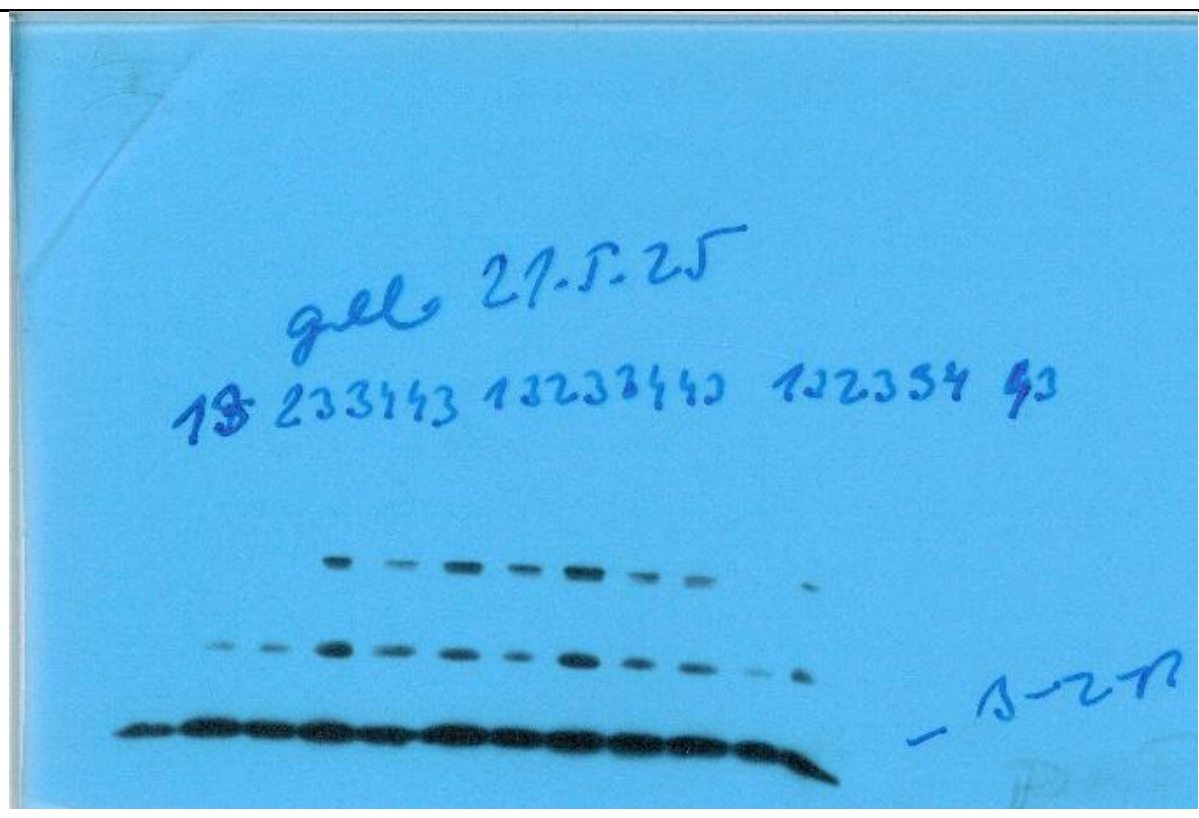
14,24,35,44 – 3x	

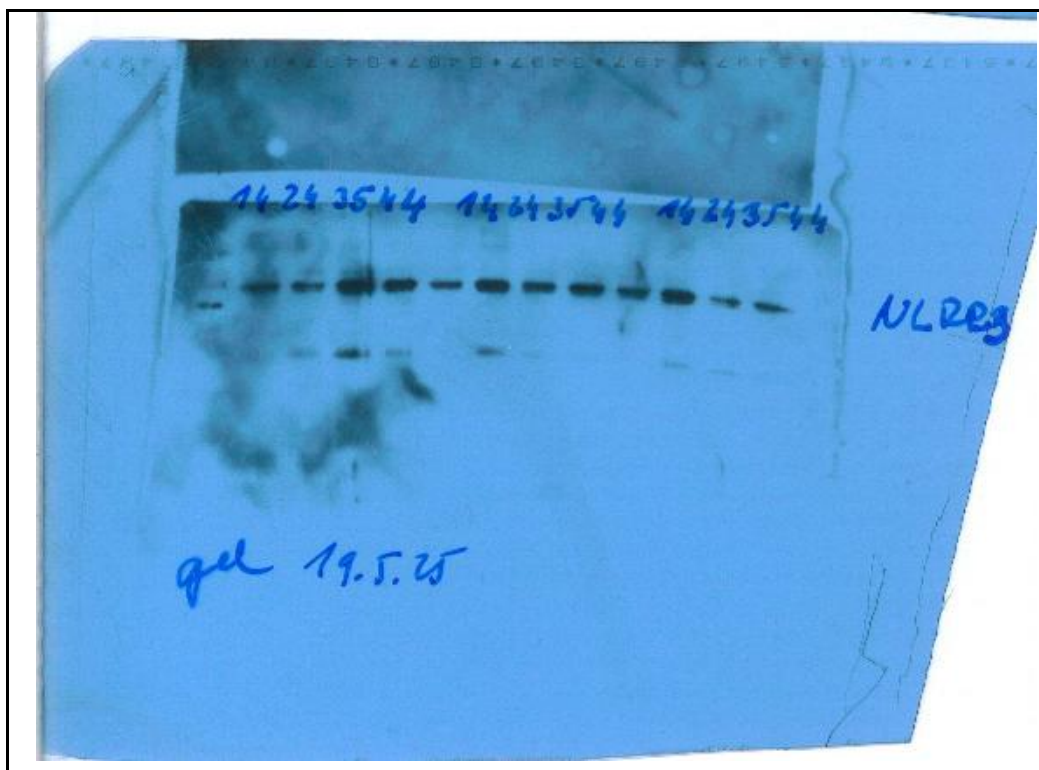	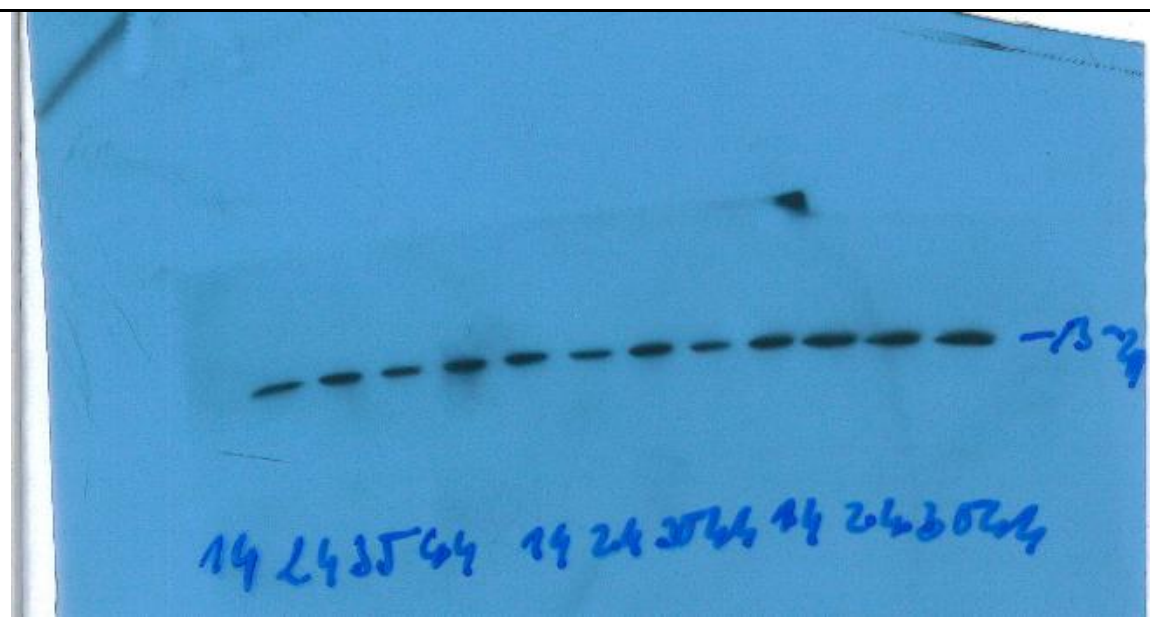
12,25,36,45 – 3x	
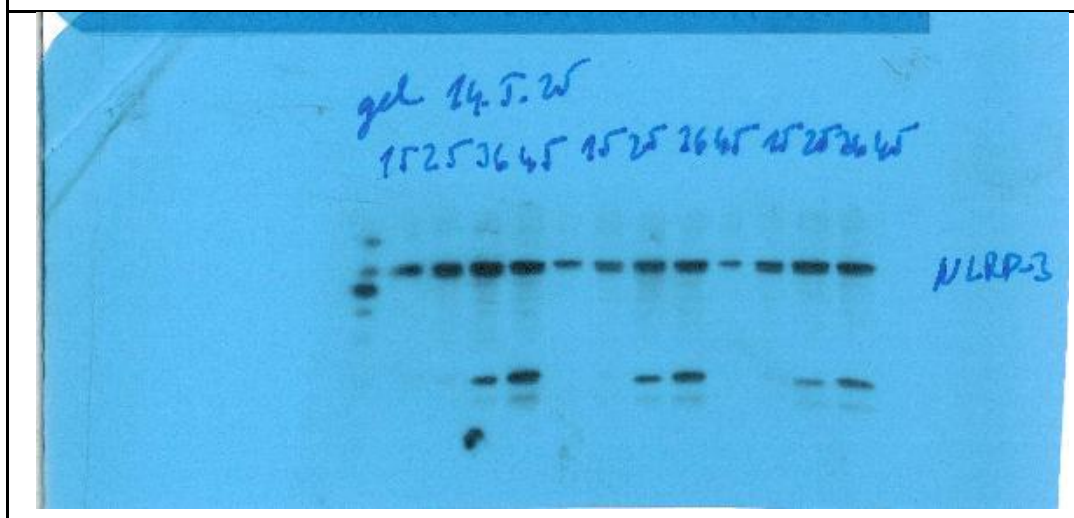	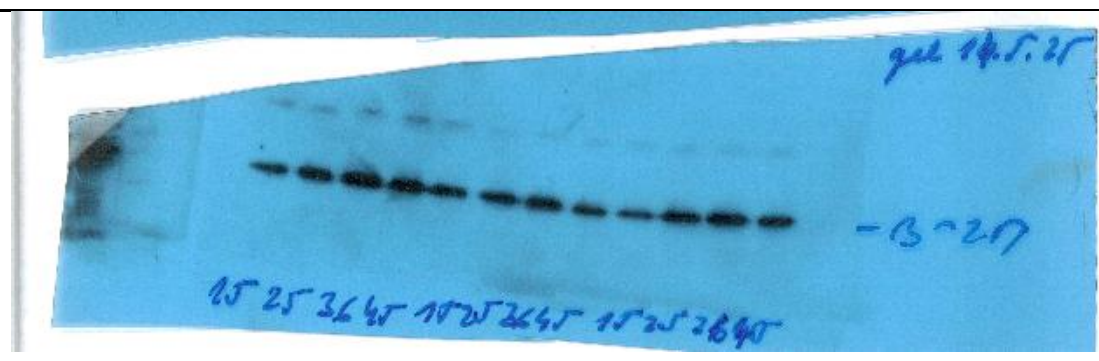
16,27,37,46 – 3x	
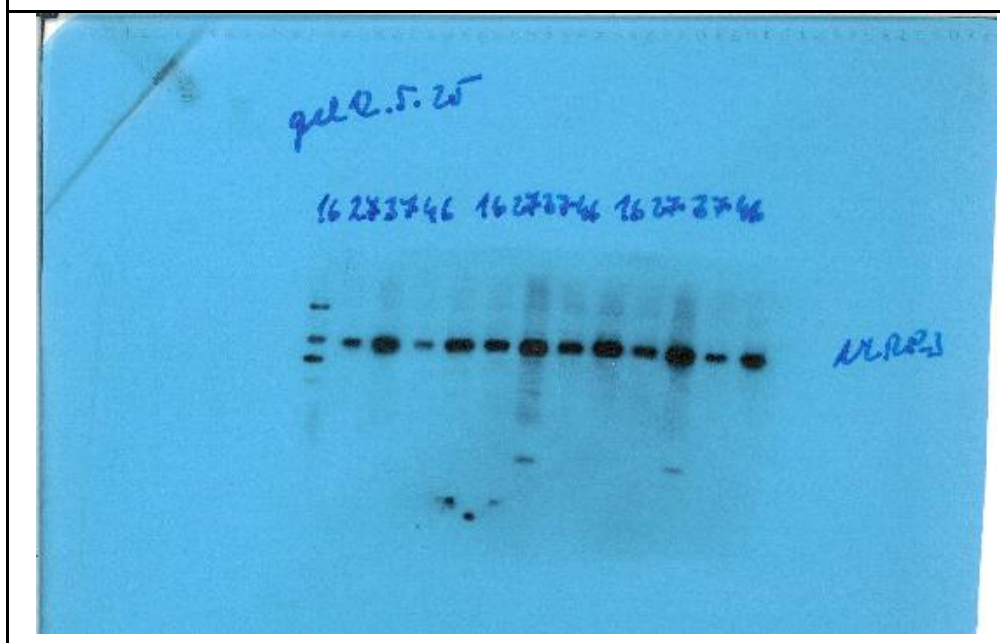	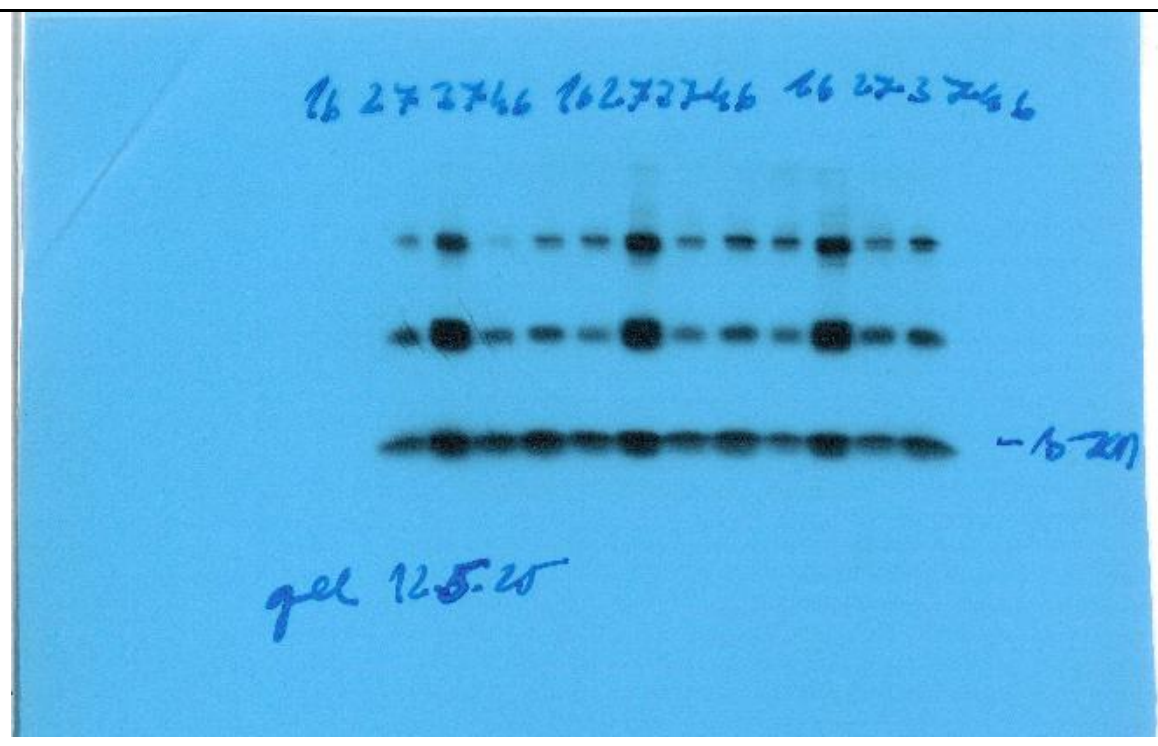
28,38,47,17 – 3x	
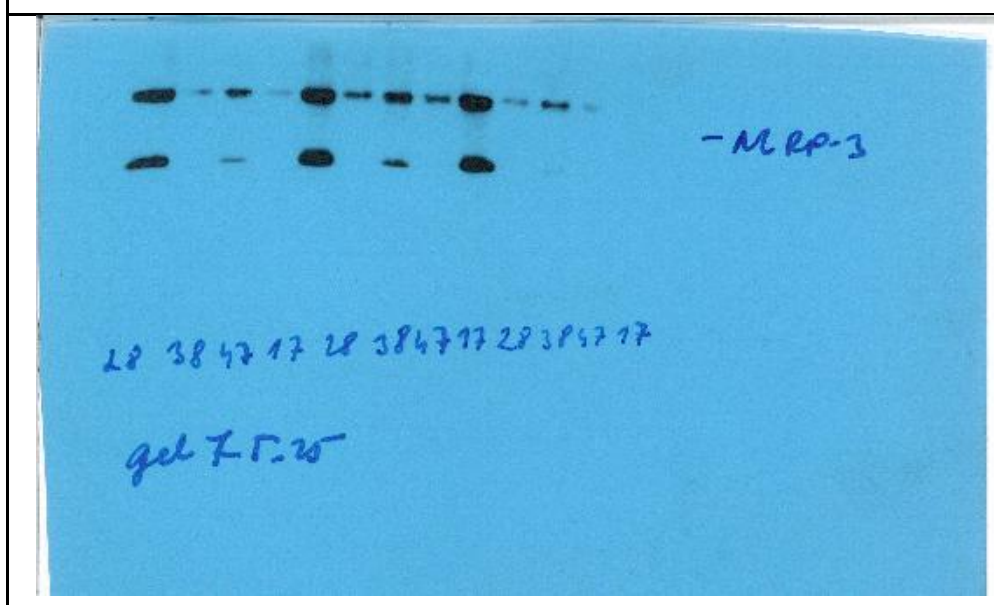	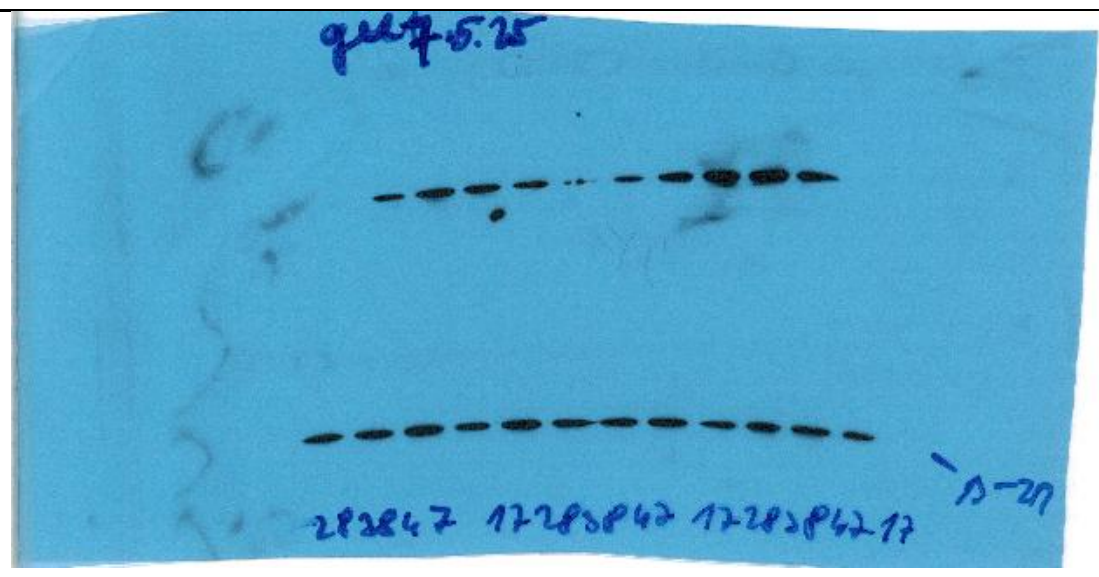
18,29,39,48 – 3x	

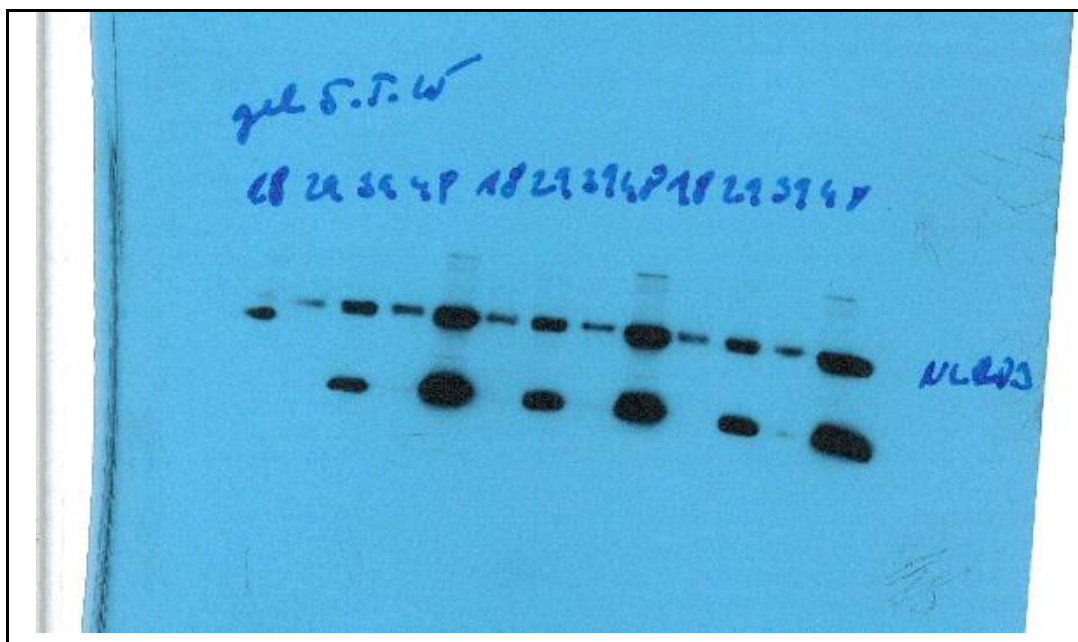	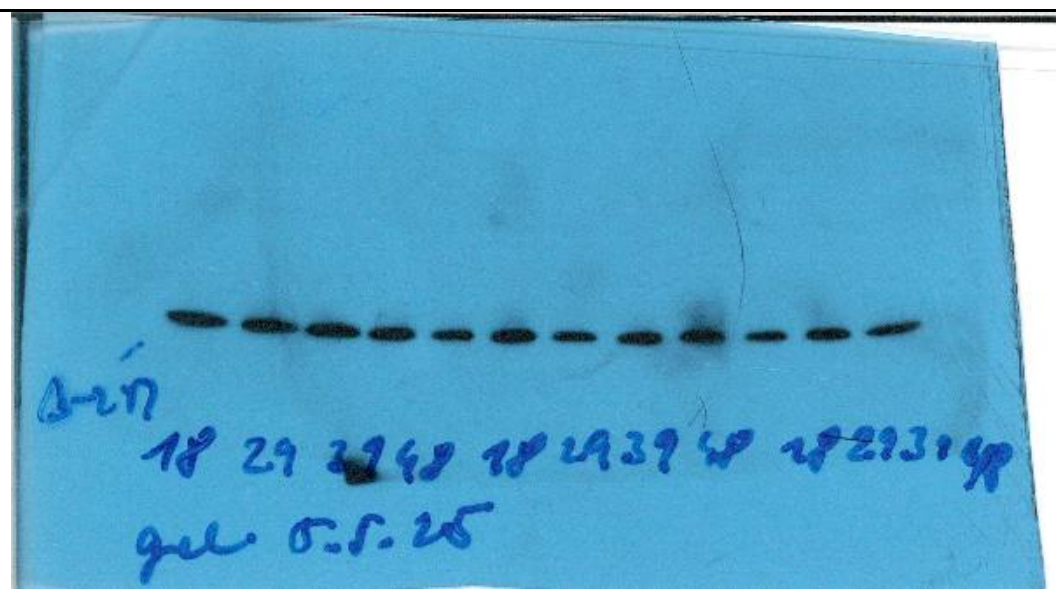
19,310,49 – 3x	
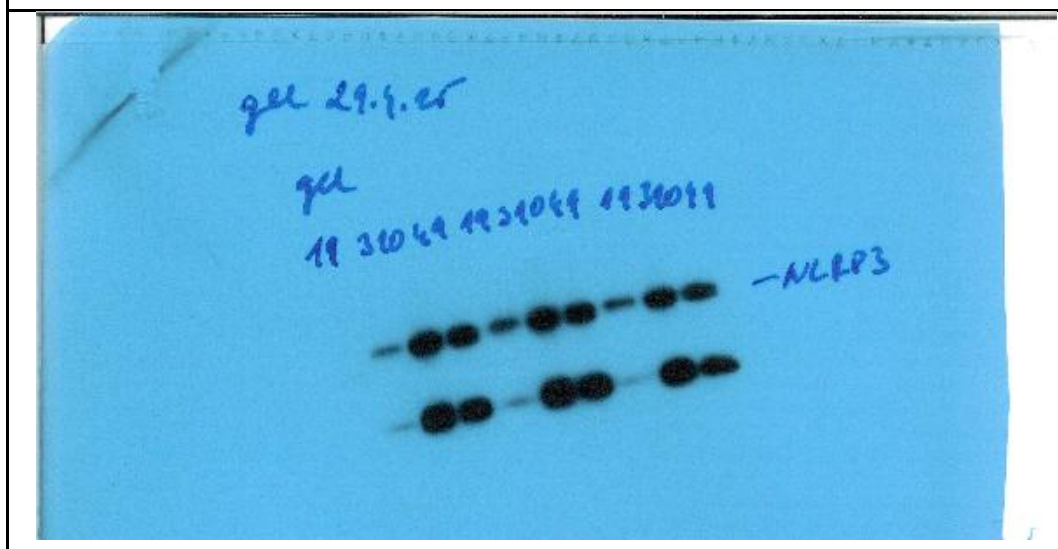	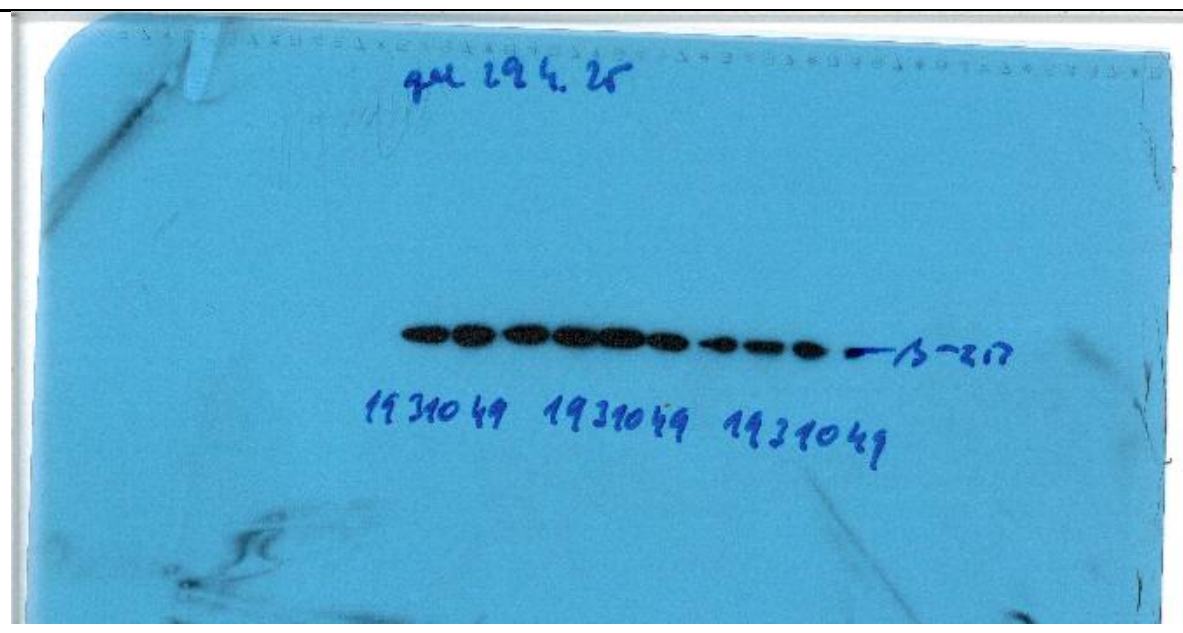

TNF	b-2-m
11,21,31,41-3x	
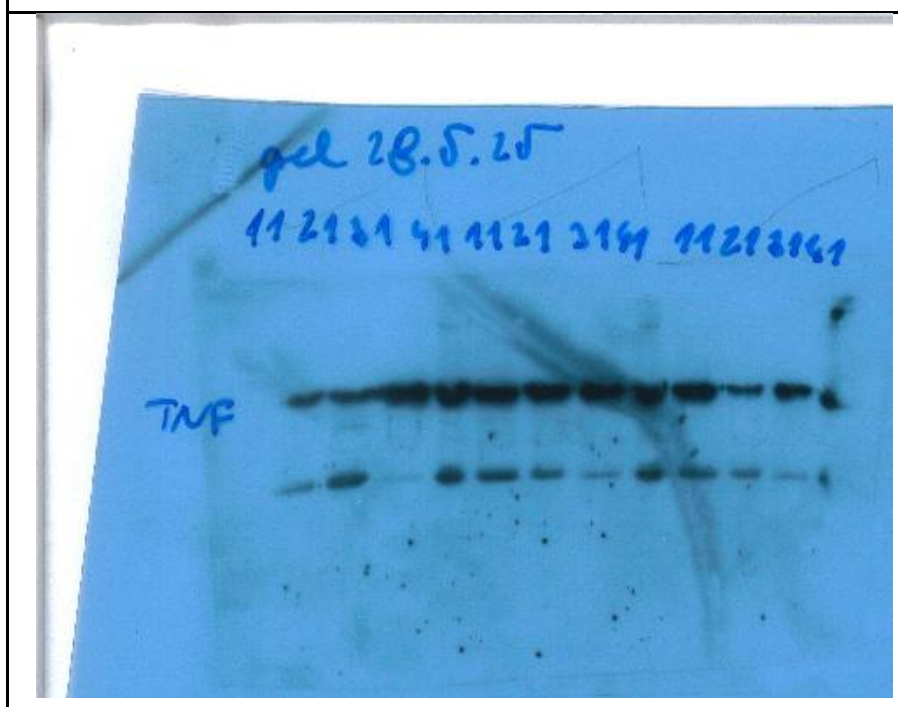	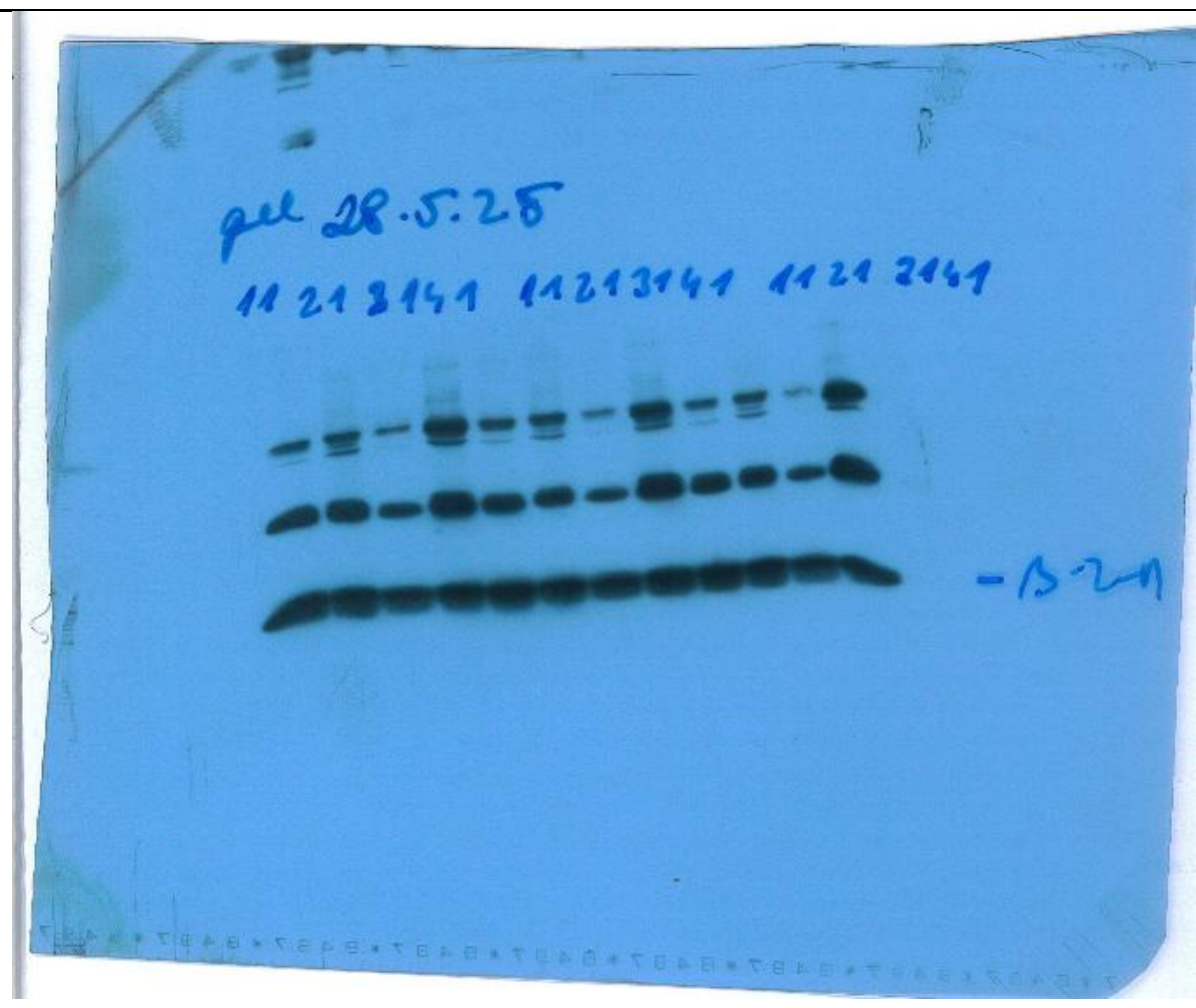
12,22,33,42-3x	

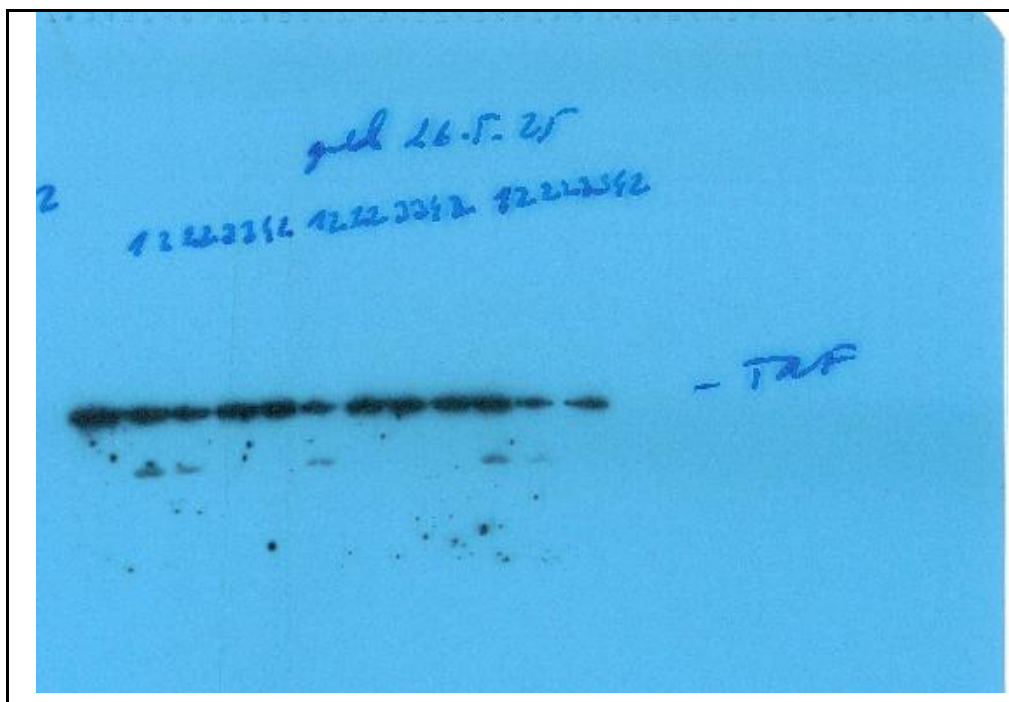	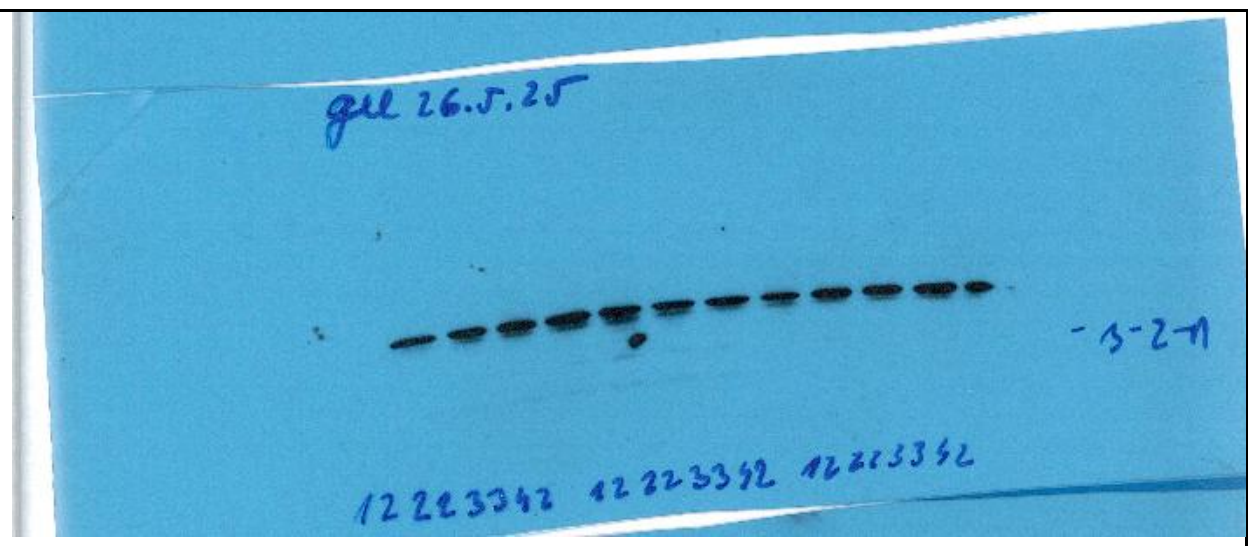
13,23,34,43-3x	
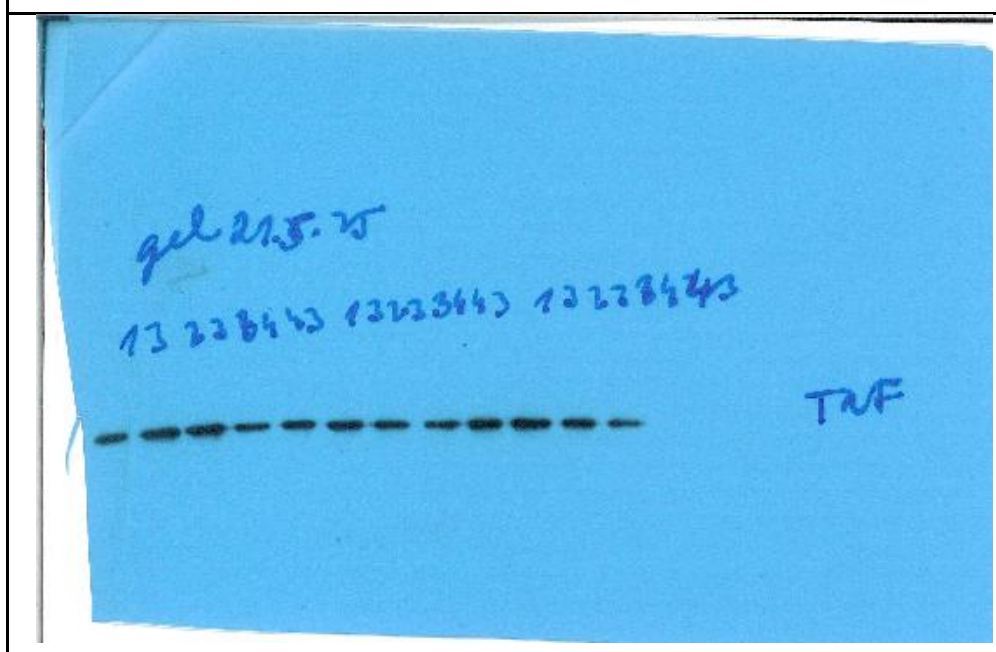	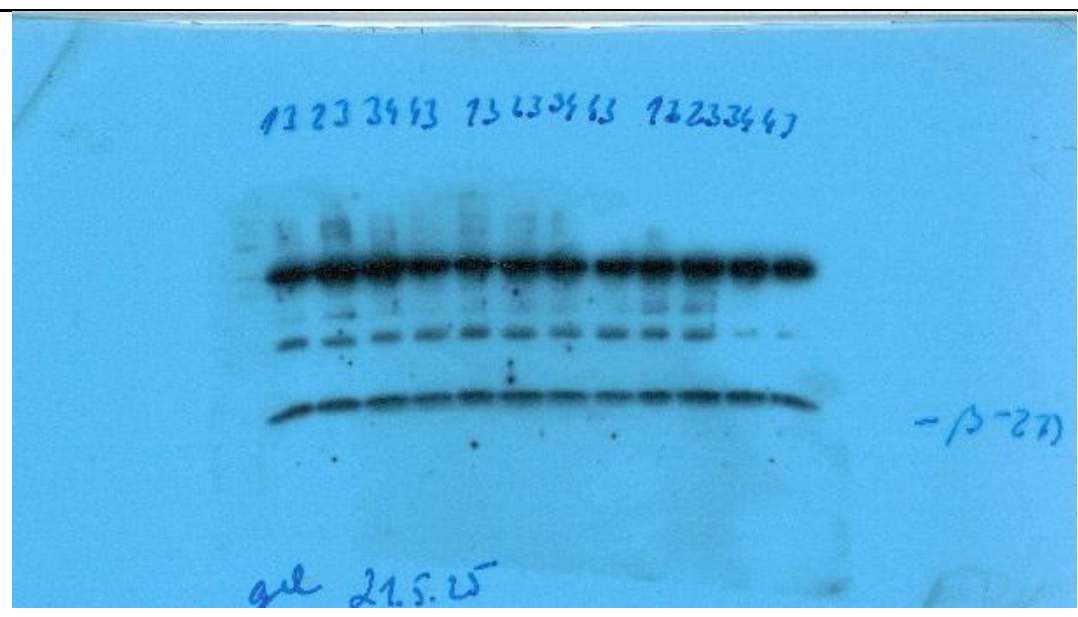
14,24,35,44-3x	
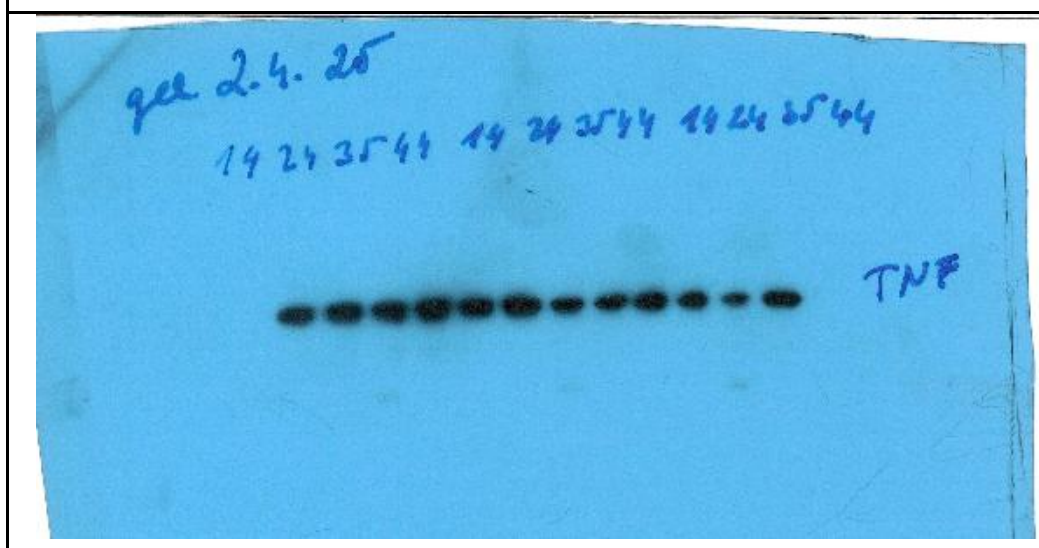	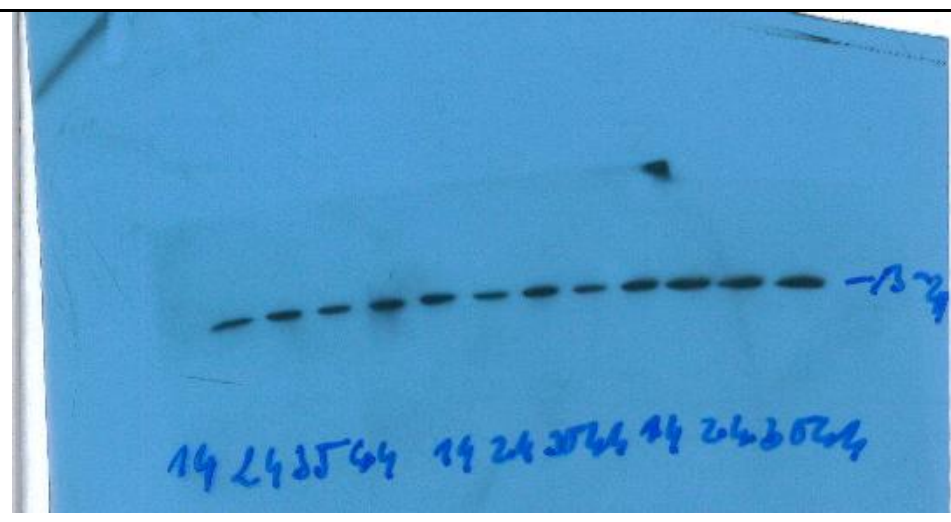
15,25,36,45-3x	
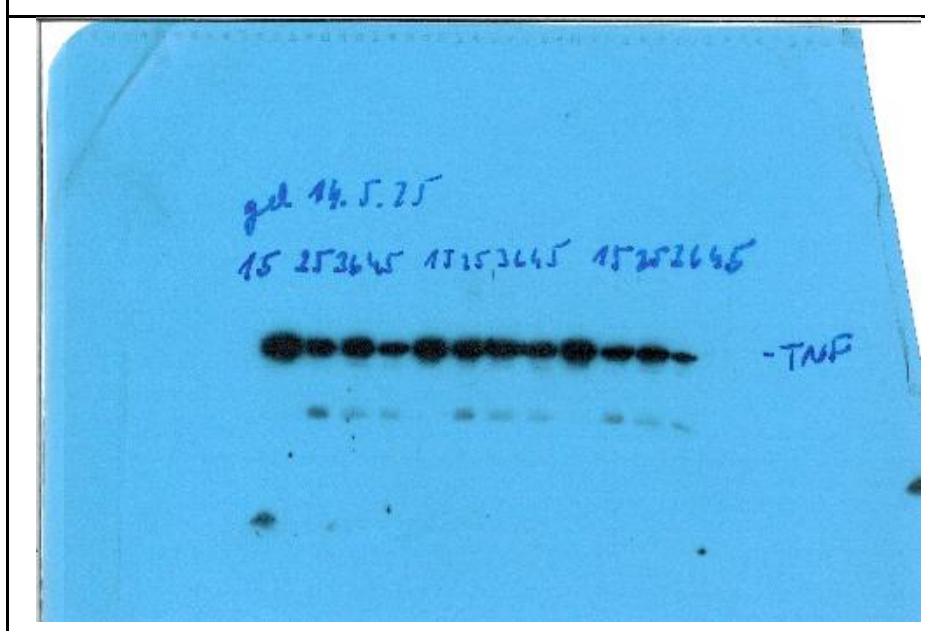	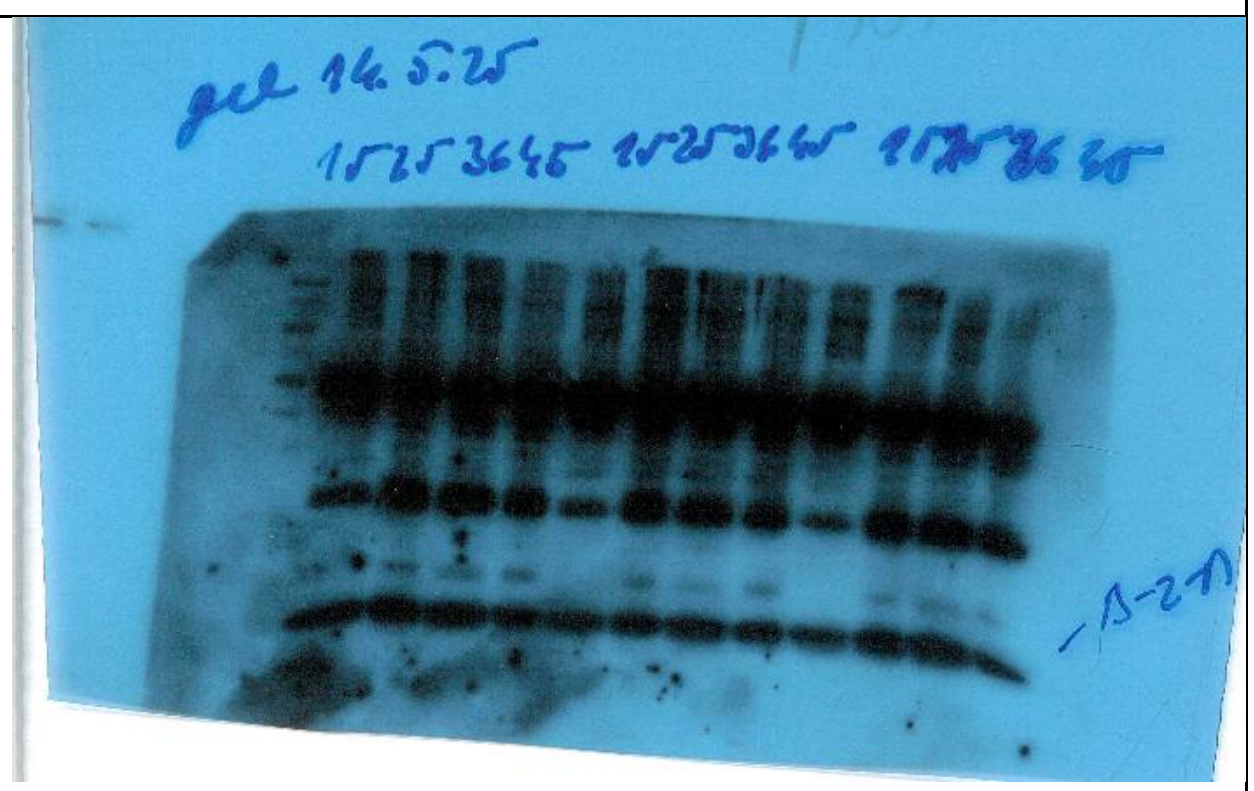
16,27,37,46-3x	

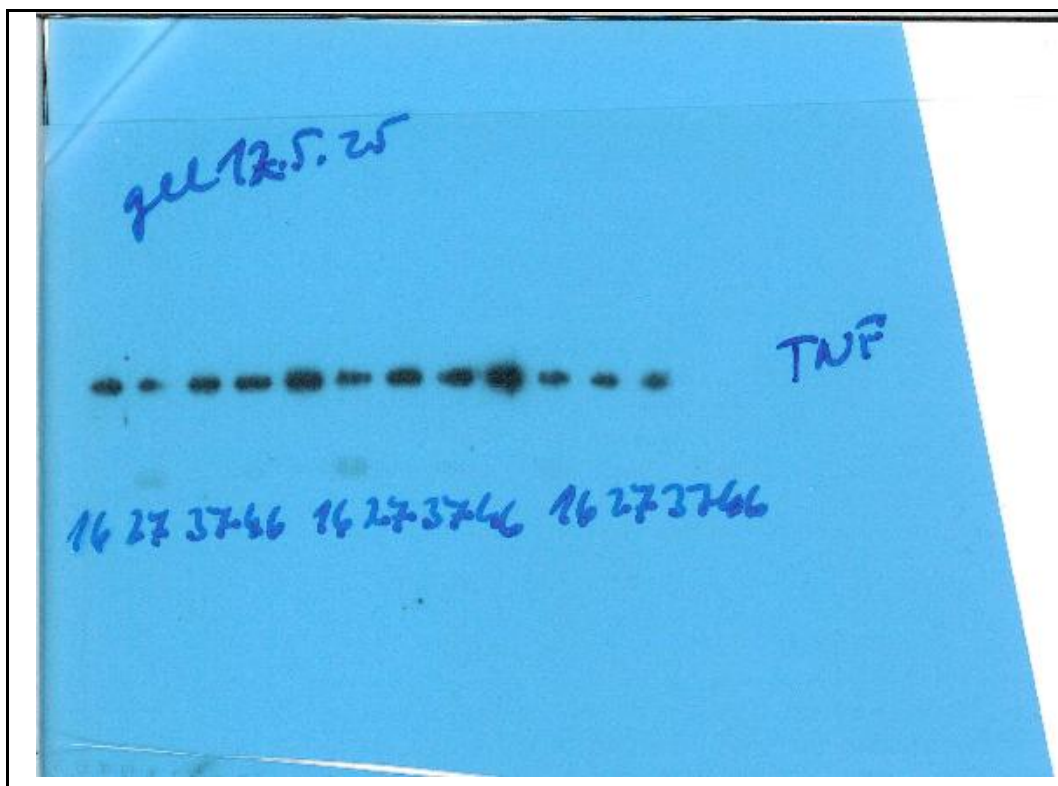	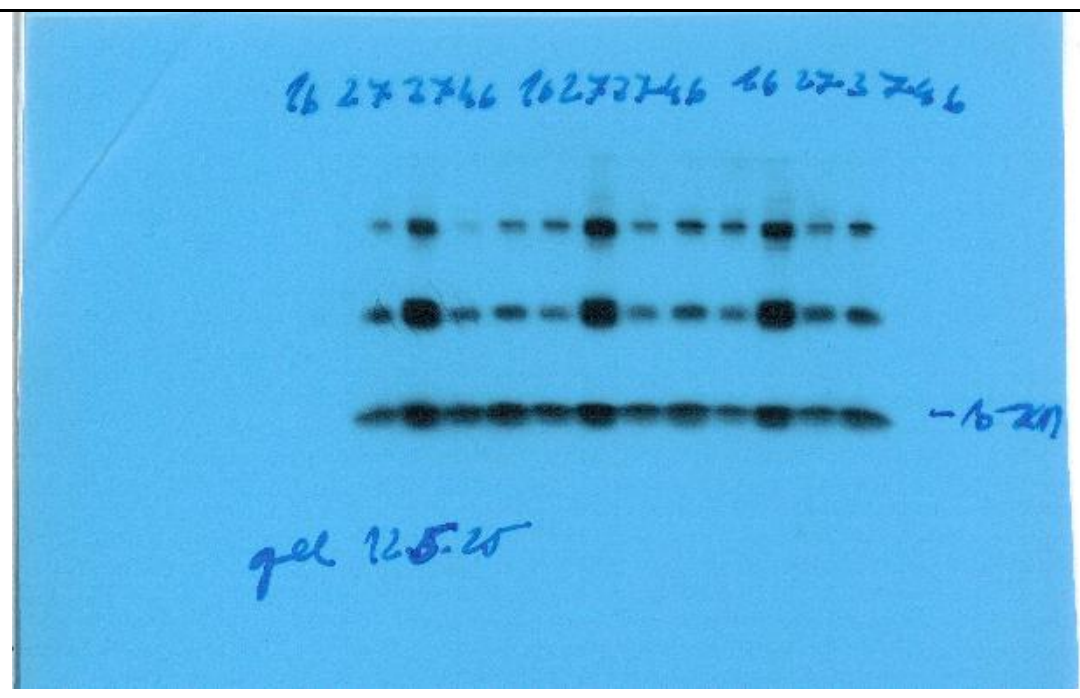
28,38,47,17-3x	
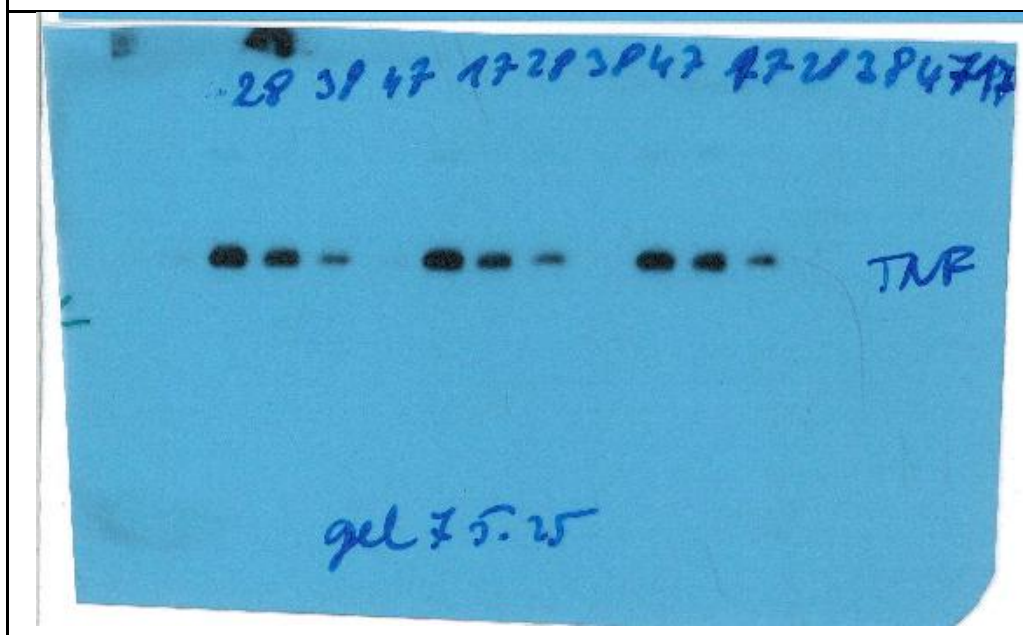	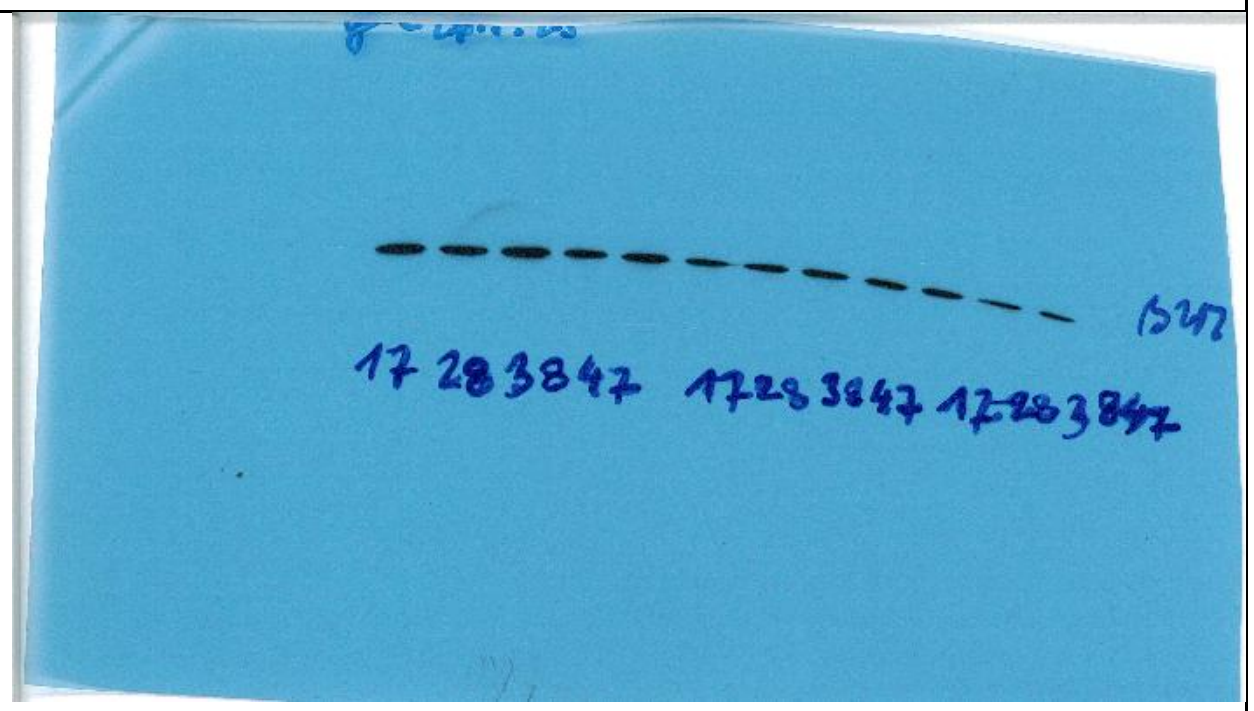
18,29,39,48-3x	
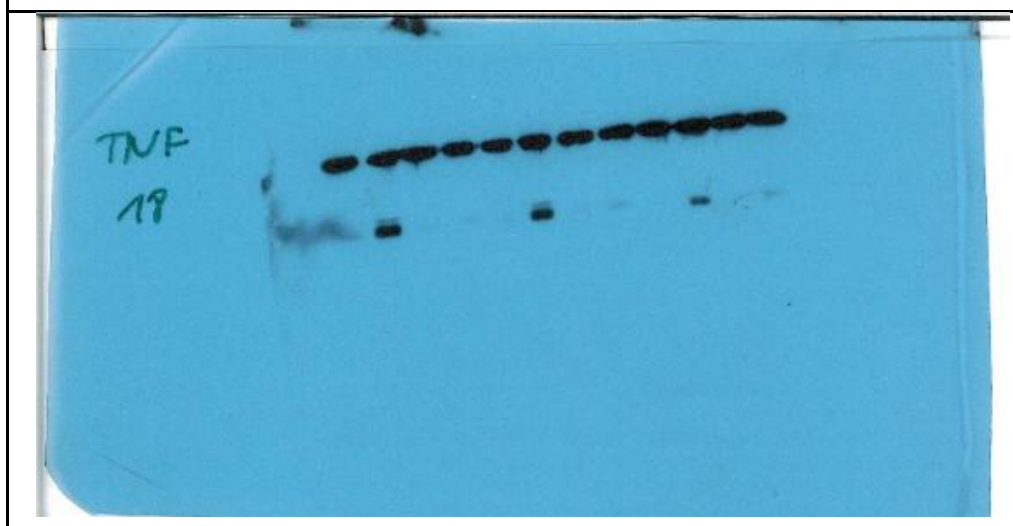	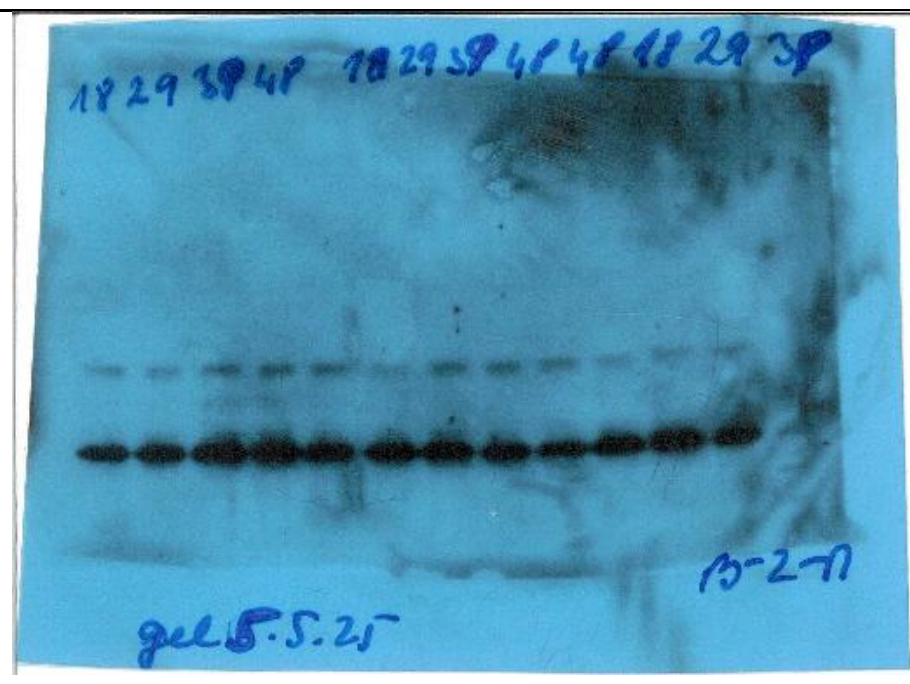
19,310,49-3x	
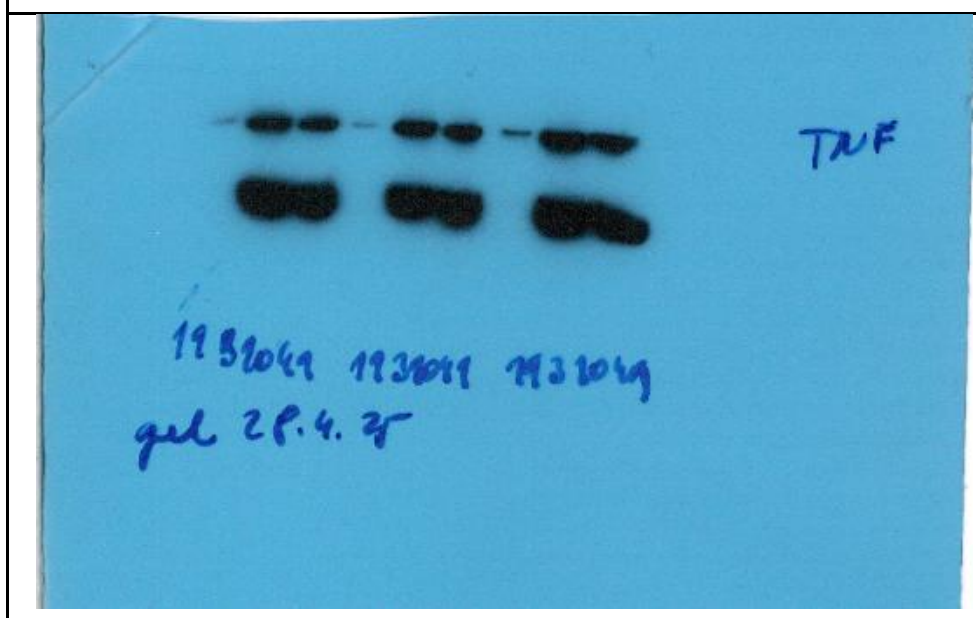	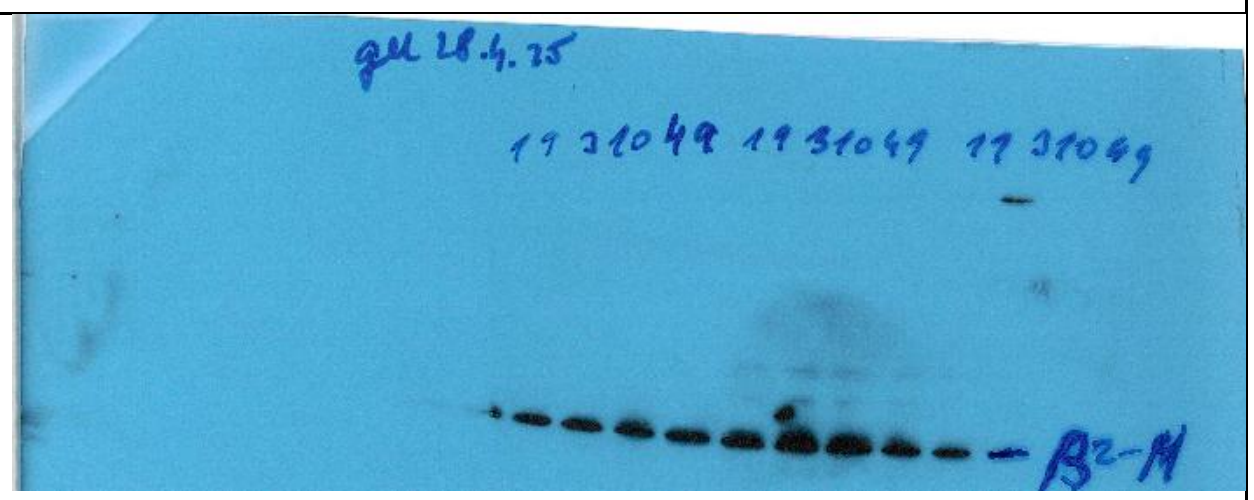

MMP-3	b-2-m
11,21,31,41-3x	
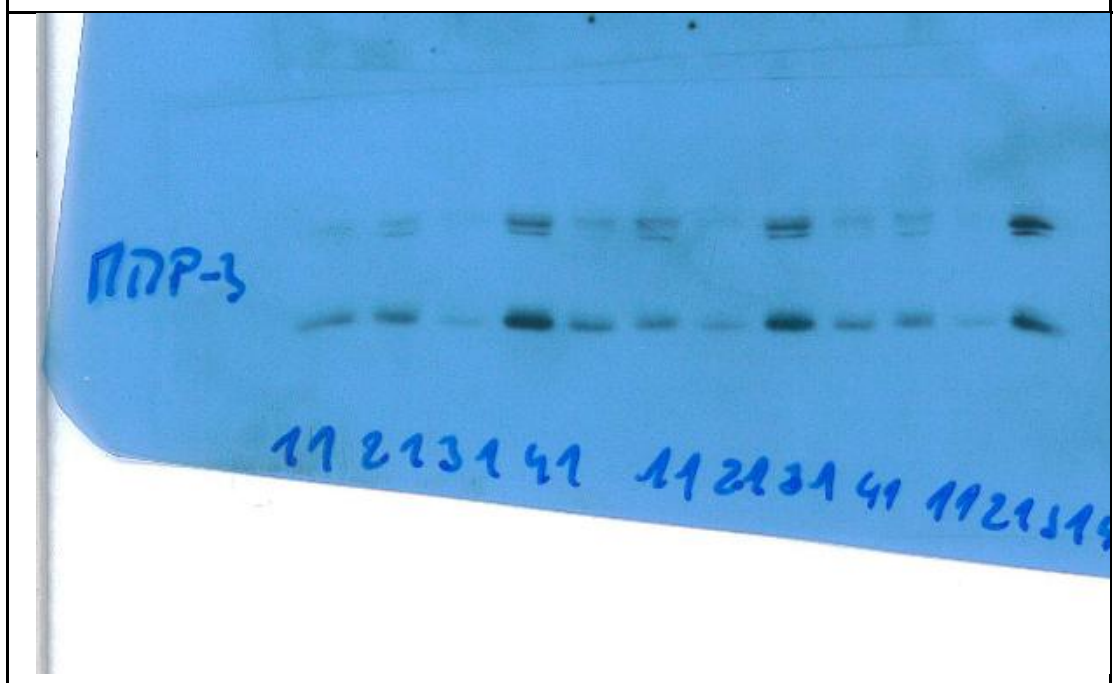	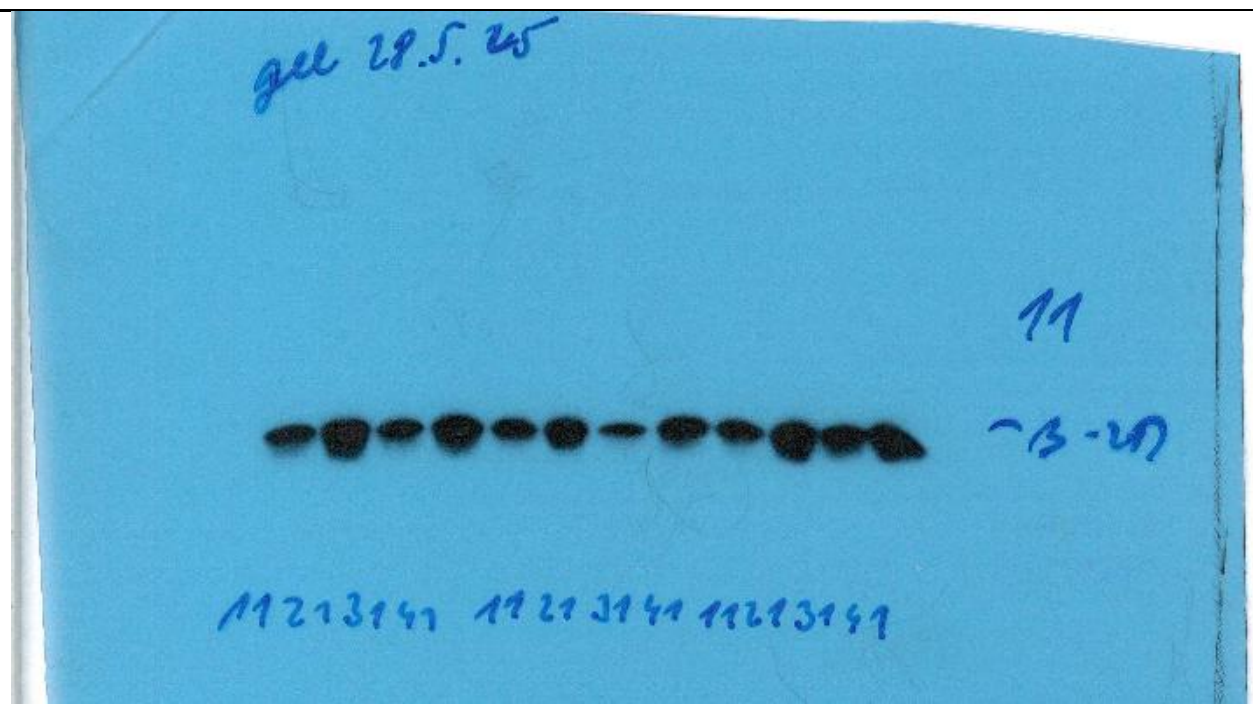
12,22,33,42-3x	
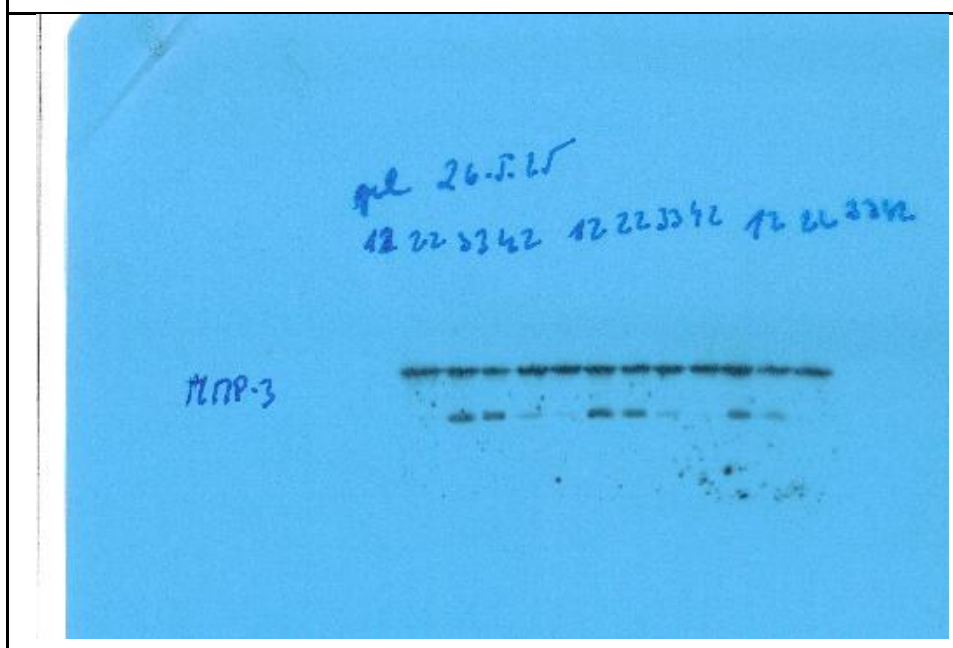	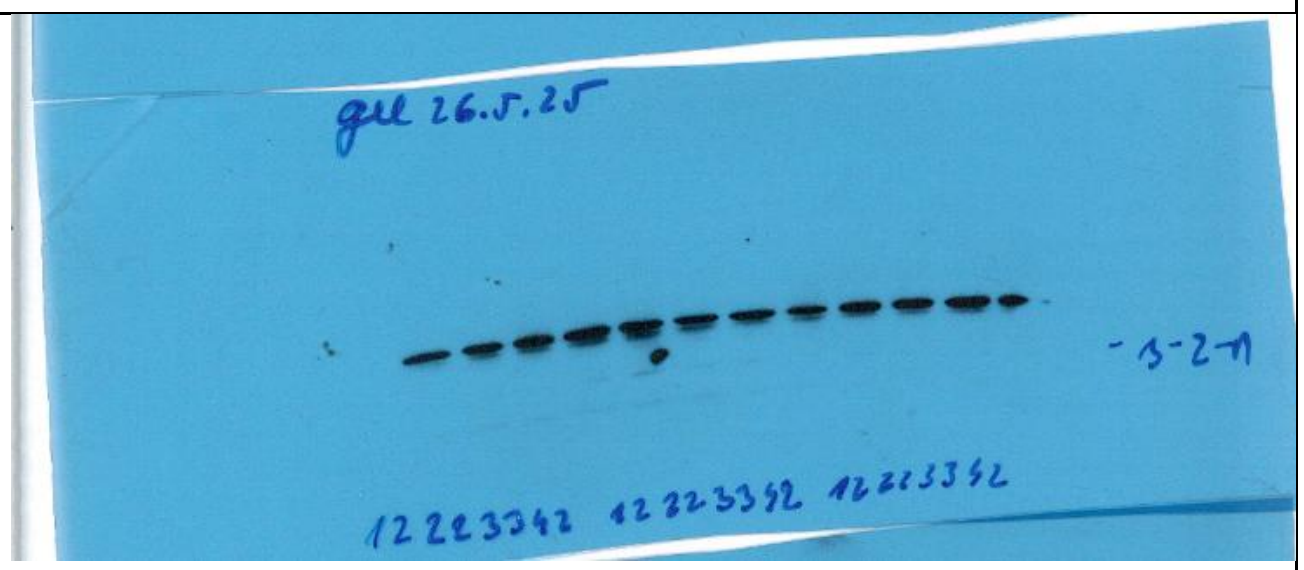
13,23,34,43-3x	
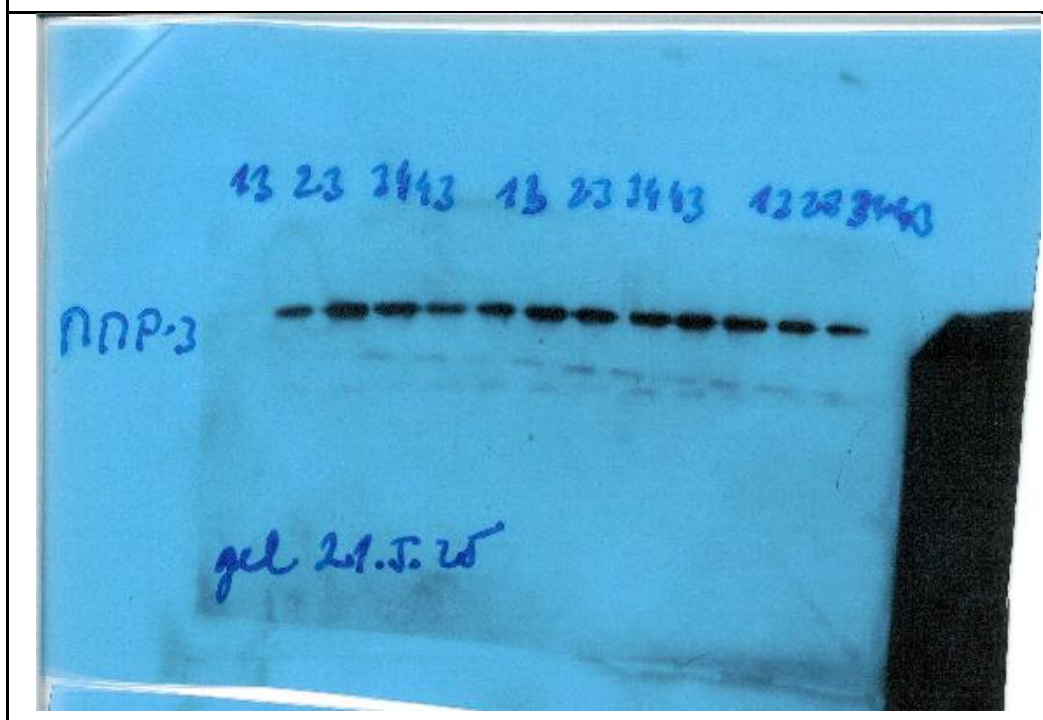	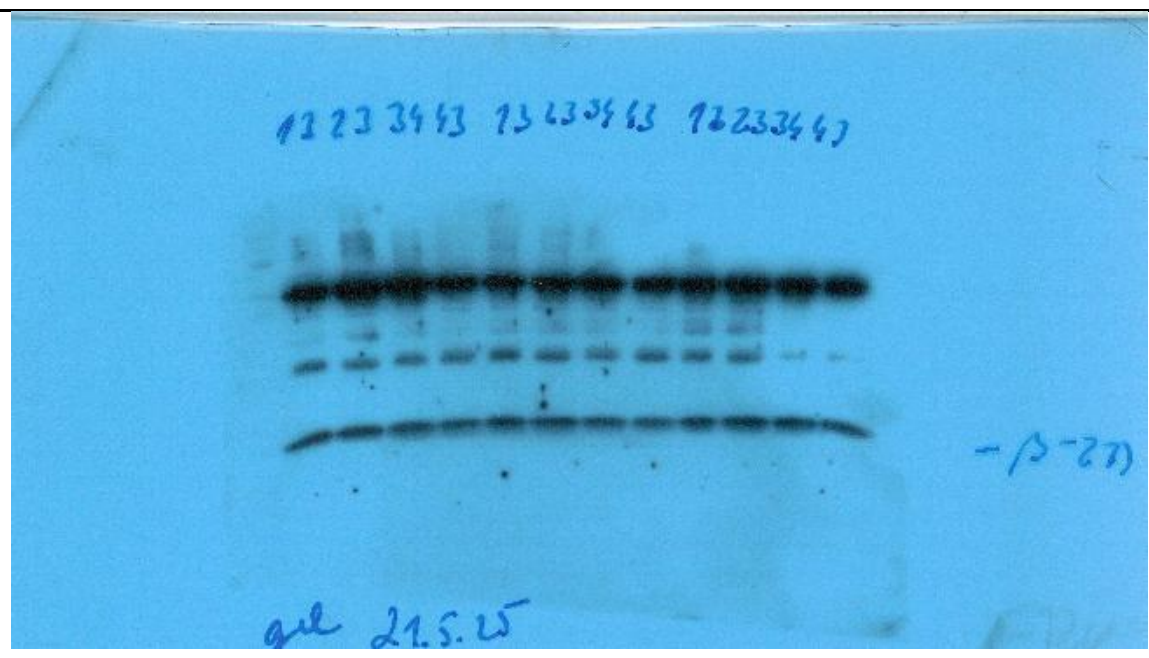
14,24,35,44-3x	
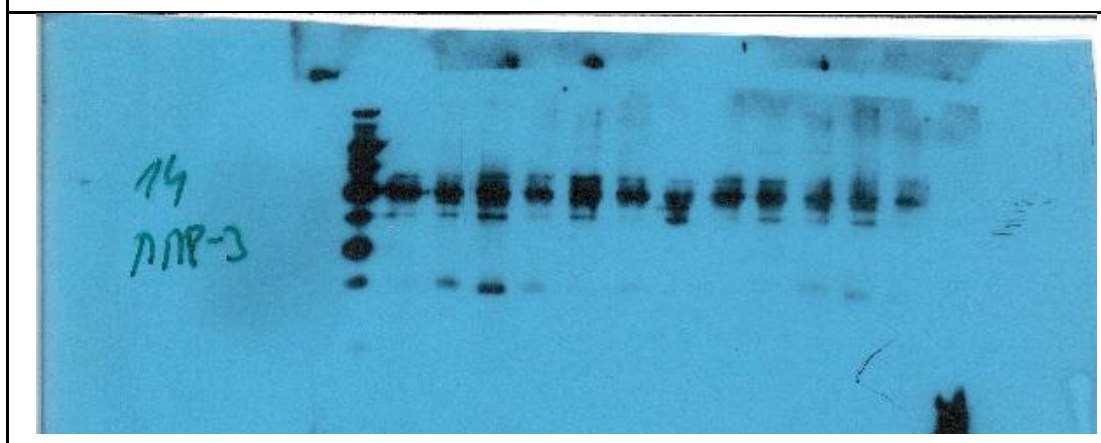	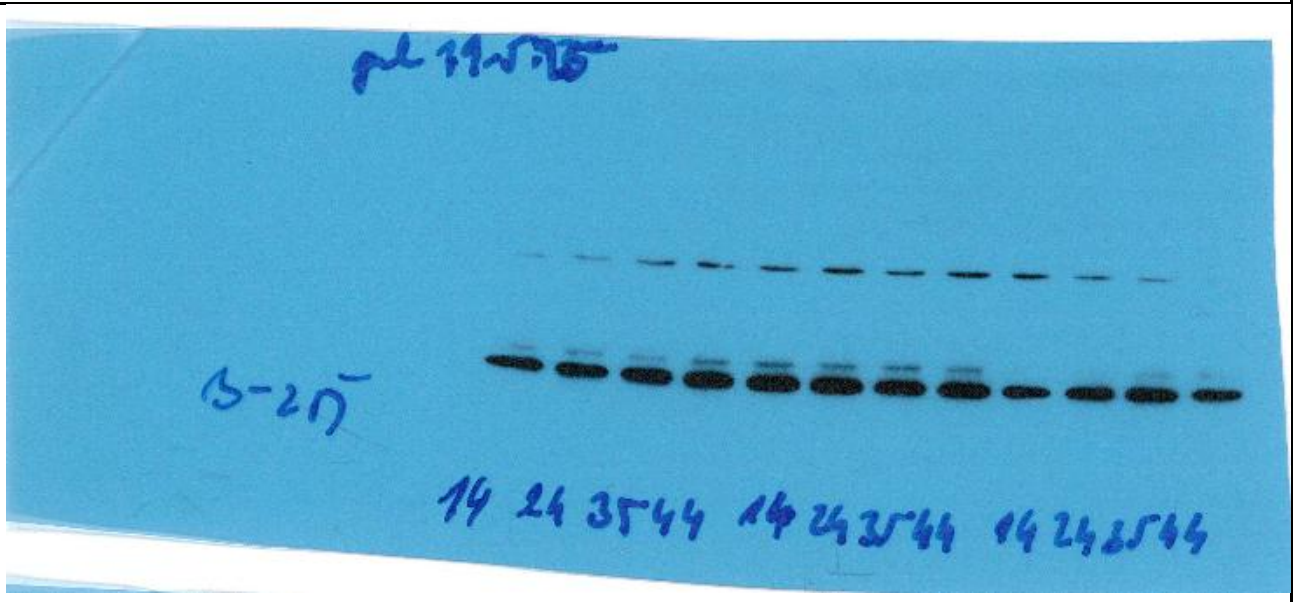

15,25,36,45-3x	
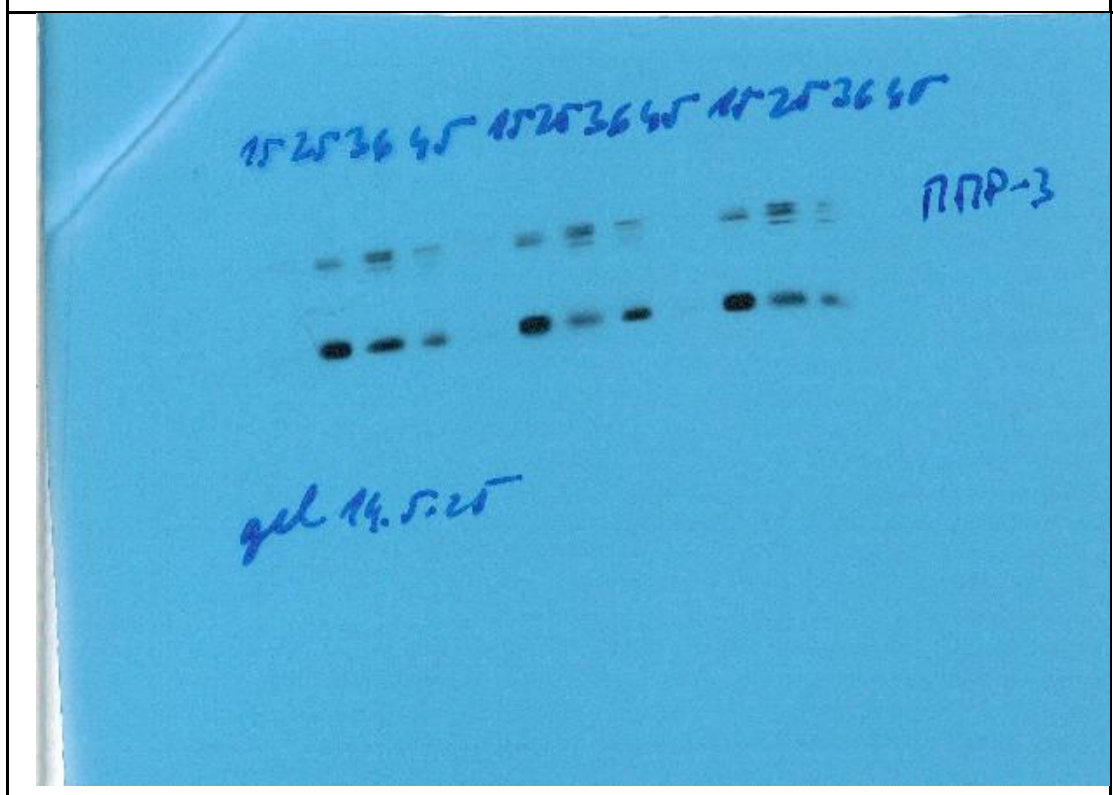	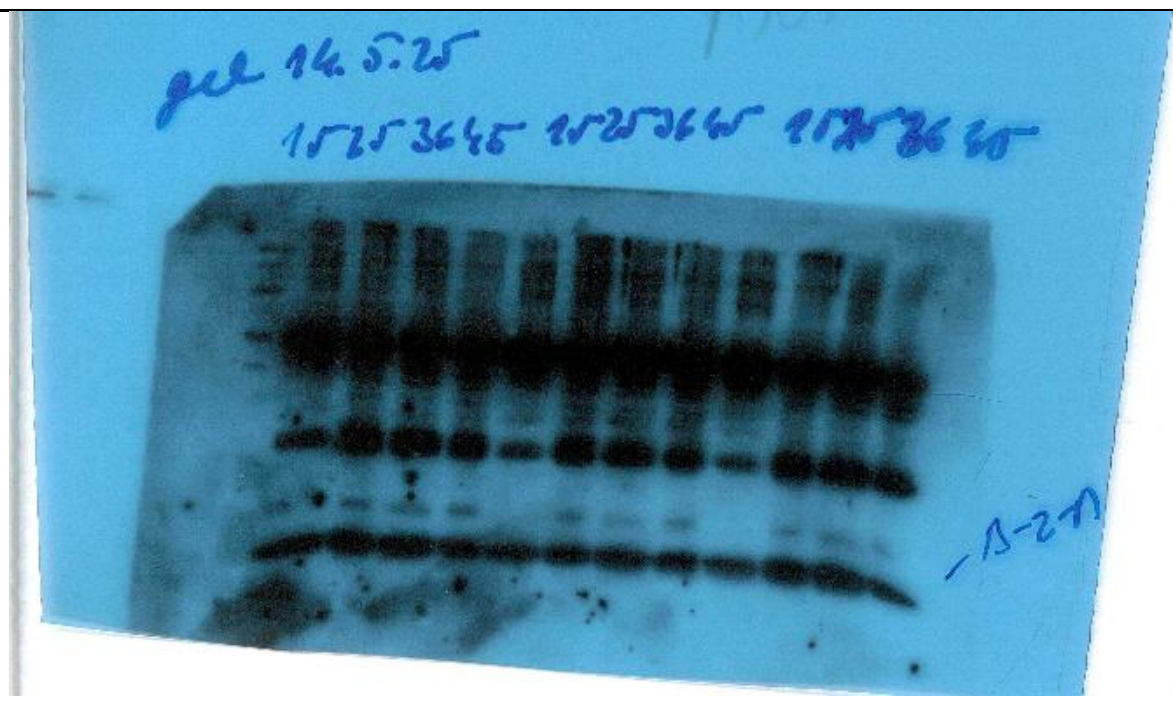
16,27,37,46-3x	
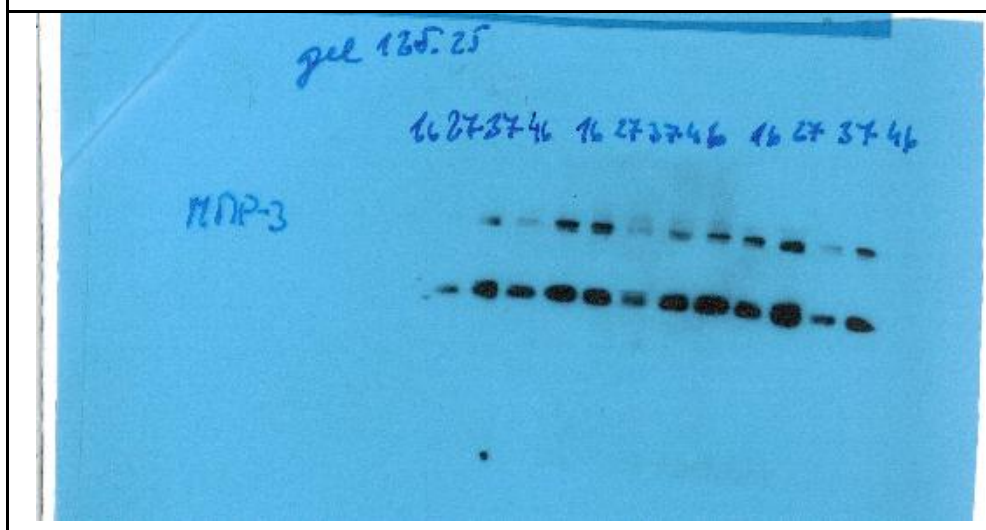	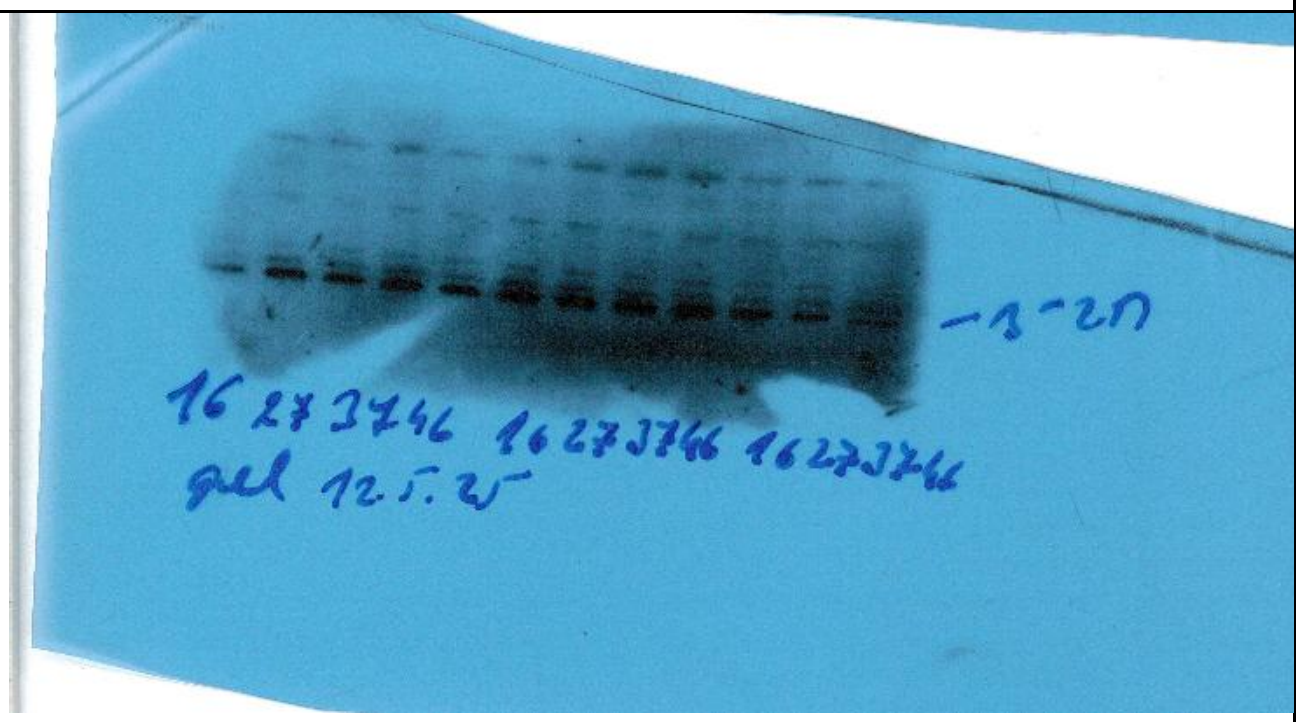
17,28,38,47-3x	
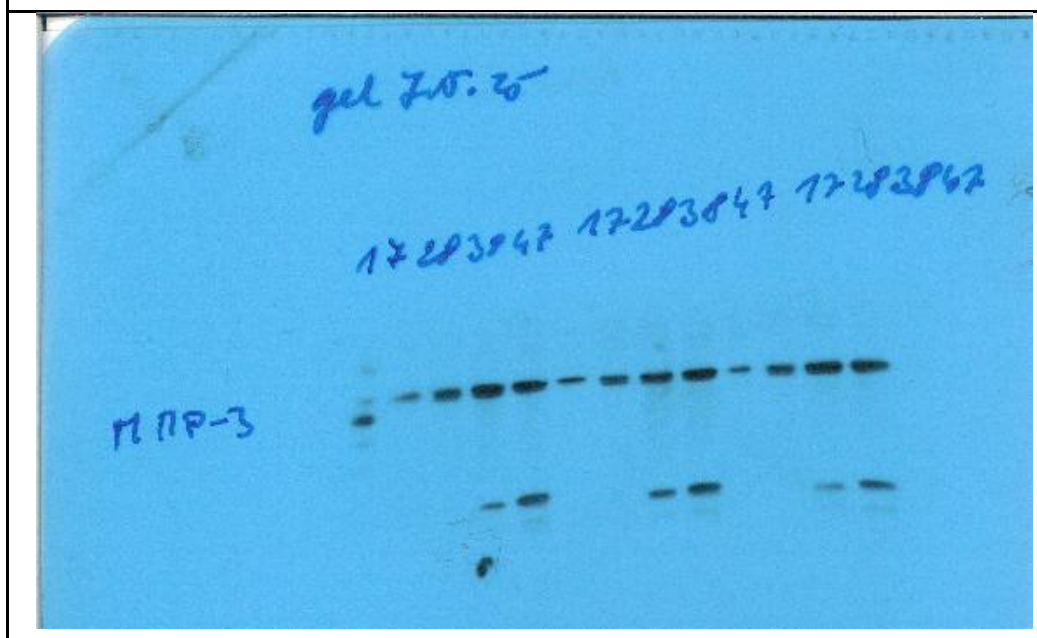	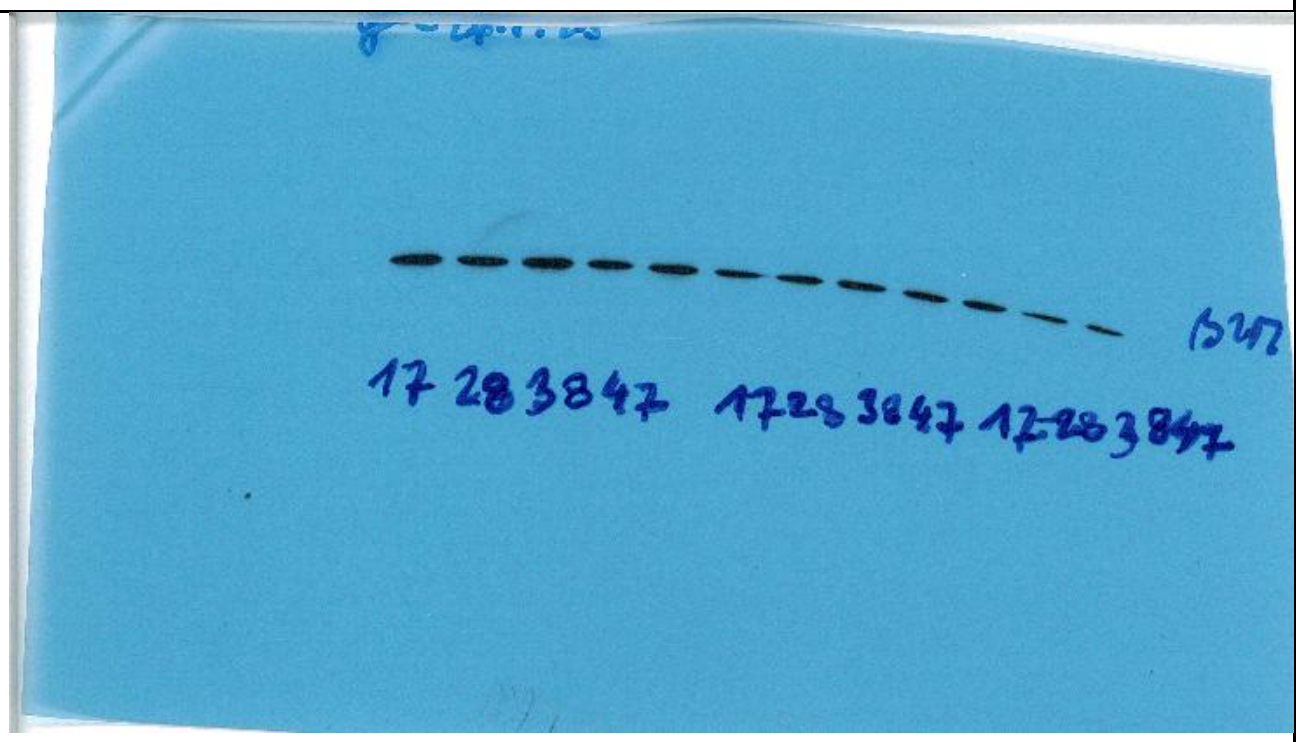
18,29,39,48-3x	

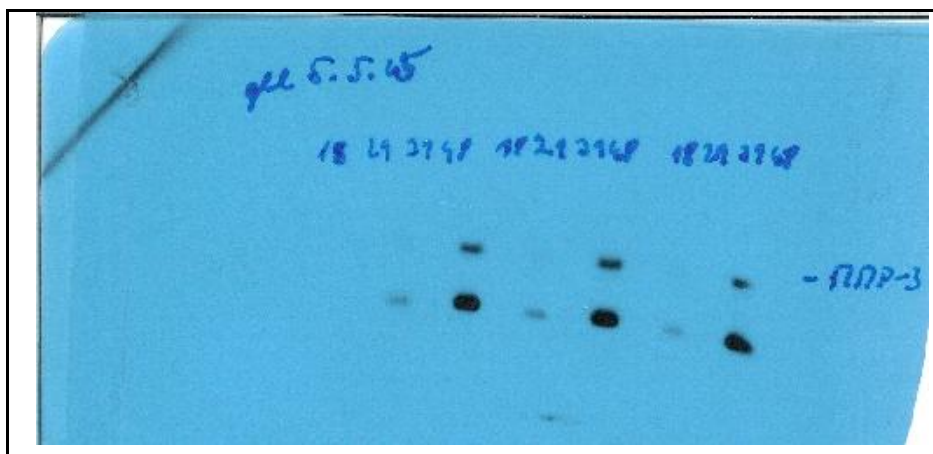	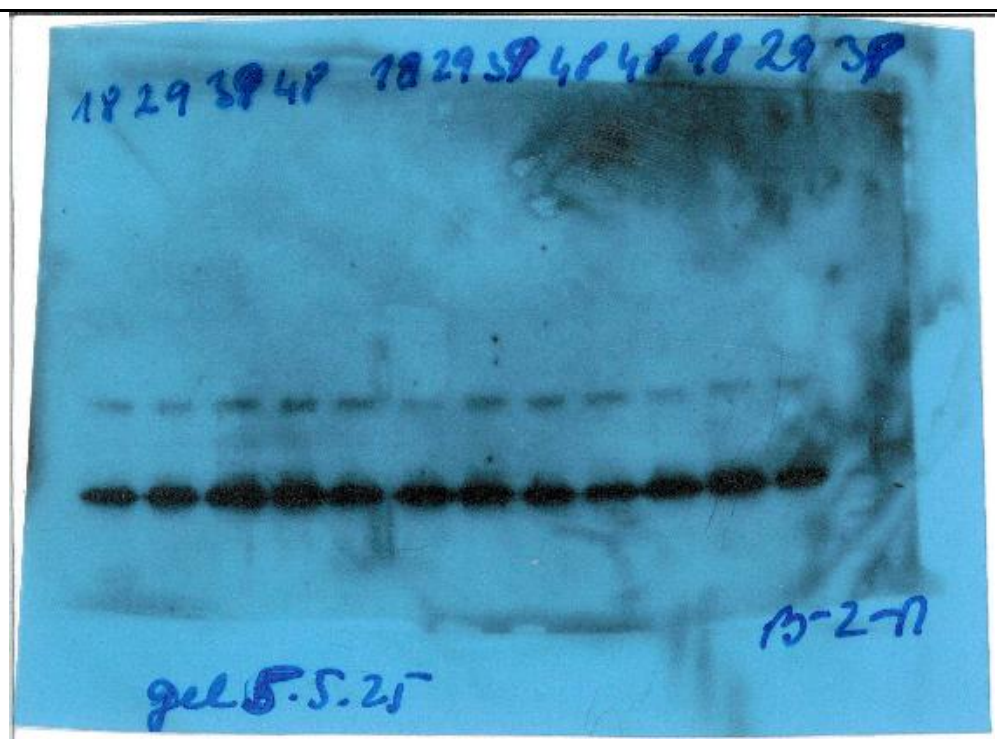
19,310,49-3x	
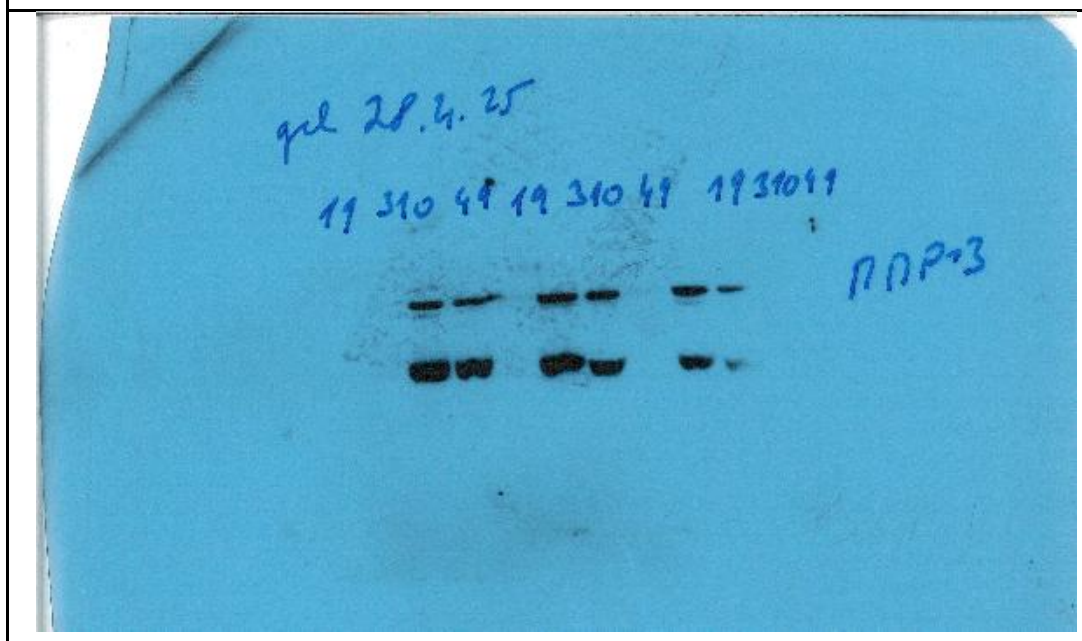	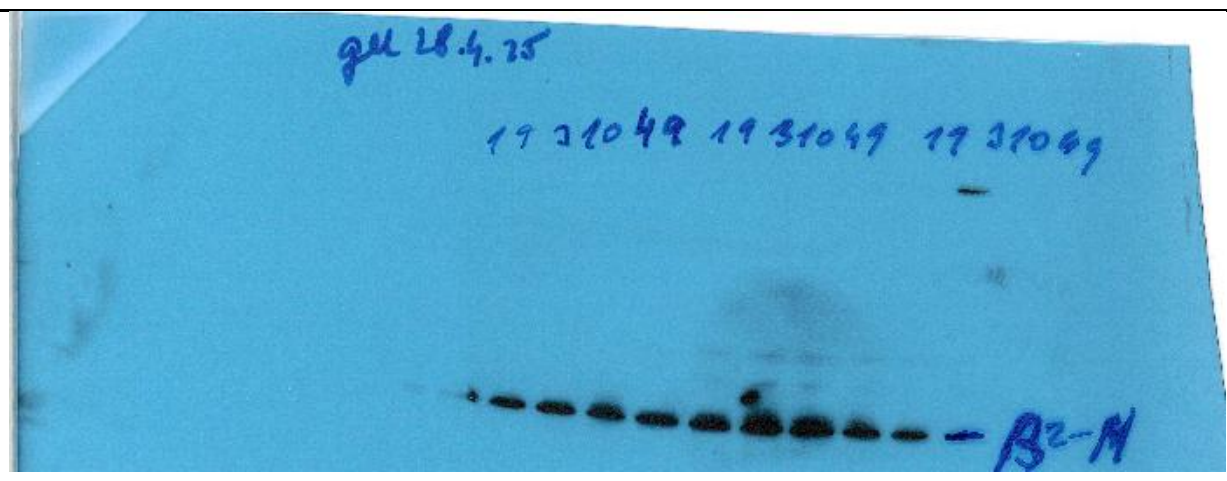
